# A small-molecule Skp1 inhibitor elicits cell death by p53-dependent mechanism

**DOI:** 10.1016/j.isci.2022.104591

**Published:** 2022-06-14

**Authors:** Muzammal Hussain, Yongzhi Lu, Muqddas Tariq, Hao Jiang, Yahai Shu, Shuang Luo, Qiang Zhu, Jiancun Zhang, Jinsong Liu

**Affiliations:** 1 State Key Laboratory of Respiratory Disease, Center for Chemical Biology and Drug Discovery, Guangzhou Institutes of Biomedicine and Health, Chinese Academy of Sciences, 190 Kaiyuan Avenue, Science Park, Guangzhou 510530, China; 2 University of Chinese Academy of Sciences, Beijing 100049, China; 3 Bioland Laboratory (Guangzhou Regenerative Medicine and Health Guangdong Laboratory), Guangzhou 510005, China; 4 Guangdong Provincial Key Laboratory of Biocomputing, Institute of Chemical Biology, Guangzhou Institutes of Biomedicine and Health, Chinese Academy of Sciences, Guangzhou 510530, China; 5 China-New Zealand Joint Laboratory on Biomedicine and Health, Guangzhou 510530, China; 6 Guangdong-Hong Kong-Macao Joint Laboratory of Respiratory Infectious Diseases, Guangzhou Institutes of Biomedicine and Health, Chinese Academy of Sciences, Guangzhou 510530, China

**Keywords:** Drugs, Pharmaceutical science, Chemistry, Organic chemistry, Biological sciences, Cell biology

## Abstract

Skp1 overexpression promotes tumor growth, whereas reduced Skp1 activity is also linked with genomic instability and neoplastic transformation. This highlights the need to gain better understanding of Skp1 biology in cancer settings. To this context, potent and cellularly active small-molecule Skp1 inhibitors may be of great value. Using a hypothesis-driven, structure-guided approach, we herein identify Z0933M as a potent Skp1 inhibitor with K_D_ ∼0.054 μM. Z0933M occupies a hydrophobic hotspot (P1) – encompassing an aromatic cage of two phenylalanines (F101 and F139) – alongside C-terminal extension of Skp1 and, thus, hampers its ability to interact with F-box proteins, a prerequisite step to constitute intact and active SCF E3 ligase(s) complexes. *In cellulo*, Z0933M disrupted SCF E3 ligase(s) functioning, recapitulated previously reported effects of Skp1-reduced activity, and elicited cell death by a p53-dependent mechanism. We propose Z0933M as valuable tool for future efforts toward probing Skp1 cancer biology, with implications for cancer therapy.

## Highlights

• Z0933M manifests strong binding with Skp1 and inhibits Skp1-F-box PPIs
• Z0933M interacts with a P1 hotspot alongside C-terminal extension of Skp1
• Z0933M alters SCF E3 ligase functioning, leading to substrate accumulation/modulation
• Z0933M causes cell-cycle arrest, and elicits cell death by p53-dependent mechanism

## Graphical abstract

**Figure:**
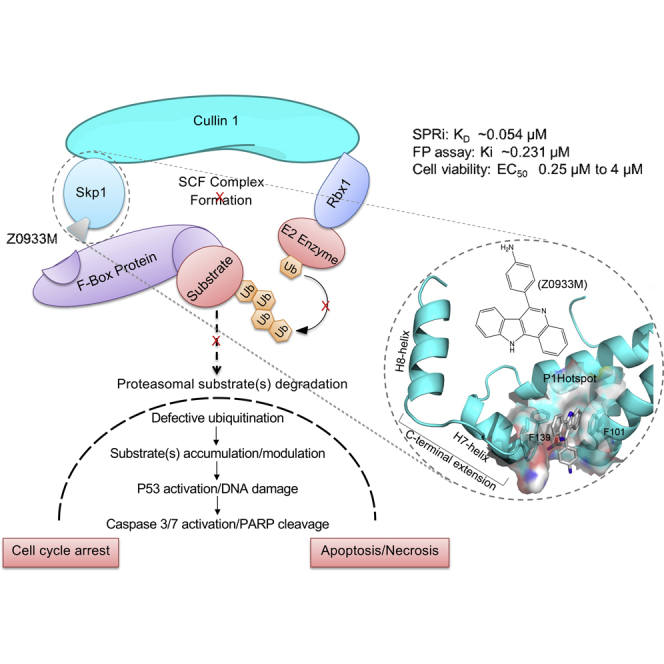

## Introduction

S phase kinase-associated protein 1 (Skp1) is a 163-amino-acid protein that is ubiquitously expressed in different cells and tissues (Hussain et al., 2016). Structurally, Skp1 protein has ∼125 residue N-terminal domains with α/β structures, and a two-helix C-terminal extension (Figure S1) (Hussain et al., 2016). Encompassing a binding interface, the C-terminal extension mediates protein-protein interactions (PPIs) of Skp1 with various members of the F-box protein family (∼69), a prerequisite step to constitute a repertoire of intact and active multi-subunit SCF (Skp1-Cullin 1(Cul1)-F-box) E3 ubiquitin ligase(s) and ncPRC1.1 (non-canonical Polycomb Repressive Complex 1.1) assemblies (Hussain et al., 2016; Wong et al., 2016). Skp1, thereof, serves as an essential component of – and thereby facilitates proteasomal degradation of myriad substrates as well as the chromatin regulation, respectively, by – SCF E3 ligase(s) and ncPRC1.1 complexes (Cohen et al., 2018; Hussain et al., 2016; Vidal and Starowicz, 2017; Wang et al., 2018). Moreover, Skp1 through C-terminal extension also collaborates with some non-F-box proteins like CENP-E (centromere-associated protein E) and stratifen (SFN, 14-3-3 sigma), contributing to their respective functioning in cytokinesis and tumorigenesis (Liu et al., 2006; Shiba-Ishii et al., 2019). Given these facts, one may indeed infer that Skp1 partakes in many aspects of cellular biology. However, at this time, the cellular functions of Skp1 in normal physiology and beyond are yet to be comprehensively elucidated.

Meanwhile, there are conflicting reports which implicate aberrant expression of Skp1 in oncogenesis and genome instability. On one hand, separate lines of inquiry have documented that Skp1 overactivation or overexpression promotes cancer stemness, cancer initiation, and progression, and correlates with poorer patient outcome as well (Liu et al., 2015; Shiba-Ishii et al., 2019; Tian et al., 2020; Wu et al., 2021; Zhu et al., 2021). In addition, *Skp1* depletion using genetic methods or Skp1 pharmacological inactivation reduces cancer cell viability or growth (Liu et al., 2015; Tian et al., 2020; Zhu et al., 2021). But, on the other hand, attenuation of *Skp1* activity in some cancer cells has been linked to increased genomic instability, replication stress, DNA double-strand breaks (DSB), and neoplastic transformation (Lepage et al., 2021; Piva et al., 2002; Thompson et al., 2020). From a mechanistic perspective, the modulated functioning of tumor promoter (such as cyclin E, c-Myc, cyclin D1, c-Jun, etc.) or suppressor (e.g. RASSF1) proteins, which are *bona fide* substrates of SCF assembly, appears to be puppeteer of Skp1 perpetration in either case (Shiba-Ishii et al., 2019; Tian et al., 2020; Wu et al., 2021). In short, the enigmatic interplay of these conjoined yet opposed effects raises the possibility that molecular axes of Skp1 partnership in the cellular machinery could fine-tune and maintain cellular homeostasis of tumor suppressor/promoter proteins. Should the balance of this partnership tip over in favor of tumor promoters, the tumorigenic potential will rise and vice versa. With this notion in mind, potent and cellularly active small-molecule Skp1 inhibitors as a chemical tool to explicate Skp1 biology will be of great value, not only to understand as-yet unexplored cellular functioning but also for comprehensive validation of Skp1 as a therapeutic target. However, no considerable progress toward small-molecules targeting Skp1 has been reported so far.

**Figure 1:**
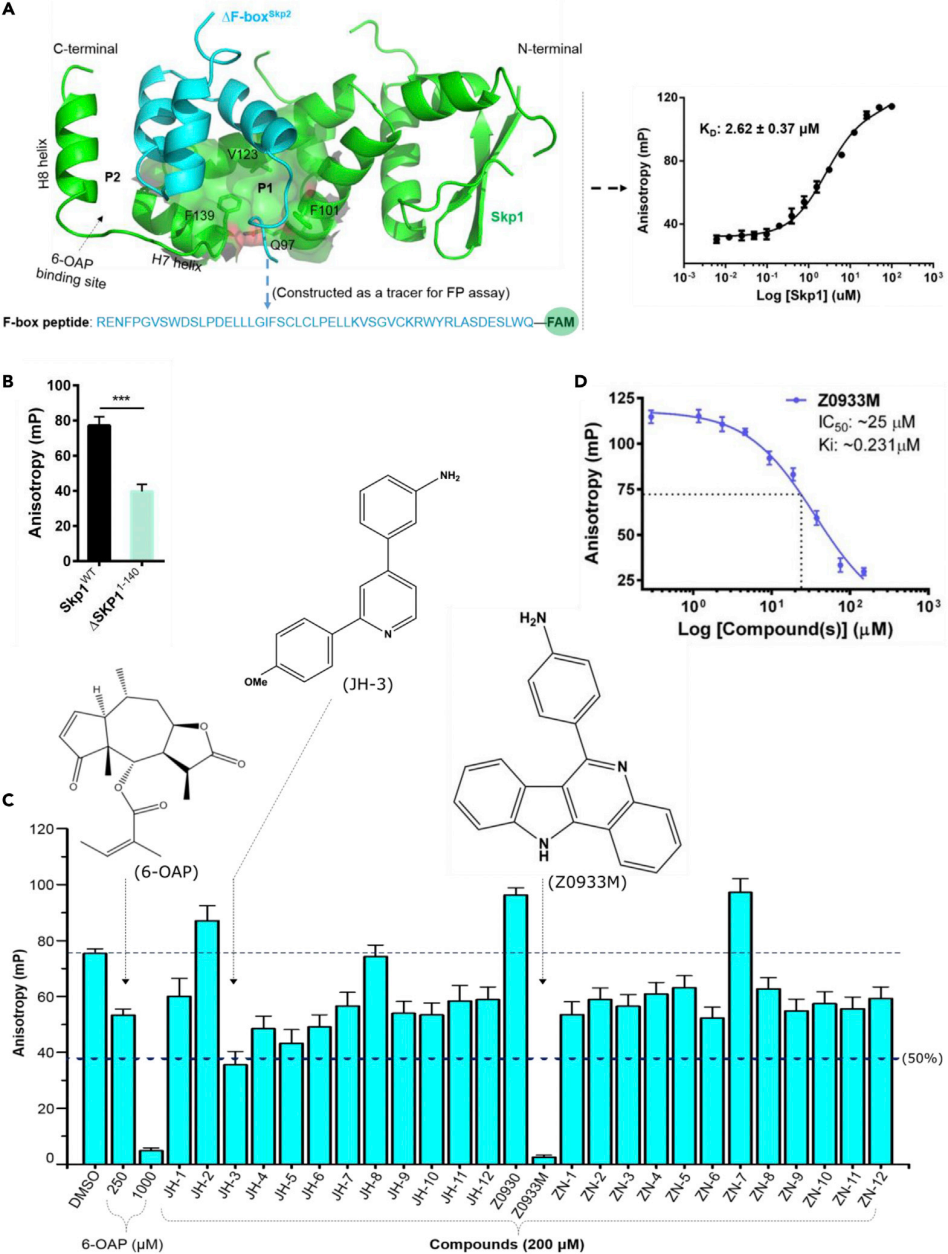
Biochemical identification of Z0933M as a potent inhibitor of Skp1-F-box PPIs (A) Design strategy for the FP-based *in vitro* competition assay, based on the crystal structure of Skp1-Skp2 (PDB: 2AST) (left), and the *in vitro* titration curve of Skp1^WT^ and the constructed ΔSkp2^F^-^box^ peptide giving a K_D_ value of 2.62 ± 0.37 μM (right). (B) The ΔSkp2^F^^-^^box^ peptide showed significantly reduced binding with ΔSkp1^1−140^ (see also Figure S3C), suggesting that it specifically binds at the F-box-binding interface of Skp1. Data are presented as the mean ± SD (*n* = 3). ∗∗∗*p* < 0.001, two-tailed Student’s *t* test. (C) Bar graph representation for the inhibitory effect(s) of compounds tested by the FP-based *in vitro* competition assay. (D) Dose-response kinetic analysis for compound Z0933M by FP assay.

The only notable exception relies on 6-OAP (Figure 1) (Liu et al., 2015), the first molecule reported previously by a joint venture of our group. 6-OAP was identified by *in silico* screening approach and demonstrated to bind at H8-helix region (P2) of the C-terminal extension, thereby disrupting Skp1-F-box PPIs (Liu et al., 2015). However, 6-OAP possesses moderately potent cellular activity and insufficient selectivity (Cheng et al., 2017; Liu et al., 2015). Moreover, the difficult-to-modify chemical structure of 6-OAP has impeded the precise exploration of its structure–activity relationships (SAR), and the efficient development of more potent compounds. This highlights the need for identifying new inhibitor chemotypes that could potentially be exploited from drug discovery prospects against Skp1.

Taking the efforts to next stage, we herein took advantage of a hypothesis-driven, structure-guided biochemical approach to identify Z0933M as a cellularly more active Skp1 inhibitor as compared to 6-OAP. By using a series of *in vitro* and cell-based experiments, we show that Z0933M engages the P1 aromatic hydrophobic hotspot alongside the C-terminal extension of Skp1 to block its interaction with F-box proteins. We also demonstrate that Z0933M acts as a functional inhibitor and/or modulator of SCF E3 ligase(s) activity and, in turn, elicits cancer cell death by p53 activation, a killing mechanism that previously has not been manifested for 6-OAP. Our findings propose Z0933M as a chemical tool compound to dissect biochemical events of Skp1 functioning in cancer biology, and a potential lead scaffold for the development of new cancer therapeutics.

## Results

### Biochemical identification of Z0933M as a potent inhibitor of Skp1-F-box PPIs

The rationale behind the identification of Z0933M was garnered from our currently ongoing initiative to chemically exploit an aromatic cage of two phenylalanines (F101 and F139) for small-molecule drug discovery against Skp1. These phenylalanines constitute the outlet of a shallow but well-defined hotspot region, previously defined as pocket 1 (P1) (Liu et al., 2015) located alongside H7-helix of the C-terminal extension (see Figures 1A and S1). Indebted to this aromatic cage and the key residues in vicinity (such as Q97 and V123), P1 renders a docking site for many F-box proteins through π-π, electrostatic, and van der Waals interactions (Figure S1). Our preliminary investigations involving co-immunoprecipitation (co-IP) and cell-based FRET (Fluorescence Resonance Energy Transfer) assays indicated that site-directed mutations of P1 residues could either partly or completely diminish Skp1-F-box PPIs (Figure S2). On the basis of these experimental insights, we hence speculated that small-molecule ligands with bi- or multi-cyclic heteroaromatic architecture might have the structural potential to trammel the aromatic cage phenylalanines and, thus, hamper the ability of Skp1 to bind with F-box proteins.

To directly test our hypothesis, we subjected a small, but focused, subset of multicyclic ligands to a fluorescence polarization (FP)-based *in vitro* competition assay (Figures 1, S3, and S4). In brief, we generated an FP assay-based molecular surrogate system recapitulating Skp1-F-box PPIs (see STAR Methods, Figures 1A, 1B and S3), and then used this assay as our primary screening platform for compound testing (Figure 1C). We were able to recombinantly purify intact (Skp1^WT^) and truncate (ΔSkp1^1−140^; lacking H8-helix) forms of Skp1, and by titration experiments, to characterize the specific binding of ΔSkp2^F^-^box^ peptide (constructed on the consensus sequence of F-box domain of Skp2) at the F-box binding interface of Skp1 (Figures 1A, 1B and S3C). The previously reported Skp1 inhibitor, 6-OAP, could dose-dependently inhibit the interaction between Skp1^WT^ and ΔSkp2^F^^-^^box^ peptide, indicating the feasibility of utilizing this assay for compound screening (Figure 1C). At the initial concentration tested (200 μM), several compounds were able to inhibit the Skp1-ΔSkp2^F−box^-peptide interaction with a similar magnitude of effect as was observed with 6-OAP. Especially, the compound JH-3 caused more than 50% inhibition, whereas Z0933M almost completely disrupted the Skp1-ΔSkp2^F−box^-peptide binding, hence demonstrating several folds better inhibitory effect as compared to 6-OAP. From the subsequent dose-response kinetic analysis, Z0933M emerged as a potent inhibitor of Skp1/F-box-peptide interaction with a Ki value of 231 ± 18 nM (Figure 1D).

### Biophysical, *in silico*, and site-directed mutagenesis studies manifest Z0933M binding at P1 hotspot of Skp1

**Figure 2:**
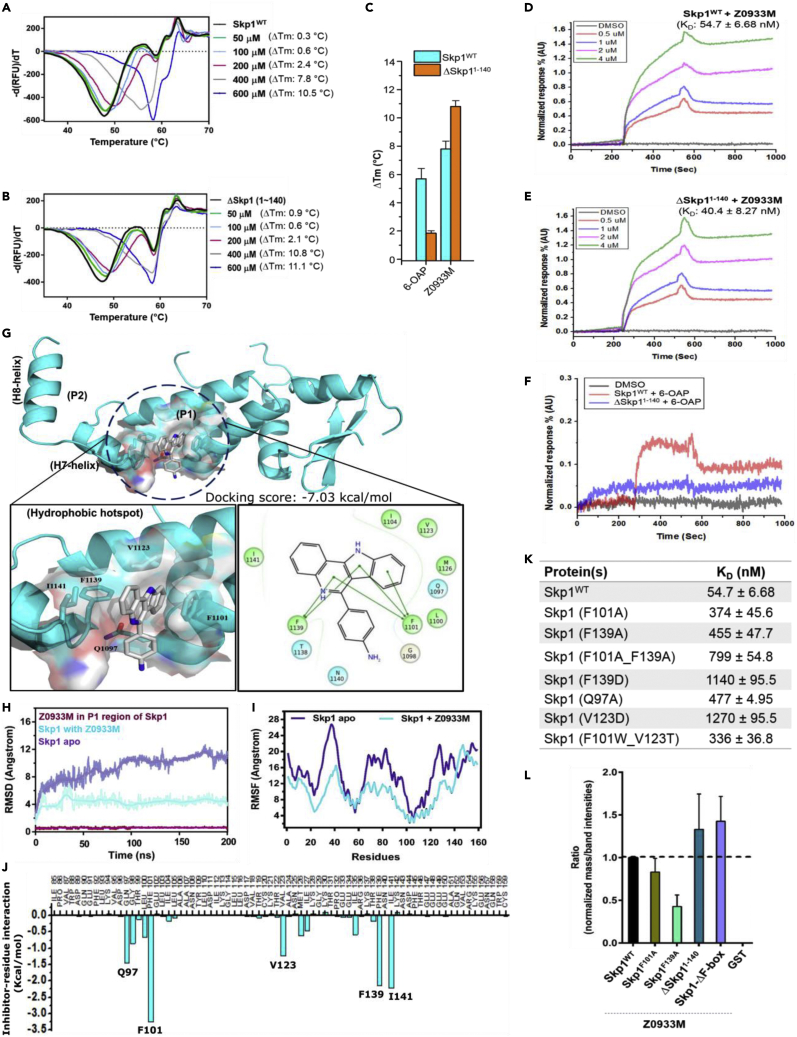
Assessment of Z0933M interaction with Skp1 by biophysical, *in silico*, and site-directed mutagenesis studies (A and B) Concentration-dependent biophysical evaluation of Z0933M interaction with Skp1^WT^ and ΔSkp1^1−140^ using TSA assay, respectively. (C) Comparative analysis for Z0933M and 6-OAP ability to induce thermal stabilization for Skp1^WT^ and ΔSkp1^1−140^ proteins in TSA assay. (D and E) Concentration-dependent biophysical evaluation of Z0933M interaction with Skp1^WT^ and ΔSkp1^1−140^ using SPRi assay, respectively. (F) Biophysical evaluation of 6-OAP interaction with Skp1^WT^ and ΔSkp1^1−140^ using SPRi assay. (G) *In silico* prediction of Z0933M interaction with the P1 hydrophobic hotspot by IFD. (H and I) Respective RMSD and RMSF graphs for Skp1 apo and Skp1 + Z0933M trajectories resulting from 200 ns MD simulations. (J) The contribution of individual residues to binding predicted by energy decomposition calculations using the MM-PBSA method. (K) K_D_ values listed after SPRi evaluation of Z0933M with Skp1^WT^, and Skp1 mutant(s) forms. (L) Bar graph representation for the results obtained from LC-MS/MS analysis of the *in vitro* binding between Skp1 and Z0933M.

To validate the biochemical activity of Z0933M, we detected direct binding of the compound to Skp1 with biophysical techniques. First, in a thermal shift assay (TSA), Z0933M increased the melting temperature (Tm) of recombinantly purified Skp1, both intact (Skp1^WT^) and truncate (ΔSkp1^1−140^) forms, in a dose-dependent fashion (Figures 2A and 2B, respectively). The previously reported Skp1 inhibitor, 6-OAP (Liu et al., 2015), could not induce a considerable ΔTm shift in case of ΔSkp1^1−140^, whereas the extent of thermal stabilization it induced for Skp1^WT^ was comparatively lesser than that of Z0933M at the same concentration (Figure 2C). By using surface plasmon resonance imaging (SPRi), we measured affinity and found that Z0933M bound potently to Skp1^WT^ and ΔSkp1^1−140^ (Figures 2D and 2E), with dissociation constant (K_D_) values 54.7 ± 6.68 and 40.4 ± 8.2 nM, respectively. Again, 6-OAP could not show binding with ΔSkp1^1−140^ in the SPRi evaluation (Figure 2F). Together these results, in addition to validating 6-OAP binding at the H8-helix region, suggested the direct engagement of Z0933M at the P1 region located alongside the C-terminal extension of Skp1.

The above interpretation was further reinforced by *in silico* molecular binding simulations and the site-directed mutagenesis studies for the *in-silico* predicted Z0933M-interacting residues. By using the Schrödinger-induced fit docking (IFD) protocol, we first depicted that Z0933M appears to be spatially well aligned with the P1 architecture, mainly via face-to-face (or edge-to-face) dual stacking interactions with the aromatic cage F101 and F139 (Figure 2G). The compound binding is further stabilized via polar and hydrophobic interactions contributed by adjacent residues Q97, V123, and I141, respectively. Besides, a round of molecular dynamics (MD) simulations at a scale of 200 ns revealed the stability of dually-stacked Z0933M binding in the P1 region of Skp1 (Video S1). The root men square deviation (RMSD) and the root men square fluctuation (RMSF) graphs of C-α atoms indicated that compound binding renders an overall structural stability to the protein as compared to the apo form (Figures 2H and 2I). An overlap of structural snapshots extracted at different time points of MD trajectory over the initially docked complex displayed a structural drift in the H7-helix region, which is also indicated by an increase in the distance between C-α atoms of V123 vs F139 and V123 vs I141 residues (Figures S5A and S5B). This structural drift renders the P1 hotspot to act as a shallow pocket, aiding the insertion of compound’s aniline moiety deep inside as well as anchor strongly to the so-called aromatic cage via dual stacking interactions. Moreover, the interacting residues determined by energy decomposition of the MD trajectory were the same as predicted by IFD simulations (Figure 2J). Next, we mutated the Z0933M-interacting residues to alanine and/or the polar/charged substituents and then performed the SPRi evaluation. As listed in Figure 2K, a remarkable decrease in the binding affinity of Z0933M was observed with mutant forms of Skp1. The binding affinity in case of Skp1^F139D^, Skp1^V123D^, and Skp1^F101A−F139A^ mutants was particularly decreased to a magnitude of several folds. This probably relates to the loss of dual stacking interactions and/or a change in the hydrophobic character of P1 region, respectively. Adding to this, we also performed an additional *in vitro* binding assay using liquid chromatography-tandem mass spectrometry (LC-MS/MS) (see Figure S6 for the details). The extracted information from the comparative analysis of LC-MS/MS spectra and the SDS-PAGE band intensities revealed that whereas Z0933M showed robust binding with Skp1^WT^, ΔSkp1^1−140^, and Skp1-ΔF-box complex, its ability to bind with Skp1^F139A^ mutant protein was markedly reduced (Figure 2L). Interestingly, Z0933M was absent in the mass spectra of recombinant GST sample that we used as a negative control. This excluded the possibility of non-specific binding by Z0933M. The mutations of other interacting residues did not show a considerable difference in binding (data not shown). The markedly reduced binding of Z0933M with Skp1^F139A^ mutant consolidated not only our initial hypothesis but also the results from the *in silico* predictions and the SPRi evaluation where F139 was interpreted to be one of the key interacting residues. Based on these results, one may conclude that Z0933M may physically bind at P1 hydrophobic hotspot region in the F-box binding interface of Skp1.

Video S1. MD simulations at a scale of 200 ns revealing dually-stacked Z0933M binding in the P1 region of Skp1, related to Figure 2

### Z0933M disrupts Skp1-F-box PPIs *in cellulo*, impairs SCF E3 ligase functioning, and alters the turnover of substrate proteins

**Figure 3:**
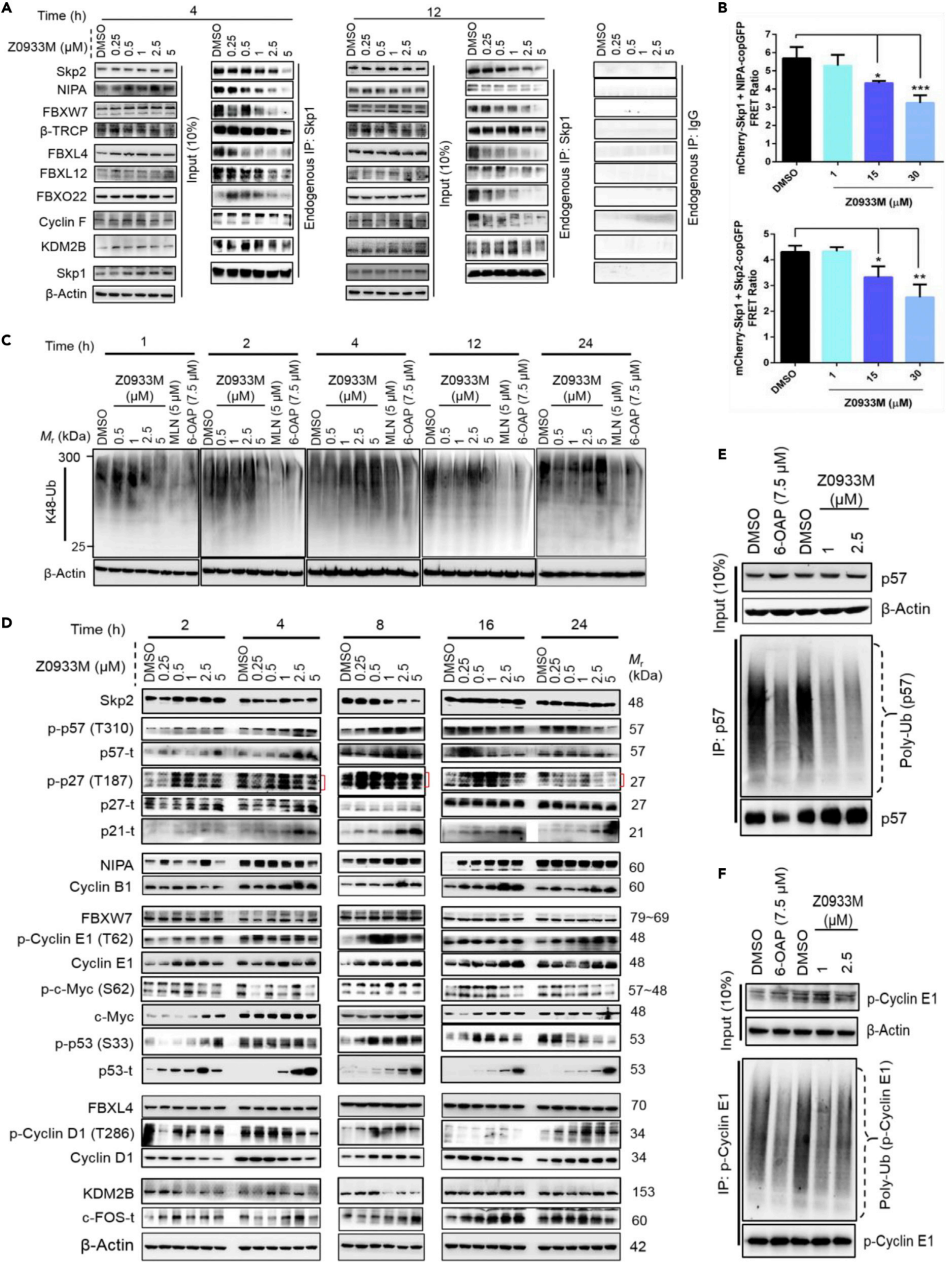
*In cellulo* assessment of Z0933M ability to disrupt Skp1-F-box PPIs and alter SCF E3 ligase(s) activity (A) Endogenous co-IP assay involving Skp1 pull-down after dose- and time-dependent Z0933M treatment of A549 cells. (B) Cell-based FRET assay in HEK293 cells, demonstrating Z0933M ability to disrupt Skp1-F-box PPIs in intact cells. Data represent mean ± SD. ∗*p* < 0.05, ∗∗*p* < 0.01, ∗∗∗*p* < 0.001, and (*n* = 3), one-way ANOVA test. (C) K48-linked cellular ubiquitination levels determined after dose- and time-dependent Z0933M treatment of A549 cells. (D) Western blot analysis for the cellular levels of respective substrate proteins recognized by SCF^Skp2^, SCF^NIPA^, SCF^FBXW7^, SCF^FBXL4^, and SCF^KDM2B^. (E and F) Effect of 6-OAP and Z0933M treatments on the ubiquitination of individual substrate proteins recognized by SCF^Skp2^ (p57) and SCF^FBXW7^ (p-Cyclin E1), respectively.

As described earlier, P1 hotspot encompasses the F-box-biding interface of Skp1 and is critically involved in mediating Skp1 interactions with several F-box proteins (Figures S1 and S2). Therefore, we next sought to characterize the antagonistic effect of Z0933M on Skp1-F-box PPIs in a cellular system. First, an endogenous co-IP assay involving Skp1 pull-down was performed (Figure 3A). Based on previous experience (Liu et al., 2015), the xenograft-amenable A549 lung cancer cell line was selected for these experiments. The Western blot analysis of samples prepared after Z0933M treatment exhibited a dose- and time-dependent dissociation of Skp1 from its binding partners (Figure 3A). A clearer Z0933M inhibitory effect could be observed against Skp2, NIPA, FBXW7, and FBXO22, the F-box proteins for which the interaction with Skp1 was also strongly reduced after inducing P1 mutations in the earlier preliminary investigations (Figure S2). In the case of β-TRCP, cyclin F, and KDM2B, nevertheless, the effect was not prominent at 4 h, although the compound was able to disrupt the interaction at 2.5–5 μM dose points after 12 h. This result hinted at Z0933M ability to possibly modulate Skp1-F-box PPIs *in cellulo*, pertaining to interaction plasticity manifested by different F-box proteins with the H8-helix and the P1 hotspot of Skp1. We also conducted an additional Flag-IP experiment with Skp1 WT and mutant(s) plasmids to validate the results from endogenous pull-down assay (Figure S7). In this case, whereas A549 cells were transiently transfected with Flag-*Skp1* WT and mutant plasmids, the effect of Z0933M treatment was monitored on Skp1-F-box PPIs (of cyclin F and β-TRCP) that still retained their binding capacity in our initial evaluation of P1 site-directed mutants. The resulting data revealed that although Z0933M prevented the interaction of Skp1^WT^ with indicated F-box proteins, its ability was abolished or diminished to do so in case of Skp1^F101A^ and Skp1^F139A^ (Figure S7). FBXO22 could not be pulled-down with mutant(s) forms except Skp1^Q97A^, which is consistent with our preliminary investigations (Figure S2). To further verify that Z0933M disrupts Skp1-F-box PPIs in intact cells, we subjected it to the cell-based FRET assay that initially was employed for examining P1 mutants (Figure S2). Z0933M treatment showed significant inhibition of Skp1 interaction with NIPA and Skp2, as indicated by dose-dependent reduction in FRET ratio (Figure 3B). Taken together, these results suggested that Z0933M may disrupt and/or modulate Skp1-F-box PPIs *in cellulo*.

Encouraged by the above results, we next evaluated the effect of Z0933M treatment on the global levels of K48-linked polyubiquitination, a molecular feature associated with SCF E3 ligase(s)-mediated proteolysis of several cellular proteins. Side-by-side controls in this experiment included the neddylation inhibitor MLN4924 (Soucy et al., 2009), and 6-OAP. As shown in Figure 3C, the magnitude of treatment effects varied dose- and time-dependently for the three compounds. This, probably, could be explained by the highly buffered, diversified, and “just in time” activity-based dynamics of the human cellular SCF repertoire, aiding the cell to adapt to the changing pool of substrates (Hill et al., 2019; Reitsma et al., 2017; Wang et al., 2020). Overall, Z0933M treatment modulated cellular K48-ubiquitination levels with fairly similar trends as were observed with 6-OAP (Figure 3B). At the earliest time points examined (1 and 2 h), Z0933M-induced decrease in K48-polyubiquitin chains was clearly evident at 5 μM dose. The reduction in K48-linked ubiquitination at 0.5–1 μM dose points of Z0933M was similar in extent to that observed by 7.5 μM of 6-OAP after 24 h treatments (Figure 3A). In line with these observations, Western blot analysis of the Z0933M-treated A549 cellular lysates, revealed dose- and time-dependent accumulation of various substrate proteins (Figure 3D), an outcome consistent with the concept that loss of K48-polyubiquitylated chains impairs the targeting of proteins for proteasomal degradation. In particular, the increased levels of several phosphorylated (p) proteins hinted at the Z0933M-derived decommissioning of the corresponding SCF E3 ligase(s) activity, since SCF assembly recognizes substrate proteins only when they are phosphorylated (Jin et al., 2005; Kitagawa et al., 2009; Lin et al., 2006; Tang et al., 2005; Zheng et al., 2014). Again, the magnitude of effects for Z09333M treatment varied dose- and time-dependently, which also could be rendered to the activity-based dynamics of the human cellular SCF repertoire as mentioned above. This interpretation could further be endorsed by modulated protein levels of some F-box proteins, such as Skp2 and NIPA, in a dose- and time-dependent fashion after Z0933M treatment. In addition, Z0933M caused a reduction in the ubiquitination of p57 (Figure 3E) and (*p*-Cyclin E1) (Figure 3F), the individual substrate proteins recognized by SCF^Skp2^ and SCF^FBXW7^, respectively. The effect of Z0933M treatment at 1 μM was comparable to 7.5 μM of 6-OAP. Moreover, the effect of Z0933M treatment on substrate accumulation could even be observed at lower dose points (0.25–1 μM), a result that is comparable to the half-maximal effective concentration (EC_50_) value(s) demonstrated in certain cancer cell lines including A549 cells (see below). It is also worth mentioning that increases in protein levels of certain substrates observed with the lower Z0933M concentration were generally not seen for the higher concentration (5 μM), probably owing to the higher rate of apoptosis observed at 14 and 24 h of treatment (described below).

Collectively, the above-described findings suggested that Z0933M may inhibit and/or modulate the functional activity of several SCF E3 ligases and, in turn, increase the levels or alter the turnover of several substrate proteins.

### Z0933M recapitulated the previously reported effects of Skp1-reduced activity, whereas Skp1 overexpression reduced Z0933M sensitivity in cancer cells

One expects that a potent chemical inhibitor will recapitulate the knockdown effects of its target protein, whereas overexpressing the target protein will render the cells less sensitive to the growth inhibitory effects of the compound. Thus, evaluating the effect of Z0933M treatment in context of previously reported effects of Skp1-reduced activity or by increasing the cellular level of Skp1 may constitute a simple test to confirm if Skp1 is indeed the physiologically relevant target of Z0933M.

**Figure 4:**
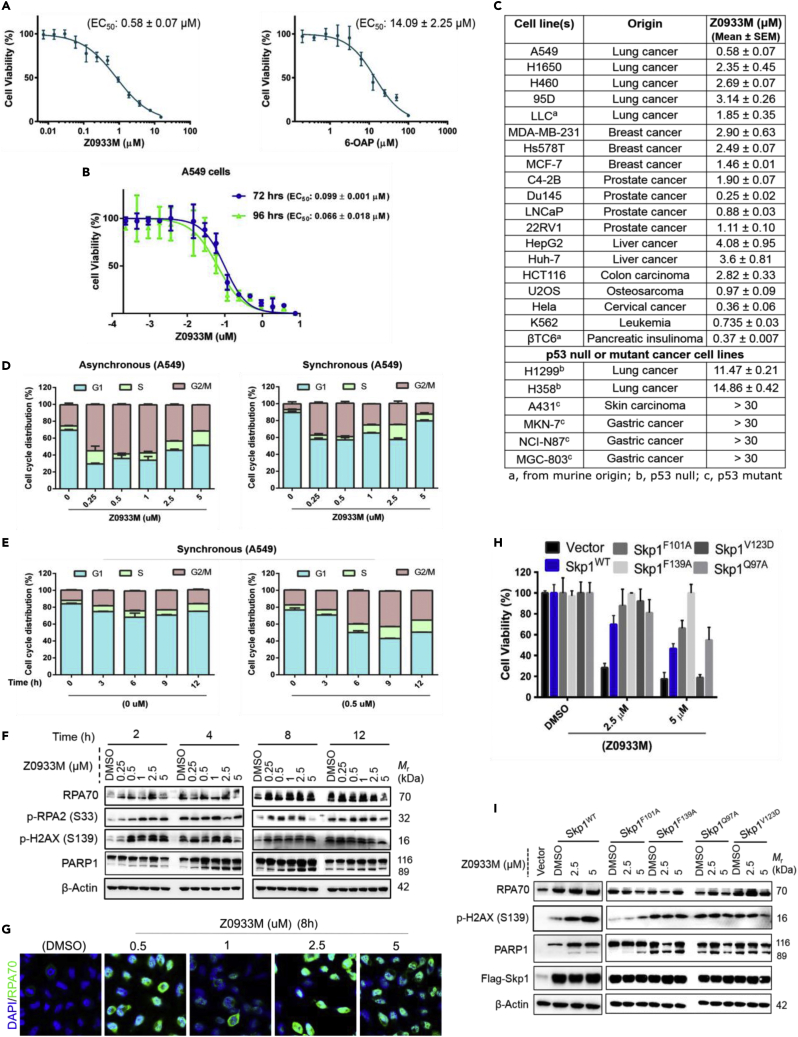
Assessment of Z0933M efficacy in context of the effects reported previously for Skp1 reduced activity, and overexpression (A) Dose-response graphs for the cell viability experiment of A549 cells at 48 h comparing the cellular potency of Z0933M and 6-OAP. (B) Dose-response graphs for the cell viability experiment of A549 cells showing cellular potency of Z0933M at 72 and 96 h. (C) Anti-proliferative EC_50_ values of Z0933M listed after cell viability experiments against a variety of cancer lines. (D) Flow cytometric analysis of cell cycle profiles after dose-dependent Z0933M treatment of A549 cells for 24 h. (E) Flow cytometric analysis of cell cycle profiles after time-dependent Z0933M treatment of A549 cells at 0.5 μM. (F) Western blot analysis showing the effect of Z0933M treatment on the cellular levels of proteins associated with reduced activity of Skp1. (G) Confocal microscopy result for RPA70 after dose-dependent treatment of A549 cells. (H) Effect of Z0933M treatment on the cell viability of A549 cells transfected with Skp1 WT and mutant(s) plasmids. (I) Western blot analysis of the proteins previously implicated in Skp1-reduced activity from the cellular lysates generated after Z0933M treatment of A549 cells transfected with Skp1 WT and mutant(s) plasmids.

Knockdown experiments have previously suggested that survival of some cancer cell lines (including A549) may depend on Skp1 (Liu et al., 2015; Tian et al., 2020; Zhu et al., 2021), whereas its pharmacological inhibition with 6-OAP has been demonstrated to reduce the viability of A549 cells (Liu et al., 2015). We therefore tested the effect of Z0933M treatment on the viability of A549 cells. As our main goal was to identify a cellularly potent compound, we included 6-OAP as a control for comparison of potency. Intriguingly, the anti-proliferative activity of Z0933M against A549 cells was demonstrated to be approximately 24 times more potent than 6-OAP (EC_50_ values: 0.58 ± 0.07 μM versus 14.09 ± 2.25 μM, respectively) in our hands (Figure 4A). Moreover, the EC_50_ further dropped to double-digit nanomolar range when A549 cells were treated for 72 (EC_50_ = 0.099 ± 0.001 μM) and 96 h (EC_50_ = 0.066 ± 0.018 μM) (Figure 4B). Encouraged by this, we further tested the compound in a panel of cell lines derived from hematologic and solid tumors. As depicted in Figure 4C, Z0933M treatment induced complete cell killing of all the lines tested, with variable EC_50_ values ranging from 0.25 to 4 μM. This highlights the potential of Z0933M to serve as an anticancer agent. Surprisingly, Z0933M demonstrated substantially weaker or absolutely no anti-proliferative effects against p53-deficient (null and mutant) cell lines, hinting that probably the mechanism of cell killing by this compound solely depends upon p53 (described in next section).

As *Skp1* knockdown and/or Skp1 pharmacological inactivation are also known to cause G2/M phase cell-cycle arrest (Liu et al., 2015), we explored the effect of Z0933M on cell cycle kinetics by flow cytometry. Consistent with the preceding statement, Z0933M treatment induced a complete G2/M arrest at concentrations near its EC_50_ value (0.5 μM) in both asynchronized and synchronized A549 cells (Figures 4D and 4E). However, at higher concentrations (≥1 μM), Z0933M treatment resulted in arrests at G1, S, and G2/M phases of the cell cycle, which was suggestive of the defective ubiquitination or proteolysis of the proteins that are critical for proper passage through the cell cycle. Protein levels of the cell cycle markers cyclin B1 and cyclin D1, and of the cyclin-dependent kinase (CDK) inhibitors p21 and p27 can be correlated with the cell cycle profiles observed in Z0933M-treated cells.

Recent knockdown studies have suggested that *Skp1* is critical for coordinated preservation of genomic stability (Lepage et al., 2021; Piva et al., 2002; Thompson et al., 2020). Z0933M, as an Skp1 inhibitor, would therefore be expected to adversely impact the DNA repair pathways through aberrant protein turnover of the key substrates involved in those processes. For example, Z0933M treatment led to accumulation of cyclin E1 and c-Myc (Figure 3D), both of which are known to mediate genomic instability and replication stress in response to reduced Skp1 activity (Lepage et al., 2021; Piva et al., 2002; Thompson et al., 2020). Similarly, Z0933M treatment corresponded with dose- and time-dependent increases in the levels of RPA (Replication Protein A) and γ-H2AX (S139) (Figure 4F), the surrogate markers of replication stress and DSB, respectively, which recently have been implicated in dysregulation of DNA replication and repair manifested by *Skp1* knockdown (Thompson et al., 2020). We also carried confocal microscopy that showed increased levels of RPA70 at all dose points in Z0933M-treated A549 cells as compared to the untreated control (Figure 4G). In line with these observations, we found a Z0933M dose- and time-dependent increase in cleaved PARP1 (89 kDa) (Figure 4F), an indicator of severe DNA-damage-induced apoptosis and necrosis (Bouchard et al., 2003; Qvarnstrom et al., 2009), which also suggested the impairment of DNA repair processes. These observations collectively hinted at a potentially similar impact of Z0933M on genome stability as observed previously for Skp1-reduced activity (Lepage et al., 2021; Piva et al., 2002; Thompson et al., 2020).

If Skp1 is indeed the target of Z0933M, then overexpression of the target should render the cells less sensitive to the compound. Therefore, we examined the effect of Z0933M treatment on cell viability of A549 cells that were transiently transfected with Flag-*Skp1* WT and mutant(s) plasmids. The results showed that whereas Z0933M displayed a potent inhibitory effect on the viability of control (vector-transfected) cells in a dose-dependent manner, its killing effect was compromised or nearly completely rescued upon transfection with Flag-*Skp1* WT and mutant(s) plasmids (Figure 4H). Similarly, at molecular level, transfections with Flag-*Skp1* mutant(s), particularly Flag-Skp1^F^^139A^, compromised Z0933M ability to induce accumulation of RPA70, γ-H2AX (S139), and cleaved PARP1 (Figure 4I).

Altogether, the aforementioned data clearly suggested that the actions of Z0933M are indeed mediated through Skp1 inhibition inside the cells.

### Z0933M elicits apoptotic cell death which is reversed by p53 inhibition and Skp1 overexpression

**Figure 5:**
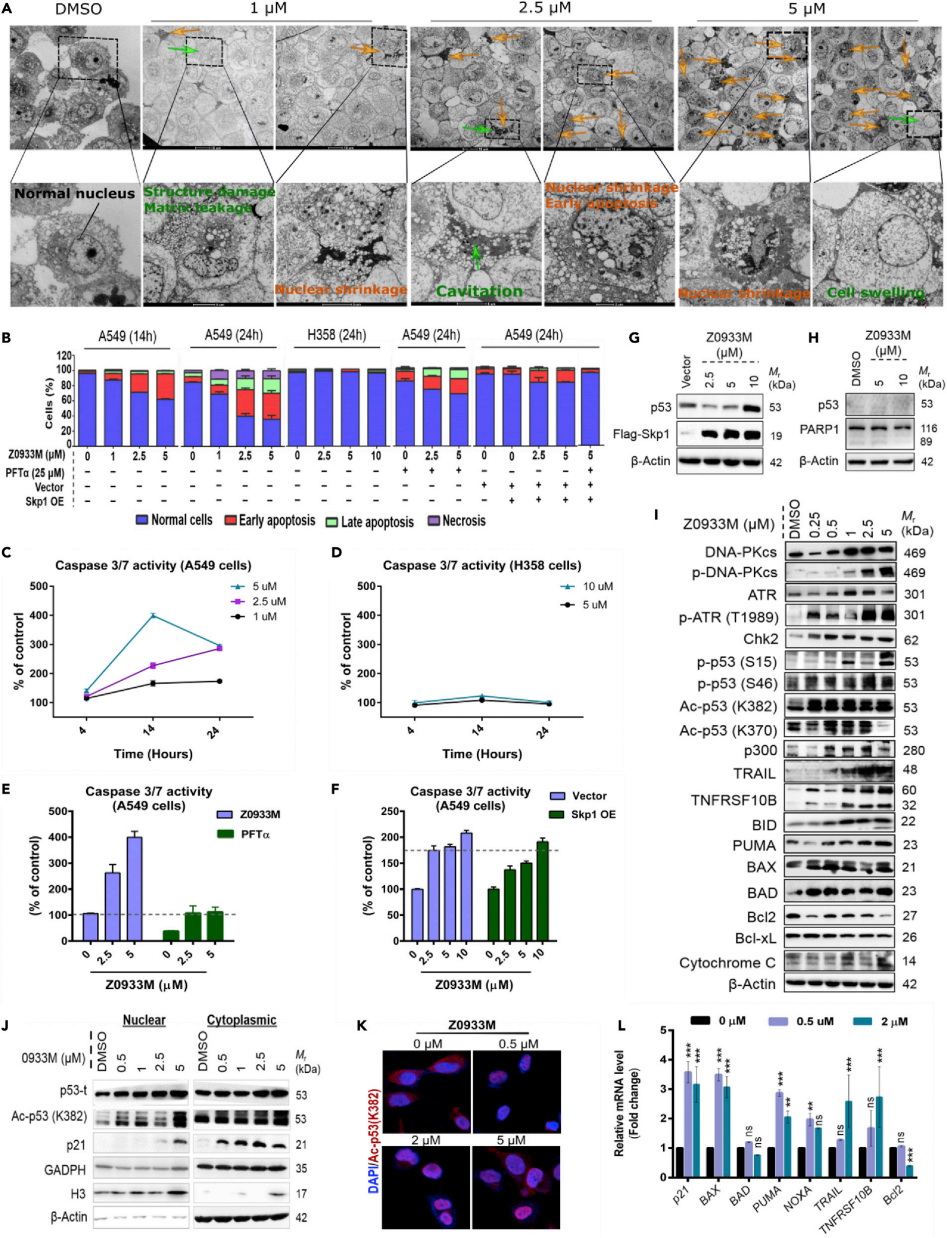
Mechanistic insights into p53-dependent cell death response elicited by Z0933M (A) TEM images for untreated and Z0933M-treated A549 cells. Orange arrows refer to apoptotic cells, whereas green arrows indicate necrosis. The scale bar for upper panels is 10 μm, and for lower panels 2 μm. (B) Flow cytometric analysis for apoptosis and necrosis. (C and D) Dose- and time-dependent effect of Z0933M treatment on the caspase 3/7 activity in A549 and H358 cells, respectively. (E) Dose-dependent effect of Z0933M treatment (14 h) on the caspase 3/7 activity in A549 cells pretreated with p53-inhibitor PFTα. (F) Dose-dependent effect of Z0933M treatment (14 h) on the caspase 3/7 activity in A549 cells transfected with Flag-*Skp1*. (G) Western blot analysis of p53 levels in the cellular lysates of Skp1-overexpressing and Z0933M dose-dependently (14 h) treated A549 cells. (H) Western blot analysis of PARP levels in the cellular lysates of Z0933M dose-dependently (24 h) treated H358 cells. (I) Western blot analysis of the proteins indicating DNA-damage-induced p53 activation, as well as increased levels of the proteins associated with intrinsic (mitochondrial)- and extrinsic (TRAIL)- apoptotic pathway(s) after dose-dependent Z0933M treatment (14 h) of A549 cells. (J) Western blot analysis for p53 and its direct transcriptional target p21 in the nuclear and cytoplasmic fractions of A549 cellular lysates after Z0933M treatment (14 h). (K) Confocal microscopy images indicating that Z0933M treatment (14 h) enhances Ac-p53 (K382) translocation to the nucleus. (L) RT-qPCR analyses for direct (*p21*, *BAX*, *PUMA*, *TRAIL*, and *TNFRS10B*) and indirect (*Bcl2*) transcriptional targets of p53 after Z0933M treatment (14 hrs). mRNA expression was normalized to *β-Actin*. Data represent mean ± SD. ∗p < 0.05, ∗∗p < 0.01, ∗∗∗p < 0.001, and (*n* = 3), one-way ANOVA test.

We next sought to gain insights about the cellular and molecular mechanisms for Z0933M killing of cancer cells. Z0933M treatment of A549 cells induced upregulation of γ-H2AX (S139) and cleaved PARP1 (Figure 4F). It, therefore, would be expected to induce apoptotic/necrotic cell death (Bouchard et al., 2003). To confirm this, we first performed transmission electron microscopy (TEM) that displayed classical morphological features indicative of apoptosis and necrosis (Figure 5A). To more directly characterize the apoptotic/necrotic cell death, we performed flow cytometry analysis of Z0933M-treated A549 cells by annexin V/propidium iodide (PI) double staining. Annexin V represents the early marker of apoptosis, whereas the PI is the dead cell marker. Z0933M treatment of A549 cells increased the percentages of cells in quadrants representing necrosis (Annexin V^−^/PI^+^), late apoptosis (Annexin V^+^/PI^+^), and early-apoptosis (Annexin V^+^/PI^−^) in a dose- and time-dependent manner (Figures 5B and S8A). The early apoptotic response was robust and was significantly induced after 14 and 24 h of treatment. The late apoptotic and necrotic cell populations were markedly induced at 24 h.

As mentioned above, Z0933M treatment demonstrated substantially weaker or no sensitivity toward p53-deficient or –null cell lines (Figure 4C), whereas it markedly increased stability of p53 in A549 cells (Figure 3D). This prompted us to reason that whether Z0933M-induced apoptosis is mediated by p53 activation. Indeed, p53-dpendent apoptotic cell death response has recently been reported for the neddylation inhibitor, MLN4924, which acts upstream of the SCF assembly (Ferris et al., 2020). Therefore, to answer the above-mentioned statement, we performed a series of experiments at cellular and molecular level. First, we conducted the flow cytometric analysis of Z0933M-treated H358 (p53 null) cells, which indicated that, if anything, H358 cells were more resistant to Z0933M-induced apoptosis than A549 cells (Figures 5B and S8B). Z0933M could not induce apoptosis in H358 cells even at 10 μM dose. Similar results were also observed at a molecular level, where Z0933M treatment dose- and time-dependently induced caspase 3/7 activity, an apoptotic cell death marker, in A549 cells (Figure 5C) but it failed to do so in H358 cells (Figure 5D). Moreover, pharmacological inhibition of p53 with Pifitherin-α (PFTα) revealed the necessity of p53 in Z0933M-induced apoptosis and caspase 3/7 activity in A549 cells (Figures 5B and 5E, respectively, and Figure S8C). Interestingly, transfecting A549 cells with Flag-*Skp1* markedly reduced the proportions of apoptotic/necrotic cells (Figures 5B and S8C), as well as compromised the induction of caspase 3/7 activity (Figure 5F), after Z0933M treatment at lethal dose points (2.5–5 μM). Moreover, pretreatment of Flag-Skp1-transfected A549 cells with PFTα almost completely abolished the ability of Z0933M to induce apoptosis/necrosis (Figures 5B and S8C). To determine if increased Skp1 levels inversely relate with Z0933M-induced p53 stability, we performed Western blot analysis for p53 from the cellular lysates of Flag-*Skp1* transfected A549 cells that initially were prepared for flow cytometric analysis. As shown in Figure 5G, Skp1 overexpression has abrogated the Z0933M ability to induce p53 accumulation at 2.5- and 5-μM dose points, although compound treatment was still able to induce p53 stability at 10 μM. Apart from this, Western blot analysis of Z0933M-treated H358 cellular lysates did not show PARP cleavage (Figure 5H), probably owing to the lack of p53 activity. Together, these findings indicated that Z0933M elicits apoptotic/necrotic cell death by p53-dependent mechanism.

In line with the above findings, the Western blot analysis for whole cell extracts revealed dose-dependent accumulation of DNA-PKcs, p-DNA-PKcs (S2056), ATR, p-ATR (T1989), p-p53 (S15), Chk2, p-p53 (S46), Ac-p53 K370, Ac-p53 K382, and p300 (Figure 5I). S15 and S46 lie amongst the concurrent p53 phosphorylations – mediated by DNA-PKcs and ATR in response to DNA damage and checkpoint activation (Reed and Quelle, 2014) – that facilitate efficient p300-p53 association and p300-mediated acetylation (K370 and K382), and also promote p300-p53 co-recruitment onto the promoter regions of transcriptional targets in DNA (Reed and Quelle, 2014). Consistent with this, confocal microscopy for Z0933M-treated and Z0933M-untreated A549 cells revealed enhanced nuclear localization of acetylated(Ac)-p53 K382 (Figure 5J), a result also confirmed by Western blot analysis of nuclear and cytoplasmic fractions of Z0933M-treated A549 cellular lysates (Figure 5K). Moreover, RT-qPCR analysis for Z0933M-treated A549 cellular extracts revealed significantly enhanced transactivation ability of p53 toward its transcriptional targets that are associated with extrinsic and intrinsic (mitochondrial) pathways of apoptosis signaling (Figure 5L). Direct p53-target genes were upregulated (*p21*, *BAX*, *PUMA*, *TRAIL*, and *TNFRS10B*), whereas indirect target gene (*Bcl2*) was downregulated. The Western blot analysis also highlighted the dose-dependent up- and down-regulation of these proteins (Figure 5I). Collectively, these data suggested that Z0933M treatment induces p53-mediated apoptotic cell death response.

**Figure 6:**
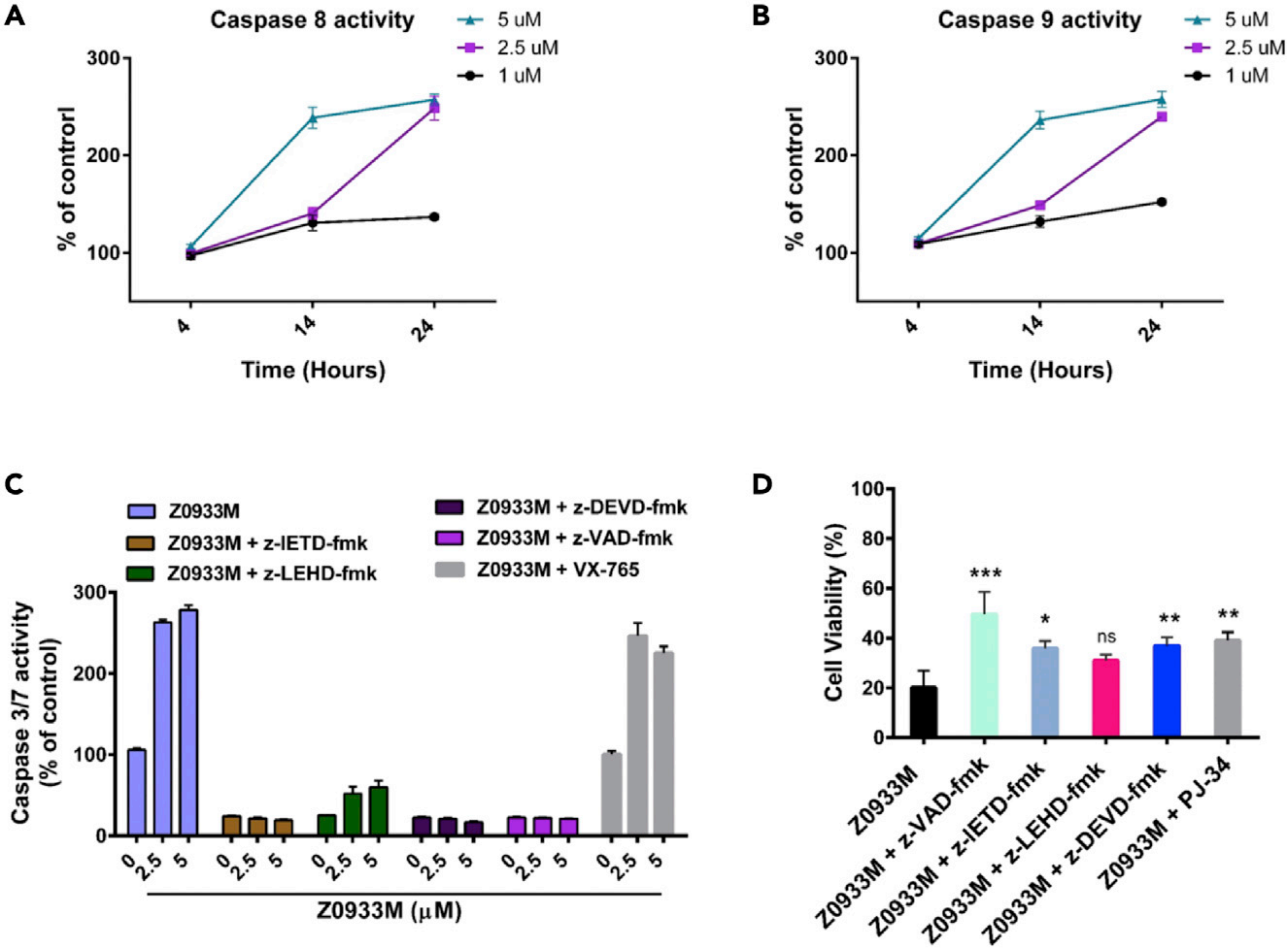
Z0933M may activate intrinsic and extrinsic pathways of apoptosis (A and B) Dose- and time-dependent effect of Z0933M treatment on the caspases-8 and -9 activities in A549 cells, respectively. (C) Effect of inhibitor interventions on caspase 3/7 activity after Z0933M dose-dependent treatment (14 h) of A549 cells. (D) Effect of inhibitor interventions on the cell viability of Z0933M-treated A549 cells. Data represent mean ± SD. ∗p < 0.05, ∗∗p < 0.01, ∗∗∗p < 0.001, and (*n* = 3), one-way ANOVA test.

The above data showed that Z0933M treatment increases the expressions of proteins serving as upstream regulators of extrinsic (TRAIL, TNFRSF10B) and intrinsic (PUMA, BAX, BAD) apoptotic pathways. The involvement of intrinsic (mitochondrial) apoptosis in mediating a cell death response to Z0933M was also confirmed by dose-dependent low expression of antiapopotic Bcl-2 and Bcl-xL molecules, and an increase in the level of pro-apoptotic protein BID and the cytochrome *c* release (Figure 5B). Indeed, the existing literature highlight that caspase-8 serves as the key upstream mediator in TRAIL-mediated and p53-derived mitochondrial apoptosis via BID (Lees et al., 2020; Seo et al., 2017). Subsequent investigations revealed that Z0933M treatment of A549 cells induced the cleavage activities of caspase-8 and casapse-9, the two representative hallmarks of extrinsic and intrinsic apoptotic pathways, respectively (Figures 6A and 6B). Pharmacological inhibition of caspase-8 (with z-IETD-fmk) almost completely reversed the caspases 3/7 activity, with similar magnitude of effect as was observed with individual treatments of caspase-3/7 (z-DEVD-fmk) and pan-caspase (z-VAD-fmk) inhibitors (Figure 6C). Likewise, caspase-9 inhibitor (z-LEHD-fmk) also impaired the Z0933M ability to induce caspases 3/7 activity. However, caspase-1 inhibitor (VX-765) failed to abolish Z0933M ability of activating caspases 3/7, indicating that apoptotic response is mainly derived by caspases-8 and -9. In addition, the individual caspase(s) inhibitors, as well as the PARP1 inhibitor (PJ-34), also showed an improvement in viability for A549 cells after treatment with lethal dose (5 μM) of Z0933M (Figure 6D). However, the effect of caspase 9 inhibitor (z-LEHD-fmk), although it compromised the Z0933M ability of killing, was not found to be significant (Figure 6D). These results together suggested that Z0933M-induced apoptotic cell death is predominantly mediated by extrinsic apoptotic pathway.

## Discussion

Cellularly active and potent small-molecule Skp1 inhibitors as a chemical tool to explicate Skp1 biology are of utmost interest. We have reported here the identification of Z0933M as a cellularly potent chemical inhibitor of the Skp1-F-box PPIs. Using structure-guided site-directed mutagenesis studies, we have demonstrated that Z0933M engages with a hydrophobic P1 hotspot region situated at the F-box binding interface of Skp1 that normally accommodate aromatic/hydrophobic side-chains from different F-box proteins, thereby directly competing with Skp1:F-box PPIs. Consistent with this fact, we have shown that Z0933M disrupted the SCF E3 ligase(s) functioning and, in turn, altered the protein turnover of several substrate proteins. Moreover, we have presented different lines of evidence suggesting that Z0933M recapitulated previously reported effects of *Skp1* knockdown and/or Skp1 pharmacological inhibition, such as tumor cell growth inhibition, cell-cycle arrest, and accumulation of proteins associated with replication stress. Apart from this, Z0933M may potentially induce death response attributed to pharmacological decommissioning of SCF E3 ligases (Chen et al., 2016; Luo et al., 2012), although previously not demonstrated for 6-OAP. Our findings have several important implications.

Z0933M can be used as a tool molecule for interrogating Skp1 biology and prospective therapeutic avenues in cancer settings. Z0933M is the structural analogue of Z0933, an anti-tuberculous compound reported recently by a joint effort of our group (Makafe et al., 2019). However, unlike Z0933 (Makafe et al., 2019), Z0933M demonstrated negligible potency (MIC >30 μM) in anti-mycobacterial evaluation. This addresses the potential application of this experimental compound as a chemical tool for explicating Skp1 biology. To this context, another structural analog, g-203 (Makafe et al., 2019); which showed absolutely no or substantially weaker activity in our *in vitro* biochemical and cell viability experiments (Figure S9), may potentially be accompanied as a negative control ligand. Apart from this, both Z0933M and JH-3 may serve as a chemically tractable starting point(s) for the further SAR studies in order to identify optimized inhibitory compounds with the potential for development into a drug compound suitable for pre-clinical and clinical studies. On a functional note, the modulation of K48-linked global ubiquitination levels and of the several substrate proteins by Z0933M further strengthens the possibility that perturbed Skp1 activity in the molecular machinery may alter the cellular homeostasis of several proteins. Considering the dynamic nature and plasticity of Skp1-F-box PPIs, the small-molecule targeting of Skp1 may potentially have different consequences in case of different SCF E3 ligases. Future studies ought to be endorsed for probing this fact, particularly taking into consideration the “just in time” activity-based dynamics of the human cellular SCF repertoire.

The discovery of small molecule inhibitors of PPIs is generally considered to be a daunting task, mostly owing to the flat and large interacting protein interfaces comprising shallow hotspot regions (Arkin et al., 2014). And, the design of ligands which specifically could address a well-defined hotspot on the protein interface remains challenging (Arkin et al., 2014; Kubota and Hamachi, 2015). Considering the case of Skp1’s F-box-binding interface, despite the interest, only one moderately potent small-molecule inhibitor, 6-OAP, has been reported so far (Liu et al., 2015). However, from a chemical perspective, 6-OAP possesses a relatively hard-to-modify core structure. In addition, the binding site of 6-OAP on the F-box-binding interface of Skp1 (H8 helix) has been reported to be structurally unstable region in free form of the protein (Chandra Dantu et al., 2016). These facts have probably impeded the exploration of 6-OAP to find more potent analogs through SAR studies. In this study, we attempted a structure-guided hypothesis-driven approach to find a new inhibitor chemotype(s) against the F-box-binding interface of Skp1. The differentiating factor of this approach relies on the fact that it explicitly considers the hydrophobic character of a hotspot region alongside the C-terminal extension of Skp1. The identification of Z0933M as a potent Skp1 inhibitor through this approach reveals what is likely to be generally workable strategy to discover or optimize next-generation Skp1 inhibitors. Although the compound dataset employed was small, the current work clearly demonstrated the feasibility of our approach, i.e. the ligands with bi- or multi-cyclic heteroaromatic architecture would show strong binding with the phenylalanine aromatic cage system.

From a mechanistic perspective, we identified p53 activation as the key molecular feature predicting sensitivity to apoptotic response by Z0933M-mediated Skp1 inhibition. p53 is a *bona fide* substrate of SCF assembly (Cui et al., 2020). We found that Z0933M caused upregulation of both total and phosphorylation levels of p53, as well as its transcriptional activity in A549 cells, likely in part through the induction of DNA damage response. Previous studies have demonstrated that MLN4924, the neddylation inhibitor (Soucy et al., 2009), also activates p53 by modifications including phosphorylation, leading to its accumulation and increase of transcriptional activation of pro-apoptotic factors such as TRAIL, Puma, and Bax, as well as cell cycle regulation-related proteins such as p21 (Ai et al., 2018; Ferris et al., 2020; Jia et al., 2011; Lin et al., 2010). In a general perspective, our results also unveiled similar mechanisms. Whereas Z0933M may stabilize p53 via the disruption of SCF assembly, we note that p53-dependent effects of Z0933M-caused Skp1 inhibition may not be solely driven by decommissioned activity of SCF ligases. Recent studies have shown that *Skp1* knockdown also leads to increased genomic instability and DSB damage by upregulating RPA70 and γH2AX, although the mechanism was not deeply explored (Thompson et al., 2020). In our findings, Z0933M recapitulated the effects of Skp1-reduced activity as indicated by upregulated protein levels of RPA70 and γH2AX (Figure 4F). Moreover, Z0933M treatment increased the levels of DNA-PKcs, the hallmark protein of non-homologous end-joining (NHEJ) DNA repair mechanism (Jiang et al., 2015), together with ATR (Figure 5I). Both DNA-PKcs and ATR have a regulatory role in the homologous recombination (HR) and NHEJ repair mechanisms of chromosomal instability-induced DSBs, and this regulation is mediated by synergistic phosphorylations of both p53 (e.g. S46) and RPA (e.g. RPA32) (Serrano et al., 2013). Interestingly, Z0933M treatment increased the levels of phosphorylated p53, such as p-p53 (S46 and S15) and RPA32 (including p-RPA2 (S33)). Taking these facts into account, one may infer a complex crosstalk between Skp1 inhibition-derived p53 activation and DNA damage response and repair pathways, leading to cell death. Clearly, a deep mechanistic study ought to be endorsed, which probably would shed light on Skp1’s role in DNA damage response signaling.

Our work provides the convincing support that actions of Z0933M inside the cells are indeed mediated through binding with Skp1 and the resulting disruption of Skp1-F-box PPIs. However, one potential caveat is that the compound demonstrated more potent phenotypical (anti-proliferation) function than it did in the cellular target engagement (which could be evidenced in part by the rescue (Figure 4H) and the Flag-IP experiments (Figure S7)). Considering this fact, the involvement of potentially off-target effects in Z0933M functioning cannot be overruled. As a matter of fact, we also conducted rescue experiment with lower doses (0.5 and 1 μM), where the killing effect of compound was almost completely rescued by Skp1 overexpression (data not shown). This suggested Skp1 as the main cellular target protein for Z0933M. Nevertheless, the potential involvement of off-target effects in Z0933M functioning warrants a full fledge separate study that is currently in progress.

In summary, we have described the identification Z0933M as a cellularly potent Skp1 inhibitor. Future efforts will now be endorsed to understand the interplay of Z0933M-mediated p53 regulation in relevance to Skp1 physiology in the multiple DNA repair pathways. Adding to this, many other details remain to be elucidated regarding Z0933M-derived perturbation of Skp1 partnering in the cellular machinery. Referring to existing literature evidence(s), a few questions would be worth investigating. For instance, how Z0933M treatment alters the tethered kinetics of substrate(s) degradation (Hill et al., 2019)? And, what ultimate impact would Z0933M treatment have on the cellular biogenesis in context of epigenetics, stem cell biology, and immunology (Meng et al., 2018; Wang et al., 2018; Xie et al., 2019)? Answers to these questions, and many others (Hussain et al., 2016), will reciprocally further our understanding of Skp1 cellular functioning.

### Limitations of the study

This study characterizes the identification of a Skp1-targeting compound from a limited chemical space. Moreover, the experiments for the mechanism study have been done entirely in a human cancer cell line grown in culture, which may not reflect exactly what happens in pre-clinical animal models. In addition, we acknowledge that further work is still required to rule out the potential off-target effects involved in Z0933M functioning.

## STAR★Methods

### Key resources table

**Table:** 

REAGENT or RESOURCE	SOURCE	IDENTIFIER
**Antibodies**		

Poly-Ubiquitination (FK2)	Life Sensors	Cat# AB120
K48-Ubiquitination	Abclonal	Cat# A18163; RRID:AB_2861948
Cullin 1	Proteintech	Cat# 12895-1-AP; RRID:AB_2086291
Cyclin F	Santa Cruz	Cat# sc-51520
FBXW7	Abcam	Cat# ab109617; RRID:AB_2687519
FBXL4	Santa Cruz	Cat# sc-393772
FBXL10/KDM2B	Abclonal	Cat# A16017; RRID:AB_2763453
KDM2B	Santa Cruz	Cat# sc-293279
FBXL12	Abclonal	Cat# A14589; RRID:AB_2761463
FBXO22	Proteintech	Cat# 13606-1-AP; RRID:AB_2104403
NIPA	Santa Cruz	Cat# sc-365058; RRID:AB_10847677
Skp1	Santa Cruz	Cat# sc-5281; RRID:AB_2254579
Skp2	Cell Signaling Technology	Cat# 2652t
β-TRCP	Santa Cruz	Cat# sc-166492; RRID:AB_2102598
p57	Cell Signaling Technology	Cat# 2557S
p-p57 (T310)	Cell Signaling Technology	Cat# 2558S
p21	Beyotime	Cat# AP021; RRID:AB_2904251
p27	Santa Cruz	Cat# sc-1641; RRID:AB_628074
p-p27 (T187)	ThermoFisher	Cat# 37-9700; RRID:AB_2533350
c-Myc	Beyotime	Cat# AF6513
p-c-Myc (S62)	Abcam	Cat# ab185656
p53	Abclonal	Cat# A11212; RRID:AB_2758461
p-p53 (S33)	Abbkine	Cat# abp50385
p-p53(S46)	Beyotime	Cat# AF5896
Cyclin B1	Santa Cruz	Cat# sc-166757; RRID:AB_2072277
Cyclin D1	Abclonal	Cat# A10757; RRID:AB_2758200
p-Cyclin D1 (T286)	Cell Signaling Technology	Cat# 3300T
Cyclin E1	Abclonal	Cat# A0112; RRID:AB_2756955
p-Cyclin E1 (T62)	Cell Signaling Technology	Cat# 4136S
c-Fos	Santa Cruz	Cat# sc-166940; RRID:AB_10609634
RPA70	Abcam	Cat# ab79398; RRID:AB_1603759
p-RPA2 (S33)	Abcam	Cat# ab211877; RRID:AB_2818947
p-H2AX (S139)	Beyotime	Cat# AF1201; RRID:AB_2920717
PARP1	Beyotime	Cat# AP102
PUMA	Abclonal	Cat# A3752; RRID:AB_2863135
BAX	Abclonal	Cat# A0207; RRID:AB_2757021
Cytochrome c	Abclonal	Cat# A13430; RRID:AB_2760292
BAD	Abclonal	Cat# A19595; RRID:AB_2862688
BCL2	Proteintech	Cat# 12789-1-AP; RRID:AB_2227948
p- p53-S15	Abclonal	Cat# AP0950; RRID:AB_2863860
Ac-P53-K370	Abclonal	Cat# A11012; RRID:AB_2758362
Ac-p53(K382)	Beyotime	Cat# AF2674
BID	Beyotime	Cat# AF6306
p300	Beyotime	Cat# AF6795
DNA-PKcs	Beyotime	Cat# AF1888
p-DNA-PKcs(S2056)	Abcam	Cat# ab124918; RRID:AB_11001004
ATR	Beyotime	Cat# AF6267
p-ATR (T1989)	Abcam	Cat# ab223258
Chk2	Beyotime	Cat# AF2020
Histone H3	Abclonal	Cat# A2348; RRID:AB_2631273
BcL-xL	Abclonal	Cat# A19702; RRID:AB_2862744
TRAIL	Abclonal	Cat# A2138; RRID:AB_2764157
TNFRSF10B	Proteintech	Cat# 15497-1-AP; RRID:AB_2240702
Anti-Flag (OctA)	Santa Cruz	Cat# sc-166355; RRID:AB_2017593
HRP Goat anti-Rabbit	Beyotime	Cat# A0208; RRID:AB_2892644
HRP Goat anti-Mouse	Beyotime	Cat# A0216; RRID:AB_2860575
IgG Isotype control	Abcam	Cat# ab172730; RRID:AB_2687931
Β-actin-HRP Rabbit Monoclonal antibody	Beyotime	Cat# AF5006

**Bacterial and virus strains**		

BL21(DE3) Chemically Competent *E. coli*	Thermo Fisher Scientific	Cat# C600003

**Chemicals, peptides, and recombinant proteins**		

DMEM	Thermo Fisher Scientific	Cat# 11965-092
RPMI 1640	Thermo Fisher Scientific	Cat# 61870-036
IMDM	Thermo Fisher Scientific	Cat# 12440053
Flag antibody coated M2 beads	Sigma Aldrich	Cat# M8823
Triton X-100	Sigma Aldrich	Cat# T8787
TWEEN 20	Sigma Aldrich	Cat# P9416
DMSO	Sigma Aldrich	Cat# D2650
NaCl	Sigma Aldrich	Cat# S5150-1L
Fetal bovine serum	Thermo Fisher Scientific	Cat# 26140079
Penicillin G-streptomycin	Corning	30004CI
Trypsin-EDTA (0.05%)	Thermo Fisher Scientific	25300054
Isopropyl-ß-D-thiogalactopyranoside (IPTG)	Thermo Fisher Scientific	Cat# R0392
ΔSkp2^Fbox^ peptide	Nanjing Yuanpeptide Biotech Co. Ltd (Synthesized)	N/A
MG-132	Target Mol	Cat# T2154
z-VAD-fmk	Target Mol	Cat# T6013
z-IETD-fmk	Target Mol	Cat# T7019
z-DEVD-fmk	Target Mol	Cat# T6005
z-LEHD- fmk	glpbio	Cat# GC18019
Pifitherin-α (PFTα)	Target Mol	Cat# T2707
VX-765	Target Mol	Cat# T6090
PJ-34	Target Mol	Cat# T6197
N-ethylmaleimide (NEM)	Target Mol	Cat# T3088
MLN4924 (Pevonedistat)	Target Mol	Cat# T6332
6-OAP (Brevilin A)	Yuanye biology	Cat# B30102
Protease Inhibitor Cocktail	Cell Signaling Technology, Inc.	Cat# 5871
Phosphatase Inhibitor Cocktail	Cell Signaling Technology, Inc.	Cat# 5870
RNase inhibitors	Thermo Fisher	Cat# EO0381
Isopropanol	DAMAO Co., Ltd.	N/A
Methanol	XIHUA Co., Ltd.	N/A
Ethanol	XIHUA Co., Ltd.	N/A
Acetic acid	Tian in Fuyu Fine Chemicals Co., Litd.	N/A
Lipofectamine™ 3000 Transfection Reagent	Thermo Fisher Scientific	Cat# L3000001
TRIzol™ Reagent	Thermo Fisher Scientific	Cat# 15596026
PBS	Thermo Fisher	C10010500BT
Pre-staining protein marker 10 to 180kDa	Thermo Fisher	Cat# 26617
EDTA	Thermo Fisher	Cat# AM9260
Glycerol	Sigma Aldrich	Cat# G5516-500ML
2-Mercaptoethanol	Sigma Aldrich	Cat# M6250
PVDF membrane	Millipore	Cat# ISEQ00010
Propidium Iodide	Beyotime	Cat# ST511

**Critical commercial assays**		

CellTiter-Glo	Promega	G9241
Caspase-Glo® 3/7 Assay	Promega	G8091
Caspase-Glo® 9 Assay	Promega	G8210
Caspase-Glo® 8 Assay	Promega	G8200
Nuclear and Cytoplasmic Protein Extraction Kit®	Key GEN Biotech	KGP50
Pierce™ BCA Protein Assay Kit	Thermo Fisher Scientific	Cat#23225
ECL Western blotting Kit	Proteintech	Cat# PK10003
EndoFree Mini Plasmid Kit II	TIANGEN	Cat# DP118-02
SYBR Green Supermix	Promega	Cat# S2062

**Experimental models: Cell lines**		

A549	ATCC	CCL-185; RRID:CVCL_0023
H460	ATCC	HTB-177; RRID:CVCL_0459
H1650	ATCC	CRL-5883; RRID:CVCL_1483
95D	ATCC	CRL-2112; RRID:CVCL_3462
LLC	ATCC	CRL-1642; RRID:CVCL_4358
MDA-MB-231	ATCC	HTB-26; RRID:CVCL_0062
Hs578T	ATCC	CRL-7849; RRID:CVCL_0332
MCF-7	ATCC	HTB-22; RRID:CVCL_0031
C4-2B	ATCC	CRL-3315; RRID:CVCL_4784
Du145	ATCC	HTB-81; RRID:CVCL_0105
LNCaP	ATCC	CRL-1740; RRID:CVCL_1379
22RV1	ATCC	CRL-2505; RRID:CVCL_1045
HepG2	ATCC	HB-8065; RRID:CVCL_0027
HCT116	ATCC	CCL-247; RRID:CVCL_0291
U2OS	ATCC	HTB-96; RRID:CVCL_0042
HeLa	ATCC	CCL-2; RRID:CVCL_0030
K562	ATCC	CCL-243; RRID:CVCL_0004
βTC6	ATCC	CRL-11506; RRID:CVCL_0605
H1299	ATCC	CRL-5803; RRID:CVCL_0060
H358	ATCC	CRL-5807; RRID:CVCL_1559
A431	ATCC	CRL-1555; RRID:CVCL_0037
NCI-N87	ATCC	CRL-5822; RRID:CVCL_1603
HEK293	ATCC	CRL-1573; RRID:CVCL_0045

**Oligonucleotides**		

Primers for RT-qPCR (see Table S1)	Igebio company	N/A

**Recombinant DNA**		

pETDuet-Skp1(WT)	This paper	N/A
pETDuet-Skp1(1-140)	This paper	N/A
pETDuet-Skp1(F-box)	This paper	N/A
pETDuet-Skp1(Q97A)	Igebio company	N/A
pETDuet-Skp1(F101A)	Igebio company	N/A
pETDuet-Skp1(V123D)	Igebio company	N/A
pETDuet-Skp1(F139A)	Igebio company	N/A
pETDuet-Skp1(F139D)	Igebio company	N/A
pETDuet-Skp1(I141D)	Igebio company	N/A
pETDuet-Skp1(F101W_V123T)	Igebio company	N/A
pETDuet-Skp1(F101A_F139A)	Igebio company	N/A
pETDuet-Skp1(F101D_F139D)	Igebio company	N/A
pcDNA3.1(+)-Flag-Skp1(WT)	Liu et al., 2015	https://doi.org/10.18632/oncotarget.5547
pcDNA3.1(+)-Flag-Skp1(F101A)	Igebio company	N/A
pcDNA3.1(+)-Flag-Skp1(F139A)	Igebio company	N/A
pcDNA3.1(+)-Flag-Skp1(V123D)	Igebio company	N/A
pcDNA3.1(+)-Flag-Skp1(Q97A)	Igebio company	N/A
pcDNA3.1(+)-mCherry-Skp1(WT)	Igebio company	N/A
pcDNA3.1(+)-mCherry-Skp1(Q97A)	Igebio company	N/A
pcDNA3.1(+)-mCherry-Skp1(V123D)	Igebio company	N/A
pcDNA3.1(+)-mCherry-Skp1(F101A)	Igebio company	N/A
pcDNA3.1(+)-mCherry-Skp1(F139A)	Igebio company	N/A
pcDNA3.1(+)-NIPA-copGFP(WT)	Igebio company	N/A
pcDNA3.1(+)-Skp2-copGFP(WT)	Igebio company	N/A
pcDNA3.1(+)-mCherry-copGFP	Igebio company	N/A

**Software and algorithms**		

ImageJ	ImageJ	https://imagej.nih.gov/ij/
Bio-RAD CFX Manager	BIO-RAD	http://www.bio-rad.com/en-us/product/cfx-manager-software?tab=Download
ZEN	Zeiss	https://www.zeiss.com/microscopy/int/software-cameras.html
Prism 6.0	GraphPad	https://www.graphpad.com/
OriginPro®	OriginLab Corporation	https://www.originlab.com/
InkScape 1.2	InkScape	https://inkscape.org/
PyMOL version 1.8.8.2	PyMol	www.pymol.org
Schrödinger program	Schrödinger, LLC, New York, NY, 2017-1	https://www.schrodinger.com/platform
Amber 14 and Amber Tools	Amber tools	https://ambermd.org/

### Resource availability

#### Lead contact

Further information and requests for resources and reagents should be directed to and will be fulfilled by the lead contact, Jinsong Liu (liu_jinsong@gibh.ac.cn).

#### Materials availability

This work did not generate new unique reagents.

### Experimental model and subject details

#### Cells

HEK293, MDA-MB-231, Hs578T, MCF-7, Hela, βTC6, HepG2, LLC, HCT-116, and A-431 cell lines were cultured in Dulbecco’s Modified Eagle’s Medium (DMEM, Hyclone) supplemented with 10% fetal bovine serum (FBS, Biowest), nonessential amino acids (Gibco), penicillin–streptomycin (HyClone), and GlutaMax (Gibco). A549, H460, H1650, H1299, H358, C4-2B, Du145, LNCaP, 22RV1, Huh-7, MGC-803, MKN-7, and NCI-N87 cells were cultured in Roswell Park Memorial Institute (RPMI) 1640 medium supplemented with 10% FBS (Biowest), nonessential amino acids (Gibco), penicillin–streptomycin (HyClone), and GlutaMax (Gibco). U2OS and K562 cells were cultured in Iscove’s Modified Dulbecco’s Media (*IMDM*) supplemented with 10% FBS (Biowest), nonessential amino acids (Gibco), penicillin–streptomycin (HyClone), and GlutaMax (Gibco). The cultured cells were maintained at 5% CO_2_ in humidified chamber at 37 ⁰C.

#### Plasmids

The pcDNA3.1-flag-*Skp1*, pcDNA3.1-flag-*NIPA*, and pcDNA3.1-flag-*Skp2* plasmids were a kind gift from Prof. Guang-Biao Zhou’s laboratory (Liu et al., 2015). Other plasmids used in this study were generated by Igebio Company (Guangzhou, China) and are listed in key resources table.

### Methods details

#### Small molecule compounds

The compounds (as depicted in Figure S4) tested in this study were already present in our *in house* library. The compounds were either synthesized (JH-1 to JH-12, see the STAR Methods below), or were purchased previously (ZN1 to ZN-12), based on the chemical insights while keeping in mind the hydrophobic architecture of the Skp1 binding site. All the candidate compounds were prepared as stock solutions of 50 mM in DMSO.

#### Materials and general methods for compound(s) synthesis

All commercially available compounds and solvents were of reagent grade and used without further treatment unless otherwise noted. Reactions were monitored by TLC using Qing Dao Hai Yang GF254 silica gel plates (5 × 10 cm); zones were detected visually under ultraviolet irradiation (254 nm) and by spraying with an ethanol solution of phosphomolybdic acid, or by fuming with iodine steam. Silica gel column chromatography was performed on silica gel (200–300 mesh) from Qing Dao Hai Yang. NMR spectra were recorded on a Bruker NMR AVANCE 400 or a Bruker NMR AVANCE 500 (shown in Data S1). Chemical shifts (δ) were recorded in ppm and coupling constants (*J*) in hertz (Hz). Splitting patterns describe apparent multiplicities and are designated as s (singlet), d (doublet), t (triplet), q (quartet), m (multiplet) or br (broad). MS data were measured on an Agilent MSD-1200 ESI-MS system.

4-Bromo-2-(4-methoxyphenyl)pyridine (JH-1): A mixture of 2,4-dibromopyridine **S1** (2.37 g, 10 mM), 4-methoxyphenylboronic acid pinacol ester **S2** (2.57 g, 11 mM), Pd(OAc)_2_ (113 mg, 0.5 mM), PPh_3_ (525 mg, 2 mM), and KOH (1.12 g, 20 mM) were dissolved in CH_3_CN (100 mL). The mixture was stirred at 70°C under argon atmosphere for 24 hrs. After cooled to room temperature, the solid was filtrated off and the filtrate was concentrated. The crude product was then dissolved in CH_2_Cl_2_ (200 mL) and the solution was washed with water (100 mL×3) and brine (100 mL), dried over Na_2_SO_4_, filtered and evaporated in vacuo. The resulting residue was purified by column chromatography on silica gel (petroleum ether/EtOAc, 50/1 then 20/1) to give **JH-1** (7.02 g, 89%) as a colorless solid. ^1^H NMR (400 MHz, CDCl_3_) δ 8.45 (d, J = 5.2 Hz, 1H), 7.92 (d, J = 8.4 Hz, 2H), 7.83 (d, J = 1.4 Hz, 1H), 7.33 (dd, J1 = 1.4 Hz, J2 = 5.2 Hz, 1H), 6.99 (d, J = 8.4 Hz, 2H), 3.86 (s, 3H).

**Figure:**
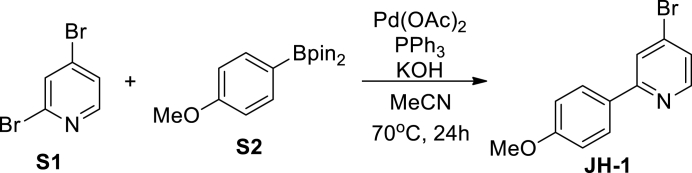

3-(2-(4-Methoxyphenyl)pyridin-4-yl)aniline (JH-3): A mixture of 4-bromo-2-(4-methoxyphenyl)pyridine **JH-1** (1.05 g, 4 mM), 3-nitrophenylboronic acid **S3** (735 mg, 4.4 mM), Pd(PPh_3_)_4_ (347 mg, 0.3 mM), and 1 M aqueous NaHCO_3_ (12 mL, 12 mM) were dissolved in dimethoxyethane (60 mL). The mixture was refluxed overnight under argon atmosphere. After cooled to room temperature, the solid was filtrated off and the filtrate was concentrated. The crude product was then dissolved in EtOAc (100 mL) and the solution was washed with water (50 mL×3) and brine (50 mL), dried over Na_2_SO_4_, filtered and evaporated in vacuo. The resulting residue was purified by column chromatography on silica gel (petroleum ether/EtOAc, 10/1) to give **JH-2** (793 mg, 65%) as a yellow solid. ^1^H NMR (400 MHz, CDCl_3_) δ 8.70 (d, J = 5.1 Hz, 1H), 8.48 (s, 1H), 8.25 (dd, J1 = 1.2 Hz, J2 = 8.2 Hz, 1H), 7.99–7.95 (m, 3H), 7.82 (s, 1H), 7.64 (t, J = 8.0 Hz, 1H), 7.35 (dd, J1 = 1.4 Hz, J2 = 5.1 Hz, 1H), 6.98 (d, J = 8.8 Hz, 2H), 3.83 (s, 3H). ^13^C NMR (126 MHz, CDCl_3_) δ 160.8, 158.1, 150.4, 148.8, 146.6, 140.4, 133.0, 131.5, 130.2, 128.3, 123.6, 122.0, 119.3, 117.6, 114.2, 55.4.

**Figure:**
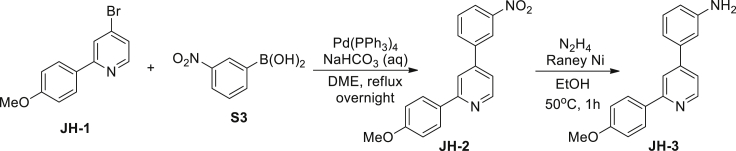

To a solution of **JH-2** (312 mg, 1 mM) in ethanol (10 mL) was added hydrazine hydrate (80% in water, 1 mL). The reaction was stirred at 50°C for 15 min and an excess of Raney nickel slurry in water (about 1.2 equiv) was added slowly. After 1 hr, the bubbling ceased and the mixture was cooled to room temperature and filtered through Celite. The filtrate was condensed under reduced pressured, and the residue was purified by column chromatography on silica gel (petroleum ether/EtOAc, 5/1) to give **JH-3** (194 mg, 69%) as a colorless solid. ^1^H NMR (400 MHz, CDCl_3_) δ 8.65 (d, J = 5.1 Hz, 1H), 8.00 (d, J = 8.8 Hz, 1H), 7.81 (s, 1H), 7.31 (dd, J1 = 1.3 Hz, J2 = 5.1 Hz, 1H), 7.25 (t, J = 7.8 Hz, 1H), 7.05–6.99 (m, 3H), 6.93 (s, 1H), 6.73 (dd, J1 = 1.3 Hz, J2 = 8.0 Hz, 1H), 3.84 (s, 3H), 3.84 (brs, NH_2_). ^13^C NMR (126 MHz, CDCl_3_) δ 160.5, 157.5, 149.8, 147.2, 139.8, 132.1, 130.0, 128.3, 119.6, 118.0, 117.2, 115.6, 114.1, 113.4, 55.3.

N-(3-(2-(4-methoxyphenyl)pyridin-4-yl)phenyl)acetamide (JH-4): 3-(2-(4-Methoxyphenyl)pyridin-4-yl)aniline **JH-3** (76 mg, 0.28 mM) was dissolved in Ac_2_O (6 mL). After stirred at room temperature under argon atmosphere for 2 hrs, the solution was condensed under reduced pressured. The crude product was then dissolved in CH_2_Cl_2_ (100 mL) and the solution was washed with saturated NaHCO_3_ aqueous solution (50 mL×3) and brine (50 mL), dried over Na_2_SO_4_, filtered and evaporated in vacuo. The resulting residue was purified by column chromatography on silica gel (petroleum ether/EtOAc, 2/1 then 1/2) to give **JH-4** (85 mg, 97%) as a colorless solid. ^1^H NMR (400 MHz, CDCl_3_) δ 8.64 (d, J = 5.1 Hz, 1H), 7.96 (d, J = 8.8 Hz, 2H), 7.88–7.86 (m, 2H), 7.81 (s, 1H), 7.56–7.54 (m, 1H), 7.43–7.38 (m, 2H), 7.33 (dd, J1 = 1.5 Hz, J2 = 5.1 Hz, 1H), 6.98 (d, J = 8.8 Hz, 2H), 3.85 (s, 3H), 2.19 (s, 3H). ^13^C NMR (126 MHz, CDCl_3_) δ 168.8, 160.7, 157.9, 150.0, 148.9, 139.6, 138.9, 132.1, 129.8, 128.4, 123.0, 120.4, 119.8, 118.6, 118.2, 114.3, 55.5, 24.7. MS (ESI): m/z 319.3 [M + H]^+^.

**Figure:**
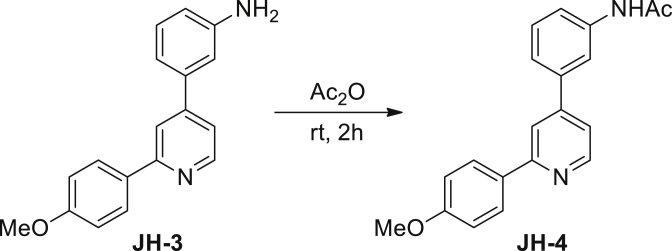

3-(3-(2-(4-methoxyphenyl)pyridin-4-yl)phenylamino)propane-1,2-diol (JH-5): A mixture of 3-(2-(4-Methoxyphenyl)pyridin-4-yl)aniline **JH-3** (194 mg, 0.73 mM), *(±)*-glycidol (54 mg, 0.73 mM), and Ti(O^*i*^Pr)_4_ (260 μL, 0.88 mM) were dissolved in dry CH_2_Cl_2_ (10 mL). The mixture was stirred at room temperature overnight under argon atmosphere. After quenched by 10% NaOH aqueous solution, the mixture was filtered through Celite. The organic layer was dried over Na_2_SO_4_, filtered and evaporated in vacuo. The resulting residue was purified by column chromatography on silica gel (petroleum ether/EtOAc, 2/1, then EtOAc) to give **JH-5** (15 mg, 28% (brsm)) as a colorless solid. ^1^H NMR (400 MHz, CDCl_3_) δ 8.63 (d, J = 5.1 Hz, 1H), 7.96 (d, J = 8.7 Hz, 2H), 7.81 (s, 1H), 7.34–7.27 (m, 2H), 7.03–6.99 (m, 3H), 6.88 (s, 1H), 6.73–6.70 (m, 1H), 4.02–3.98 (m, 1H), 3.86 (s, 3H), 3.81–3.77 (m, 1H), 3.69–3.64 (m, 1H), 3.37–3.33 (m, 1H), 3.26–3.21 (m, 1H). ^13^C NMR (126 MHz, CDCl_3_) δ 160.7, 157.8, 149.9, 149.8, 148.9, 140.0, 132.2, 130.2, 128.5, 119.9, 118.4, 117.0, 114.3, 113.9, 111.9, 70.5, 64.9, 55.5, 46.8. MS (ESI): m/z 349.2 [M−H]^-^.

**Figure:**
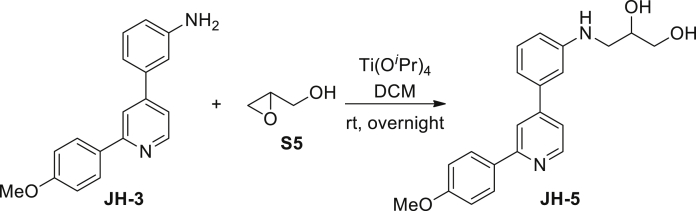

N-(4-(2-(4-methoxyphenyl)pyridin-4-yl)phenyl)acetamide (JH-6): A mixture of 4-bromo-2-(4-methoxyphenyl)pyridine **JH-1** (264 mg, 1 mM), 4-acetamidophenyl boronic acid **S6** (197 mg, 1.1 mM), Pd(PPh_3_)_4_ (87 mg, 0.075 mM), and 1 M aqueous NaHCO_3_ (3 mL, 3 mM) were dissolved in dimethoxyethane (10 mL). The mixture was refluxed overnight under argon atmosphere. After cooled to room temperature, the solid was filtrated off and the filtrate was concentrated. The crude product was then dissolved in EtOAc (20 mL) and the solution was washed with water (10 mL×3) and brine (10 mL), dried over Na_2_SO_4_, filtered and evaporated in vacuo. The resulting residue was purified by column chromatography on silica gel (petroleum ether/EtOAc, 10/1) to give **JH-6** (191 mg, 60%) as a colorless solid. ^1^H NMR (400 MHz, CDCl_3_) δ 8.66 (d, J = 5.1 Hz, 1H), 8.48 (s, 1H), 8.25 (dd, J1 = 1.2 Hz, J2 = 8.2 Hz, 1H), 7.99–7.95 (m, 3H), 7.98 (d, J = 8.8 Hz, 2H), 7.83 (s, 1H), 7.66–7.62 (m, 5H), 7.36 (dd, J1 = 1.6 Hz, J2 = 5.1 Hz, 1H), 7.01 (d, J = 8.8 Hz, 2H), 3.87 (s, 3H), 2.20 (s, 3H). ^13^C NMR (126 MHz, CDCl_3_) δ 168.6, 160.7, 157.9, 150.0, 148.6, 139.0, 134.3, 132.2, 128.4, 127.8, 120.3, 119.4, 117.8, 114.3, 55.5, 24.8. MS (ESI): m/z 317.3 [M−H]^-^.

**Figure:**
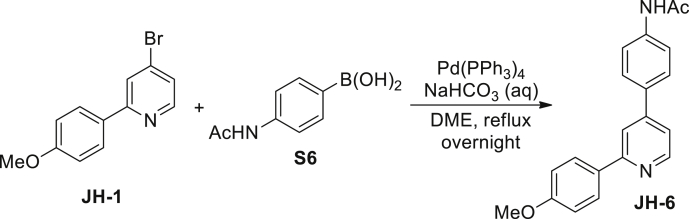

N-(4-(3-(4-methoxyphenyl)phenyl)phenyl)acetamide (JH-7): A mixture of 1-bromo-3-iodobenzene **S7** (566 mg, 2 mM), 4-methoxyphenyl boronic acid **S8** (319 mg, 2.1 mM), Pd(PPh_3_)_4_ (232 mg, 0.2 mM), and K_2_CO_3_ (1.38 g, 10 mM) were dissolved in 9:1 (v/v) mixed dioxane and water (30 mL). The mixture was refluxed under argon atmosphere for 6 hrs. After cooled to room temperature, 4-acetamidophenyl boronic acid **S6** (376 mg, 2.1 mM) was added. The mixture was refluxed overnight under argon atmosphere. The solid was filtrated off and the filtrate was concentrated. The crude product was then dissolved in CH_2_Cl_2_ (20 mL) and the solution was washed with water (10 mL×3) and brine (10 mL), dried over Na_2_SO_4_, filtered and evaporated in vacuo. The resulting residue was purified by column chromatography on silica gel (petroleum ether/EtOAc, 1/1) to give **JH-7** (225 mg, 35%) as a colorless solid. ^1^H NMR (400 MHz, CDCl_3_) δ 7.73 (s, 1H), 7.60–7.44 (m, 9H), 7.39 (brs, NH), 3.86 (s, 3H), 2.21 (s, 3H). ^13^C NMR (126 MHz, CDCl_3_) δ 168.5, 159.4, 141.5, 141.1, 137.4, 133.8, 129.3, 128.4, 127.8, 125.7, 125.5, 125.4, 120.3 114.4, 55.5, 24.8. MS (ESI): m/z 340.5 [M + Na]^+^. (1,3-di-(4-methoxyphenyl)benzene **JH-8** (155 mg, 27%) was also obtained as a by-product).

**Figure:**
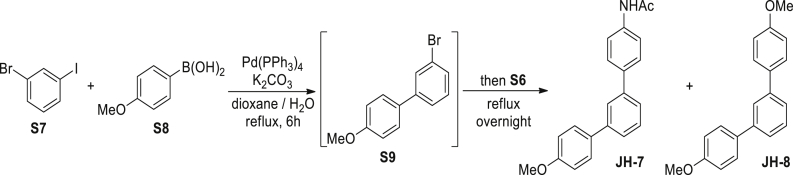

3-(2-(3-Methoxyphenyl)pyridin-4-yl)aniline (JH-9): A mixture of 2,4-dibromopyridine **S1** (237 mg, 1 mM), 3-methoxyphenylboronic acid pinacol ester **S11** (258 mg, 1.1 mM), Pd(OAc)_2_ (12 mg, 0.05 mM), PPh_3_ (53 mg, 0.2 mM), and KOH (112 mg, 2 mM) were dissolved in CH_3_CN (20 mL). The mixture was stirred at 70°C under argon atmosphere for 24 hrs. After cooled to room temperature, the solid was filtrated off and the filtrate was concentrated. The crude product was then dissolved in CH_2_Cl_2_ (20 mL) and the solution was washed with water (10 mL×3) and brine (20 mL), dried over Na_2_SO_4_, filtered and evaporated in vacuo. The resulting residue was purified by column chromatography on silica gel (petroleum ether/EtOAc, 50/1 then 20/1) to give **S12** (205 mg, 78%) as a colorless solid. ^1^H NMR (400 MHz, CDCl_3_) δ 8.47 (d, J = 5.3 Hz, 1H), 7.87 (d, J = 1.5 Hz, 1H), 7.56–7.54 (m, 1H), 7.51–7.48 (m, 1H), 7.38–7.34 (m, 2H), 6.99–6.96 (m, 1H), 3.87 (s, 3H). ^13^C NMR (126 MHz, CDCl_3_) δ 160.2, 158.7 150.3, 139.5, 133.5, 129.9, 125.4, 124.0, 119.4, 115.8, 112.3, 55.4.

**Figure:**
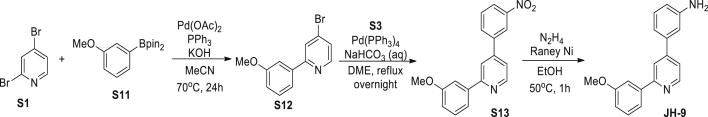

A mixture of 4-bromo-2-(3-methoxyphenyl)pyridine **S12** (205 mg, 0.78 mM), 3-nitrophenyl boronic acid **S3** (143 mg, 0.85 mM), Pd(PPh_3_)_4_ (70 mg, 0.06 mM), and 1 M aqueous NaHCO_3_ (2.4 mL, 2.4 mM) were dissolved in dimethoxyethane (15 mL). The mixture was refluxed overnight under argon atmosphere. After cooled to room temperature, the solid was filtrated off and the filtrate was concentrated. The crude product was then dissolved in EtOAc (10 mL) and the solution was washed with water (10 mL×3) and brine (10 mL), dried over Na_2_SO_4_, filtered and evaporated in vacuo. The crude product **S13** was dissolved in ethanol (10 mL), and hydrazine hydrate (80% in water, 0.5 mL) was added. The reaction was stirred at 50°C for 15 min and an excess of Raney nickel slurry in water (about 1.2 equiv) was added slowly. After 1 hr, the bubbling ceased and the mixture was cooled to room temperature and filtered through Celite. The filtrate was condensed under reduced pressured, and the residue was purified by column chromatography on silica gel (petroleum ether/EtOAc, 3/1) to give **JH-9** (116 mg, 54% for 2 steps) as a colorless solid. ^1^H NMR (400 MHz, CDCl_3_) δ 8.71 (d, J = 5.1 Hz, 1H), 7.88 (d, J = 0.6 Hz, 1H), 7.62–7.58 (m, 2H), 7.43–7.38 (m, 2H), 7.29 (t, *J* = 7.8 Hz, 1H), 7.07 (d, J = 7.7 Hz, 1H), 7.00–6.98 (m, 2H), 6.93 (s, 1H), 6.73 (dd, J1 = 1.6 Hz, J2 = 7.9 Hz, 1H), 3.91 (s, 3H).

3-(2-(2-Methoxyphenyl)pyridin-4-yl)aniline (JH-10): A mixture of 2,4-dibromopyridine **S1** (1.185 g, 5 mM), 2-methoxyphenylboronic acid pinacol ester **S14** (1.288 g, 5.5 mM), Pd(OAc)_2_ (56 mg, 0.25 mM), PPh_3_ (263 mg, 1 mM), and KOH (560 mg, 10 mM) were dissolved in CH_3_CN (100 mL). The mixture was stirred at 70°C under argon atmosphere for 24 hrs. After cooled to room temperature, the solid was filtrated off and the filtrate was concentrated. The crude product was then dissolved in CH_2_Cl_2_ (50 mL) and the solution was washed with water (50 mL×3) and brine (50 mL), dried over Na_2_SO_4_, filtered and evaporated in vacuo. The resulting residue was purified by column chromatography on silica gel (petroleum ether/EtOAc, 50/1 then 20/1) to give **S15** (1.14 g, 86%) as a colorless solid. ^1^H NMR (400 MHz, CDCl_3_) δ 8.49 (d, J = 5.3 Hz, 1H), 8.04 (d, J = 1.6 Hz, 1H), 7.79 (dd, J1 = 1.7 Hz, J2 = 7.6 Hz, 1H), 7.39–7.34 (m, 2H), 7.09–7.05 (m, 1H), 6.97 (d, J = 8.3 Hz, 1H), 3.85 (s, 3H). ^13^C NMR (126 MHz, CDCl_3_) δ 157.3, 156.9, 149.9, 132.3, 131.2, 130.6, 128.3, 127.6, 124.8, 121.0, 111.4, 55.6.

**Figure:**
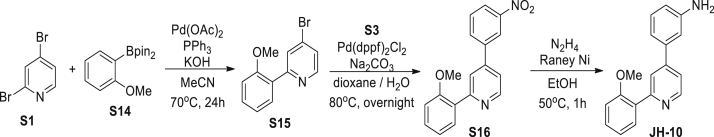

A mixture of 4-bromo-2-(2-methoxyphenyl)pyridine **S15** (923 mg, 3.5 mM), 3-nitrophenyl boronic acid **S3** (700 mg, 4.2 mM), Pd(dppf)_2_Cl_2_ (542 mg, 0.47 mM), and Na_2_CO_3_ (1.113 g, 10.5 mM) were dissolved in 1:1 (v/v) mixed dioxane and water (50 mL). The mixture was stirred at 80°C overnight under argon atmosphere. After cooled to room temperature, the solid was filtrated off and the filtrate was concentrated. The crude product was then dissolved in CH_2_Cl_2_ (50 mL) and the solution was washed with water (30 mL×3) and brine (50 mL), dried over Na_2_SO_4_, filtered and evaporated in vacuo. The resulting residue was purified by column chromatography on silica gel (petroleum ether/EtOAc, 10/1) to give **S16** (587 mg, 55%) as a colorless solid. ^1^H NMR (500 MHz, CDCl_3_) δ 8.82 (d, J = 5.1 Hz, 1H), 8.54 (d, J = 1.7 Hz, 1H), 8.31 (dd, J1 = 1.3 Hz, J2 = 8.2 Hz, 1H), 8.08 (d, J = 1.1 Hz, 1H), 8.00 (d, J = 7.7 Hz, 1H), 7.83 (dd, J1 = 1.6 Hz, J2 = 7.6 Hz, 1H), 7.69 (t, J = 3.0 Hz, 1H), 7.47–7.40 (m, 2H), 7.12 (t, J = 7.4 Hz, 1H), 7.05 (d, J = 8.3 Hz, 1H), 3.90 (s, 3H). MS (ESI): m/z 307.4 [M + H]^+^.

To a solution of **S16** (587 mg, 1.92 mM) in ethanol (30 mL) was added hydrazine hydrate (80% in water, 3.5 mL). The reaction was stirred at 50°C for 15 min and an excess of Raney nickel slurry in water (about 1.2 equiv) was added slowly. After 1 hr, the bubbling ceased and the mixture was cooled to room temperature and filtered through Celite. The filtrate was condensed under reduced pressured, and the residue was purified by column chromatography on silica gel (petroleum ether/EtOAc, 5/1 then 2/1) to give **JH-10** (477 mg, 90%) as a yellow solid. ^1^H NMR (400 MHz, CDCl_3_) δ 8.71 (d, J = 5.1 Hz, 1H), 7.98 (d, J = 0.7 Hz, 1H), 7.77 (dd, J1 = 1.6 Hz, J2 = 7.6 Hz, 1H), 7.40–7.37 (m, 2H), 7.29–7.25 (m, 1H), 7.11–7.02 (m, 3H), 6.97 (t, J = 1.8 Hz, 1H), 6.76 (dd, J1 = 1.5 Hz, J2 = 8.0 Hz, 1H), 3.87 (s, 3H), 3.80 (brs, NH_2_). MS (ESI): m/z 277.4 [M + H]^+^.

N-(3-(2-(4-methylphenyl)pyridin-4-yl)phenyl)acetamide (JH-11): A mixture of 2,4-dibromopyridine **S1** (474 mg, 2 mM), 4-methylphenylboronic acid pinacol ester **S17** (480 mg, 2.2 mM), Pd(OAc)_2_ (23 mg, 0.1 mM), PPh_3_ (105 mg, 0.4 mM), and KOH (224 mg, 4 mM) were dissolved in CH_3_CN (20 mL). The mixture was stirred at 70°C under argon atmosphere for 24 hrs. After cooled to room temperature, the solid was filtrated off and the filtrate was concentrated. The crude product was then dissolved in CH_2_Cl_2_ (20 mL) and the solution was washed with water (20 mL×3) and brine (20 mL), dried over Na_2_SO_4_, filtered and evaporated in vacuo. The resulting residue was purified by column chromatography on silica gel (petroleum ether/EtOAc, 50/1 then 20/1) to give **S18** (209 mg, 42%) as a colorless solid. ^1^H NMR (400 MHz, CDCl_3_) δ 8.48 (d, J = 5.3 Hz, 1H), 7.88–7.85 (m, 3H), 7.37 (dd, J1 = 1.6 Hz, J2 = 5.3 Hz, 1H), 7.29–7.27 (m, 2H), 2.41 (s, 3H).

**Figure:**
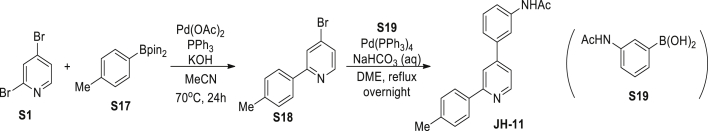

A mixture of 4-bromo-2-(4-methylphenyl)pyridine **S18** (207 mg, 0.8 mM), 3-acetamidophenyl boronic acid **S19** (156 mg, 0.88 mM), Pd(PPh_3_)_4_ (93 mg, 0.08 mM), and 1 M aqueous NaHCO_3_ (3 mL, 3 mM) were dissolved in dimethoxyethane (10 mL). The mixture was refluxed overnight under argon atmosphere. After cooled to room temperature, the solid was filtrated off and the filtrate was concentrated. The crude product was then dissolved in EtOAc (20 mL) and the solution was washed with water (10 mL×3) and brine (10 mL), dried over Na_2_SO_4_, filtered and evaporated in vacuo. The resulting residue was purified by column chromatography on silica gel (petroleum ether/EtOAc, 10/1) to give **JH-11** (84 mg, 35%) as a colorless solid. ^1^H NMR (400 MHz, CDCl_3_) δ 8.66 (d, J = 5.1 Hz, 1H), 8.48 (s, 1H), 8.25 (dd, J1 = 1.2 Hz, J2 = 8.2 Hz, 1H), 7.99–7.95 (m, 3H), 7.98 (d, J = 8.8 Hz, 2H), 7.83 (s, 1H), 7.66–7.62 (m, 5H), 7.36 (dd, J1 = 1.6 Hz, J2 = 5.1 Hz, 1H), 7.01 (d, J = 8.8 Hz, 2H), 3.87 (s, 3H), 2.20 (s, 3H). ^13^C NMR (126 MHz, CDCl_3_) δ 168.6, 160.7, 157.9, 150.0, 148.6, 139.0, 134.3, 132.2, 128.4, 127.8, 120.3, 119.4, 117.8, 114.3, 55.5, 24.8. MS (ESI): m/z 317.3 [M−H]^-^.

6-phenylphenanthridine (JH-12): To a solution of 9H-fluoren-9-one (1.08 g, 6 mM) in dry THF (60 mL) was added 3 M phenyl magnesium bromide in THF (3 mL, 9 mM). The reaction was stirred at room temperature under argon atmosphere for 1 hr. After quenched by saturated NH_4_Cl aqueous solution (50 mL), the organic layer was dried over Na_2_SO_4_, filtered and evaporated in vacuo. The resulting residue was purified by column chromatography on silica gel (petroleum ether/EtOAc, 30/1) to give **S20** (1.64 g, 88%) as a colorless solid. ^1^H NMR (500 MHz, CDCl_3_) δ 7.67 (d, J = 7.6 Hz, 1H), 7.39–7.32 (m, 6H), 7.27–7.21 (m, 5H), 2.49 (s, 1H). MS (ESI): m/z 257.2 [M−H]^-^.

**Figure:**
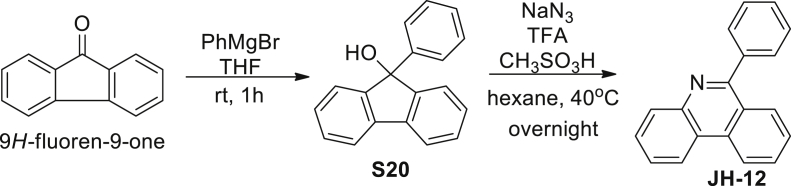

A mixture of 9-phenyl-9H-fluoren-9-ol **S20** (258 mg, 1 mM), sodium azide (163 mg, 2.5 mM), TFA (0.9 mL, 12 mM), and MeSO_3_H (390 μL, 6 mM) were dissolved in hexane (20 mL). The mixture was stirred at 40°C overnight under argon atmosphere. After cooled to room temperature, the reaction was quenched by 2M NaOH aqueous solution (20 mL) and filtered through Celite. The filtrate was extracted by EtOAc (30 mL) and water (20 mL×3), and the organic layer was washed by brine (10 mL), dried over Na_2_SO_4_, filtered and evaporated in vacuo. The resulting residue was purified by column chromatography on silica gel (petroleum ether/EtOAc, 30/1) to give **JH-12** (209 mg, 82%) as a colorless solid. ^1^H NMR (400 MHz, CDCl_3_) δ 8.67 (d, J = 8.3 Hz, 1H), 8.59 (d, *J* = 8.1 Hz, 1H), 8.29 (d, J = 8.1 Hz, 1H), 8.11 (d, J = 8.2 Hz, 1H), 7.85–7.75 (m, 4H), 7.70–7.66 (m, 1H), 7.61–7.53 (m, 4H).

#### Protein expression and purification

A pETDuet-1 plasmid was used for expressing different forms of Skp1, including wild type (Skp1^WT^), truncate (ΔSkp1^1−140^), Skp1-F-box (fused), and mutants (Skp1^Mut^), as a His6-fusion protein(s) in *Escherichia coli* BL21 (DE3) cells. All the desired plasmids were constructed using the *BamH1* and *HindIII* restriction sites of the pETDuet-1 expression vector, and are mentioned in key resources table. The constructed plasmids were then transformed into *E.coli* BL21 (DE3) and single colonies were selected on Ampicillin (100 μg mL^−1^) containing plates. For expressing protein, a single freshly transformed host colony was grown in a starter culture (20 mL of LB media containing 100 μg mL^−1^ Ampicillin) at 37°C for 15 hr. The culture was then used to inoculate 2L LB media (containing 100 μg mL^−1^ Ampicillin) and grown at 37°C with shaking to an OD_600_ of 1.0 and then cooled down to 16°C before overnight induction with 0.5 mM isopropyl-b-D-thiogalactopyranoside (IPTG). The cells were harvested by centrifugation (4,000 rpm, 20 min, and 4°C) using a Beckman Coulter Avanti J-20 XP centrifuge.

Cell pellets were re-suspended in lysis buffer (50 mM Tris-HCl pH 8.0, 500 mM NaCl, 20 mM imidazole, 7 mM β-mercaptoethanol, and 1 mM phenylmethylsulfonyl fluoride (PMSF)) (buffer A). All subsequent operations were done at 4°C. Cellular disruption was achieved by 3 to 4 passages through a Low Temperature Ultra-high Pressure Cell Disrupter JN-3000 plus (JNBIO, China) and the cell debris was removed by centrifugation at 35,000 g for 30 min. Chromatography was conducted using an Akta FPLC (GE Healthcare). The lysate was loaded onto a HisTrap^TM^ HP column (GE Healthcare) equilibrated with buffer A. The column was washed using 20 column volumes of buffer A and the target protein (∼20 KDa) was eluted with a 5% - 40% concentration linear-gradient of buffer B (50 mM Tris-HCl pH 8, 500 mM NaCl, 500 mM imidazole, 7 mM ß-mercaptoethanol, 1 mM PMSF). The polypeptide compositions of the fractions were monitored by 15% SDS-PAGE gel. The appropriate protein fractions were pooled and loaded onto a Source-Q column equilibrated with buffer C (20 mM Tris-HCl pH 8.0, 50 mM NaCl, and 2 mM Dithiothreitol (*DTT*)) and eluted with buffer D (20 mM Tris-HCl pH 8.0, 2M NaCl) with a linear gradient (15% to 35%). The protein fractions were again monitored by SDS-PAGE, pooled, and gel filtered through a Superdex-75 size exclusion column equilibrated with buffer E (20 mM Tris-HCl pH 8.0, 150 mM NaCl, 2 mM DTT). The peak fractions were pooled, concentrated by centrifugal ultrafiltration, and stored at −80°C. The protein concentrations were determined using the Bio-Rad dye reagent with bovine serum albumin as the standard.

All the pETDuet-1^Mut^ and pETDuet-1^truncate^ plasmids modified, from pETDuet-1^WT^ during this study were generated by Igebio company (Guangzhou, China) (as mentioned in key resources table), while expression and purification was performed using the same protocol as described above.

#### Fluorescence polarization (FP) assay

The basic principle of a FP assay relies on a “small” fluorescence-labeled tracer and a “large” binding partner (Lea and Simeonov, 2011). In solution, the tracer tumbles and unpolarized light is emitted after excitation. However, when bound by a binding partner, the tumbling is reduced and more polarized light can be measured after excitation. In this work, the FP assay was conducted based on fluorescence signal differences between free and protein-bound fluorescein-labeled peptide.

##### Tracer construction

The C-terminally fluorescently-labeled F-box peptide (tracer) comprising 50 amino acids (RENFPGVSWDSLPDELLLGIFSCLCLPELLKVSGVCKRWYRLASDESLWQ-FAM) was purchased from Yuanpeptide. This tracer was constructed on the consensus sequence of Skp2-F-box motif domain (PDB: 2AST, chain B).

##### Circular Dichroism (CD) spectroscopy

Since our tracer was a full F-box peptide, we first assessed its secondary structure by CD spectroscopy. CD spectra were obtained with a Jasco J-815 spectrophotometer (JASCO, Easton, MD, USA). Samples were prepared in 2 mM Na_2_H_2_PO_4_ prepared with two different pH (8.0 and 8.5). Data were recorded from 200 to 260 nm with a scanning speed of 20 nm/min and a bandwidth at 1.0 nm in a 0.1 cm path-length quartz cell. The resulting data was converted to per-residue molar ellipticity units, [Θ] (deg cm^2^ dmol^−1^ residue^−1^), and the secondary structure content was analyzed with the Dichroweb software package. The reader may refer to Figure S3A for the final analysis.

##### FP measurements

FP measurements were performed using an EnVision multilabel Reader (Perkin Elmer). Excitation wavelength was 485 nm, and emission was detected at 535 nm. Lyophilized F-box-FAM peptide was initially dissolved in a Tris buffer (20 mM Tris-HCl pH 9.0, 150 mM NaCl, 2 mM DTT), and then a stock solution of 100 μM was prepared by subsequent dilutions in a FP buffer (20 mM Tris-HCl pH 8.0, 150 mM NaCl, 2 mM DTT, 5% (v/v) glycerol, 0.001% (v/v) Triton X-100). Specific controls groups included free F-box-FAM (tracer), bound F-box-FAM in the presence of protein (Skp1^WT^ or ΔSkp1^1−140^), and FP buffer for every measurement, allowing accurate estimation of specific polarization.

##### Direct binding of F-box peptide to Skp1

The F-box-FAM peptide (0.5 μM) was incubated with the increasing amounts of Skp1 (10 nM to 100 uM) in a final volume of 20 μL binding buffer (20 mM Tris-HCl pH 8.0, 150 mM NaCl, 2 mM DTT, 5% (v/v) glycerol, 0.001% (v/v) Triton X-100) in black Costar 384-well plate at room temperature for 1 hr in the dark. The fluorescence anisotropy was measured. The binding dissociation constant (K_D_) was determined with non-linear regression and fitting to the following equation using GraphPad Prism® v6.0 (San Diego, CA). Y = Bmax ×X/(KD + X). Experiments were performed in quadruplicates in three independent experiments. In order to assure the specific binding of the tracer at the F-box binding interface of Skp1, we conducted the Skp1^WT^: tracer and ΔSkp1^1−140^: tracer reactions at a fixed protein (25 μM):tracer (500 nM) ratio. The 25 μM protein concentration was selected because it gave a sufficient difference in FP values (assay window ∼76 mP) from binding saturation experiments. The other assay conditions were kept same as above. Moreover, the same experiment with the same conditions was carried out by adding previously reported Skp1 inhibitor, 6-OAP. The experiments were performed in quadruplicates in three independent experiments.

##### Assay quality

One important criterion reflecting the suitability of a high throughput screening (HTS) assay is the Z′ factor, which can be calculated from a number of repeated reactions to determine whether the response is large enough to obtain reliable data. In order to provide a measure of the quality of our FP assay, we calculated the Z′ factor from the mean signals of the positive and negative controls (μ_c+_ and μ_c−_) and their standard deviations (σ_c+_ and σ_c−_) using the formula Z′ = 1−[3(σ_c+_ + σ_c−_)/Iμ_c+_ − μ_c−_I] (Zhang et al., 1999). As the signal range increases and the variations decrease, Z′ tends to the ideal limit of 1, while Z′ values above 0.5 are considered “excellent” (Zhang et al., 1999). From four independent measurements of the positive (F-box tracer plus Skp1^WT^) and negative (F-box tracer alone) controls for the FP assay, the Z′ factor is 0.73. This indicated that our Skp1:F-box-tracer FP assay could be used as a screening platform to test selected subset of ligands in this study.

##### Competition experiments

The selected subset of compounds were initially assayed at final concentration of 200 μM. The compounds were incubated at room temperature with Skp1^WT^ for 1 hr, followed by the addition of the tracer. The final concentrations in the reaction were: 200 μM of each of the tested compound, 25 μM Skp1^WT^, and 500 nM tracer. After incubation for another 2 hrs, FP signals was measured on an EnVision multilabel Reader. The FP response was measured in quadruplicates with controls including free F-box-FAM (tracer), bound Skp1^WT^-F-box-FAM, and FP buffer. The resultant FP values were plotted as bar graphs using ORIGIN Lab software.

For gradient FP assay, protein samples were mixed with an appropriate concentrations of ligand over the range of several folds. Skp1^WT^ (25 μM) and tracer (500 nM) concentrations were kept constant. The IC_50_ value of the compound in the gradient-concentration assay was determined with nonlinear regression and fitting using the following equation in GraphPad Prism® v6.0 (San Diego, CA). Y = Bottom + (Top – Bottom)/(1 + 10^X−LogIC50^). The Ki value was then calculated by using a Ki calculator (http://websites.umich.edu/∼shaomengwanglab/software/calc_ki/index.html) that is based on the method described previously (Nikolovska-Coleska et al., 2004). In short, the relationship between the IC_50_ of the inhibitor and the Ki of the inhibitor depends on the K_D_ of the fluorescent ligand and the concentration of the receptor.

#### Thermal stability shift assay (TSA)

TSA, also known as differential scanning fluorimetry (DSF), is used to identify the contribution of a ligand binding to an unfolding protein as a function of temperature. The detection involves increase in fluorescence of a fluorophore SYPRO Orange (Invitrogen) upon binding to the hydrophobic regions of the gradually unfolded protein. A ligand stabilizing a protein may increase the melting temperature (Tm). To detect the binding of Z0933M with Skp1, a gradient TSA was used, in which, protein samples were mixed with appropriate concentrations of ligand over the range of several folds. The protein (Skp1^WT^ or ΔSkp1^1−140^) was kept constant at 8 μM, and the compound concentration was varied from 50 μM to 600 μM. For comparison with 6-OAP, a single concentration of 400 μM was tested with both Skp1^WT^ and ΔSkp1^1−140^. All reactions were set up in final volume of 10 μL in 96-well plate with 1x SYPRO Orange (Invitrogen) and incubated with compounds on ice for 30 min. According to the experimental protocol, samples were heated at 0.5°C per minute, ranging from 30 to 90°C, and the fluorescence intensity was measured at the interval of 0.5°C. Appropriate buffer blank and protein-only controls were kept, and the shift in Tm was measured as ΔTm = Tm (ligand-protein) ‒ (protein-only). The resultant ΔTm values were plotted by using GraphPad Prism® v6.0 (San Diego, CA) and ORIGIN Lab softwares.

#### Surface plasmon resonance imaging (SPRi) assay

##### Chip preparation

Freshly deposited gold coated standard SPRi chips/slides (Plexera, LLC) were cleaned, and compound(s) at 10 mM concentration in 100% DMSO were spotted on the chips using a micropipette. After this, the slides were left for complete evaporation of DMSO (under vacuum) at room temperature for 2 hrs. After printing, the slides were exposed to UV irradiation 2.4 J/cm^2^ (365 nm) in a UV chamber (Amersham life science). The slides were subsequently washed with DMSO, ethanol, and finally with distilled water for 15 minutes (ultrasonically), respectively, to remove non-covalently bound compounds. After drying with nitrogen, the slides were assembled with flow cell and then mounted on SPRi instrument for measurement, or stored at 4°C to be used for later experiments.

##### SPRi method

All the experiments were carried out using the PlexArray® HT system (Plexera, LLC), which is based on surface plasmon resonance imaging. Oval regions of interests (ROIs) were set as 9 pixels × 7 pixels area in imaging area. ROIs of vehicle (DMSO) were used as controls for measurement of specific signals. All samples were injected with an association and dissociation flow rate of 3 μL s^−1^ and at 25°C. A 1:3 mixture of glycine. HCL (pH 4.2) and 0.5% (v/v) SDS, respectively, was used to regenerate the surface and remove bound proteins from the small molecules enabling the sensor chip to be reused for additional analyte injections.

##### Binding experiments and data analysis

The Tris buffer (10 mM Tris-HCl pH 8.0, 100 mM NaCl), without any reducing agent, was used as both analyte and running buffer. Purified recombinant proteins, including Skp1^WT^, ΔSkp1^1−140^, and any of the desired mutant(s), were in the Tris buffer (10 mM Tris-HCl pH 8.0, 100 mM NaCl). For testing the compound(s), multiple concentrations (0.5 μM, 1 μM, 4 μM and 8 μM) of Skp1^WT^ or ΔSkp1^1−140^ were flowed on the SPRi instrument as analyte to get accurate kinetic parameters. A typical sample injection cycle consists of 300 seconds association phase with the analyte solution and 400 seconds dissociation phase with running buffer at 3 μL s^−1^ flow rate. Other purified proteins such as mutant forms of Skp1 were tested to confirm binding pocket specificity. The highest concentration tested for each protein was 8 μM. For data analysis, we choose two software packages, ORIGIN Lab and Data Analysis Module (DAM) of Plexera. All data from SPRi reported here are after subtraction of the background intensity/signal by DAM software. In short, the entire concentration of analyte was fitted with a 1:1 Langmuir interaction integrated rate equation to obtain the kinetic constants.

#### Liquid chromatography-tandem mass spectrometry (LC-MS/MS) analysis of the *in vitro* binding between Skp1 and Z0933M

This assay accompanied site-directed mutagenesis and was performed to demonstrate the interaction between Skp1 and Z0933M. The Spk1^Mut^ plasmids (see key resources table) were generated based on the interacting residues predicted from our modeling studies of Z0933M and Skp1. Subsequently, Skp1^WT^, ΔSkp1^1−140^, all the Skp1^Mut^ plasmids, as well as the Skp1-F-box (fused) form were expressed and purified by following the same protocol as mentioned above. The proteins were quantified and incubated with Z0933M overnight. For this experiment, the stock solution of Z0933M was prepared in methyl hydroxide (MeOH). After a series of procedural steps (depicted in Figure S6A), the samples were prepared for (eluted protein(s) fractions) subsequent SDS-PAGE analysis (Figure S6B). By performing phase separation with an organic solvent ethyl-acetate (EA), the fractions of bound compound were obtained and then the identity of the bound compound was confirmed by LC-MS/MS analysis on an Orbitrap-Elite mass spectrometer (Thermo Scientific). From SDS-PAGE of protein(s) samples, the band intensities were detrmined. The peak heights from the extracted chromatograms for the bound compound(s) were analyzed and mass intensities were quantified. Finally, the resulting band- and mass-intensities were normalized to WT samples, and ratio(s) of mass intensity to band intensity were calculated. The resulting data was analyzed by using GraphPad Prism® v6.0 (San Diego, CA).

#### Cell viability assay

For cell viability, cells were seeded in 384 opaque-walled plates with clear bottoms at 300–500 cells per well (optimum density for growth) in a total volume of 20 μL of media. The media was according to culture conditions of each individual cell line (mentioned above). After 12 hrs, compounds were added in a total volume of 5 μL of media (two-fold diluted) to each well with final concentrations from 5 nM to 30 μM. The measurements were conducted 48 hrs after seeding of the cells. Then, 25 μL of Cell-Titer GLO reagent (Promega) was added, and luminescence was measured on an EnSpire multimode plate reader (PerkinElmer), according to the manufacturer’s instructions. The estimated *in vitro* half-maximal inhibitory concentration (EC_50_) values were calculated using GraphPad Prism® v6.0 (San Diego, CA) software. The cell viability for A549 cells was also performed for 72- and 96-hrs by using the same experimental conditions as described above.

Cell viability with inhibitor interventions was conducted in A549 cells for 48 hrs by following the same procedure as described above, except the interventional compounds were added 1 hr before the Z0933M. The information for relevant compounds is mentioned in the key resources table.

For cell viability with Skp1 WT and mutant(s) overexpression, A549 cells were seeded in 96 well opaque-walled plates with clear bottoms at 35,000–40,000 cells per well in a total volume of 100 μL of media. After 12 hrs, the media was replenished and the cells were transfected with pcDNA3.1-Skp1^WT or mutant^ plasmid(s) and pcDNA3.1 vector (control) for 24 hrs. Then, the media was removed, and 50 μL new media containing indicated concentrations (see results section) of Z0933M was added. After 48 hrs of incubation, 50 μL of Cell-Titer GLO reagent (Promega) was added, and luminescence was measured on an EnSpire multimode plate reader (PerkinElmer), according to the manufacturer’s instructions.

#### Cell-based Fluorescence Resonance Energy Transfer (FRET) assay

FRET occurs over short distances only (i.e., 2–10 nm) and, thus, represents a unique and powerful technique to monitor intermolecular protein interactions and target engagement in cells (Schaaf et al., 2017a, 2017b).

##### Development of Skp1-F-box PPI FRET

To develop an in-cell Skp1-F-box PPI assay, HEK293 cells were co-transfected with N-terminally mCherry-fused Skp1 (donor construct) and C-terminally copGFP-fused NIPA (acceptor construct) for 24 and 48 hrs (Figure S2B). To make sure that FRET is really happening, the cells were also transfected with a positive control (mCherry-copGFP fused) and a few negative controls (like unfused copGFP only, unfused mCherry, and unfused mCherry + copGFP). In addition, an empty vector transfection was used as a negative control to distinguish protein(s) expression in all systems. Microscopic immunofluorescence images revealed that protein expression in all systems was considerably enough at 48 hrs (Figure S2C). Next, we adopted the FRET system to 96-well format and, as shown in Figure S2C (bar graph), the average FRET fluorescence in our mCherry-Skp1 + NIPA-copGFP transfected system was comparable to that of the positive control. We further calculated the Z′ factor (as mentioned in the FP assay format) value from different batches of experiments (Z′ factor: 0.71), which suggested that our assay conditions were reliable to be adopted for compound(s) testing.

##### Compound testing

HEK293 cells at a density of 5 × 10^4^ cells/well in 100 μL of DMEM containing 10% FBS were seeded in white Greiner CellStar 96-well plates (Sigma). After 12 hrs of seeding, cells were co-transfected with 0.1 μg (100 ng) of the appropriate FRET constructs (mentioned in key resources table) (or an appropriate empty vector control) by Lipofectamine 2000 transfection according to the protocol of the manufacturer (LifeTech). The transfected cells were incubated at 37°C in a humidified incubator providing 5% CO_2_, and media was replenished after 8–12 hrs of transfection. After 48 hrs of transfection, if there is compound testing, the chemical compound was added into cell growth media, without phenol red, in increasing concentrations (like 1 μM, 15 μM, and 30 μM) followed by incubation for 4 hrs under normal cell-growth conditions. Otherwise, the medium was removed and replaced with PBS (1 PBS washing). Plates were then analyzed on a Perkin Elmer instrument in fluorescence mode. For copGFP, excitation (Ex) was set at 482 nm and reading emission (Em) at 502 nm. For mCherry, Ex was set at 587 nm and reading Em at 610 nm. Background fluorescence was determined from wells transfected with pCDNA3.1 vector and subtracted. The FRET ratio (FR) was calculated by following equation.

FR = FRET_c_ ‒ FRET_b_/FRET_GFPc_; Where FRET_c_ and FRET_GFPc_ are fluorescence intensities of the cells, and FRET_b_ is the background fluorescence intensity of the wells containing media or PBS. The resulting data was analyzed in GraphPad Prism® v6.0 (San Diego, CA). The potential utility of this assay for investigating the inhibitor interventions against Skp1-F-box PPIs was affirmed by using the known Skp1 inhibitor 6-OAP as a positive control (Figure S2E). The FRET ratio was calculated as a measure of compound’s ability to inhibit Skp1-F-box protein complex formation.

#### Co-immunoprecipitation (co-IP)

Co-IP experiments were performed according to a reported protocol (Fujimaki et al., 2001). Briefly, 1 × 10^7^ A549 cells were used for each experimental group (treated or untreated). The cells were washed using ice cold PBS, and subsequently harvested by using scrapper. The resulting suspension was centrifuged to remove the PBS and pellet the cells. The pellet was suspended in 600 μL volume of TNE lysis buffer (containing 10 mM Tris-HCl pH 7.4, 1% NP-40, 150 mM NaCl, 1 mM EDTA, 1 mM Na_3_VO_4_, 1 mM PMSF, and complete protease inhibitor cocktail). The resulting suspension was kept on ice for 20–30 min for complete cell lysis. Samples were homogenized using a syringe with a 0.4 mm diameter needle for 3–4 times. Resulting lysates were spun-down at 12000 xg for 10 min at 4°C. Then, the lysates were diluted with equal volume of TNE buffer and one-tenth of the supernatant was saved as protein input. For endogenous co-IP, the remaining lysate was divided into equal parts and incubated overnight at 4°C with 20 μL protein A/G Plus beads (Pierce Technology) per sample that were already pre-incubated with indicated antibodies at 4°C for 3 hrs. For flag-protein overexpressed samples, the lysates were incubated with 30 μL anti-FLAG magnetic M2 beads (Sigma-Aldrich) per sample at 4°C overnight. After immunoprecipitations, beads were washed five times with TBST (150 mM NaCl, 50 mM Tris-HCl pH 7.6, Tween 0.1%, and complete protease inhibitor cocktail) buffer to remove the non-specific bindings. Afterwards, the protein-protein complexes were eluted using elution buffer (20 mM Tris HCl pH 7.5, 2% SDS, 1 mM EDTA). The immunoprecipitates were then subjected to Western blotting. Detailed antibody information used for immunoblotting is mentioned in the key resources table.

#### Western blotting

For Western blotting of total protein(s), A549 cells were cultured in the absence or presence of Z0933M with various doses and at different time-points. The cells were harvested, lysed using RIPA buffer (Beyotime, China, P001B) in the presence of protease inhibitor cocktail (Roche), and quantified. The samples were loaded onto SDS-PAGE gel for separation, and transferred onto a polyvinylidene difluoride membrane (PVDF) or Nitrocellulose (NC) membrane(s) (Millipore). The membrane(s) was/were then blocked for at least 1 hr with 5% milk or Bovine Serum Albumin (BSA) in TBST (0.1% Tween-20 in Tris-buffered saline (TBS)), followed by incubation with the primary antibodies at 4°C overnight. Next, the membrane(s) was/were washed three times with TBST, incubated for 2 hrs at room temperature with HRP-conjugated secondary antibodies, and washed three times again with TBST. The signal was generated with ECL (details mentioned in key resources table) and detected with FUSION SOLO 4M System (Vilber Lourmat). Detailed antibody information is provided in key resources table.

#### Ubiquitination assay

For *in vivo* ubiquitination assay, A549 cells were cultured in the absence or presence of Z0933M with various doses and at different time-points. 6 hrs before harvest, the proteasome inhibitor, MG132 (10 μM), was added to accumulate polyubiquinated proteins and thus increase the sensitivity of detection. After treatment, the cells were directly lysed by the denatured buffer (8 M Urea, 300 mM NaCl, 0.5% NP40, 50 mM Tris-HCL pH 8.0, 1 mM PMSF, 1 mM Na_3_VO_4_, 1 mM NaF, complete protease inhibitor cocktail, and 10 mM N-ethylmaleimide (NEM), a deubiquitinating enzyme inhibitor). The lysates were normalized, and then subjected to Western blotting. Detailed antibody information used for detecting K48-linked polyubiquitylated chains is mentioned in the key resources table. For detecting ubiquitination of individual substrates, A549 cells were cultured in the absence or presence of Z0933M (1 μM and 2.5 μM doses) for 8 hrs, and treated with MG132 (10 μM) for 6 hrs before harvest. The total protein was extracted using IP Lysis Buffer containing 10 mM NEM. Ubiquitinated proteins in the cell lysate were collected by co-IP and then subjected to Western blotting.

#### Flow cytometric analysis of cell cycle

Both asynchronous and synchronous cells were analyzed by flow cytometry with DNA staining to determine the total amount of DNA. For asynchronous analysis, A549 cells were seeded into six-well plates at a density of 6 × 10^5^ cells/well. After 14 to 16 hrs of seeding, the cells were treated with indicated concentrations of Z0933M for 24 hrs. Then, the cells were harvested, washed twice with cold PBS, and fixed in cold 70% ethanol on ice (around 4°C) for 4 hrs. Next, the cells were washed twice with cold PBS, resuspended with cold PBS containing 20 mg/mL RnaseA (Sigma), and incubated for 30 min at 37°C. Then, PBS containing 50 mg/mL propidium iodide (PI) (Sigma) was directly added, the cells were mixed gently, and incubated for 5–10 min at room temperature in the dark. Finally, the cell-cycle phase distribution was analyzed in three different experiments using flow cytometry (BD Biosciences, San Diego, CA, USA). At least 10,000 events/sample were acquired. Assays were conducted three times independently. The data was analyzed by FlowJo V10 software. For synchronous cell cycle analysis, cells were synchronized to G1/S boundary by a double-thymidine blockade (Liu et al., 2015), and then exposed to Z0933M for time-dependent or dose-dependent analysis. Cells were harvested, fixed with 70% cold ethanol, incubated with RNase, and stained with PI. Cell cycle distribution was analyzed by flow cytometry (BD Biosciences, San Diego, CA, USA) and FlowJo V10 software.

#### Flow cytometric analysis of apoptosis

For flow cytometric analysis of apoptotic and necrotic cells, A549 cells were seeded in six-well plates at a density of 5 × 10^5^ cells/well and treated with vehicle (DMSO) or indicated concentrations of Z0933M for 12 and 24 hrs. The apoptotic rates were determined by an Annexin-V/PI assay kit (Beyotime Biotechnology, Nanjing, China). After incubation for indicated time intervals, cells were harvested, washed twice with a cold PBS, resuspended in 300-500 μL of annexin-V binding buffer, and then 10 μL of annexin-V–FITC and 5 μL of PI were added. After staining in the dark for 10–15 min at room temperature, the cells were analyzed in three independent experiments using flow cytometry (BD Biosciences, San Diego, CA, USA). The data was analyzed with FlowJo V10 software.

#### Transmission electron microscopy (TEM)

A549 cells were cultured in the RPMI medium supplemented with 10% FBS until they reach 80% confluency. Then the cells were treated at indicated concentrations of Z0933M for 24 hrs. Subsequently after treatment, cells were digested with 0.5% trypsin-EDTA, centrifuged, and pellet was washed once with 1 mL of PBS. Later, cells were pelleted by centrifugation for 3–5 min at 1000 rpm in 1.5 mL EP tube. After centrifugation supernatant was removed leaving the pellet that was fixed by adding 1 mL of fixation solution (4% formaldehyde and 1% glutaraldehyde in 0.1 M PB (pH 7.4)). While fixation cells were continuously mixed for 30 minutes at room temperature (or store cells at 4°C for long term storage). After fixation cells were washed 4 times with 0.1M PBS (pH 7.4) for 15 min each wash. After washing, second fixation was performed by adding 0.5 mL osmium tetra-oxide (1% in PBS) and cells were incubated for 2 hrs at room temperature. After osmic fixation, cells were washed 6 times by using 0.1M PBS (pH 7.4), 15 min each wash. Dehydration of cells was performed with 30%, 50%, 70%, 80%, 90%, and 100% alcohol subsequently for 5 min each while centrifugation (300 x g, 5 min) at each step. After dehydration cells were washed with Acetone twice, 5 min each wash. Then cells were incubated with Acetone: resin in 3:1 for 30 min and centrifugation was performed (300 x g, 5 min) to remove the solution. Second round of Acetone: resin incubation was performed by using 1:1 ratio for 2–4 hrs followed by centrifugation (300 x g, 5 min) to remove the solution. Third round of resin incubation was performed for overnight (12 hrs) at room temperature for complete penetration of resins into the cells. After resin penetration the samples embedding was performed by osmotic polymerization for 24 hrs at 40°C. After complete polymerization the sample were cut into 1–2 μm sections. We performed the ultrathin sectioning to have a clear staining and imaging. Uranyl acetate and alkaline were used for staining the sections and subsequently imaging was performed.

#### Measurement of caspase(s) activity

The activities of caspase-3/7, −8 and −9 were measured using Caspase-Glo® 3/7,8, and 9 assay kits (Promega, Madison, WI, USA) according to the manufacturer’s protocol. Briefly, the cells were plated over-night in a white 384 well microplate, and then treated as indicated with Z0933M. After treatment, 25 μL of each caspase substrate was added and incubated at room temperature for 30 min. Upon activation of caspase in the apoptotic cells, the substrate will give a luminescence signal after cleavage of the aminoluciferin-labelled synthetic tetrapeptide. Luminescent signals were detected using an EnSpire multimode plate reader (PerkinElmer). For inhibitor interventions and Skp1 overexpression, the experiments were performed in 96 well format.

#### Nuclear and cytoplasmic fractionation

Nuclear and cytoplasmic fractionation was perfumed using Nuclear and Cytoplasmic Protein Extraction Kit (Key GEN Biotech) and according to manufacturer’s protocol.

#### Real-time quantitative polymerase chain reaction (RT-qPCR)

Total cells were extracted by using Trizol reagents as described by the manufacturer (Invitrogen). 2 μg of RNA was reverse transcribed into single stranded cDNA with a final volume of 20 μL of reaction buffer using a reverse transcription kit (TOYOBO CO., LTD). The mRNA expression(s) of targeted gene(s) was/were quantified by using LightCycler® FastStart DNA Master SYBR Green I (Roche). The experiments were performed on Bio-Rad real-time PCR machine. Data were analyzed based on 2ˆΔΔCt method. Samples were run in triplicates and data were normalized based on the expression level of *β-actin* (housekeeping gene). The primers used in this study are mentioned in Table S1.

#### Immunofluorescence (IF) staining

For IF staining, the cells were cultured on 0.1% Geltrex-coated glass coverslips in six-well plates. After Z0933M treatment at indicated doses and time-interval, the cells were washed with PBS (Gibco) and fixed in 4% paraformaldehyde at room temperature for 30–60 min. The cells were then permeabilized and blocked with blocking solution (PBS, 3% BSA (Invitrogen), and 0.2% Triton X-100) at room temperature for 30–60 min. Primary antibodies were diluted in blocking solution and added to the cells at room temperature for 1 hr and at 4°C overnight. After three washing steps with PBS, the coverslips were incubated in appropriate secondary antibodies prepared in blocking solution for 2 hrs at room temperature. Then, cells were washed with PBS and counterstained with a 1 μg mL^−1^ 4′,6-diamidino-2-phenylindole (DAPI) solution (Sigma) for 5 min at room temperature. Finally, the coverslips were mounted on slides with 80% glycerol and images were obtained by confocal microscope.

#### Induced-Fit docking (IFD)

For IFD, Schrodinger’s IFD protocol (Schrödinger, LLC, New York, NY, 2017-1) was followed. Briefly, protein (Skp1) was prepared using the Protein Preparation Wizard (Schrödinger Suite, LLC, New York, NY, 2017-1). H-bonds were optimized, and restrained minimization was performed to relax the structure. Grid was then generated using the receptor grid generation tools. The grid box was set directly on the P1 region of Skp1, with an enclosing box of 20 Å covering the P1 residues, and other settings left as default. The compound was prepared using LigPrep (LigPrep, Schrödinger, LLC, New York, NY, 2017-1). The analyses of ligand-protein interaction(s) were performed using graphical interface of Maestro (Schrödinger, Mannheim, Germany) and PyMOL version 1.8.8.2.

#### Molecular dynamics (MD) simulations

We have used the available X-ray structure of Skp1 (PDB: 2AST) for IFD. However, in the X-ray structure of Skp1 protein, residues 37–42 and 69–84 were missing. This makes the X-ray structure of Skp1 unfit for our molecular dynamics (MD) simulations. Therefore, to solve this problem, we used the *H. sapiens* Skp1 sequence (Uniprot ID: P63208) and the Skp1 X-ray structure from 2AST (Chain B) as templates to generate the full-length Skp1 structure using the Prime module in (Schrödinger, LLC, New York, NY, 2017-1). For MD simulations, the initial orientation of the Z0933M was determined from the IFD pose. The Amber ff14SB force field and the general Amber force field (GAFF) (Wang et al., 2004) were used for protein (homology model of Skp1) and ligands, respectively. Then, complex structures were solvated using a cubic box of TIP3P water molecules (Price and Brooks, 2004), which were extended at least 10 Å away from the boundary of any protein atoms. An appropriate number of Na^+^ and Cl^−^ ions were added to neutralize the system. Each system was then submitted to energy minimization. First, water molecules, hydrogen atoms, and salt ions were subjected to 2500 steps of steepest descent minimization and 2500 steps of conjugate gradient minimization, whereas other heavy atoms were constrained with the harmonic force of 50 kcal mol^−1^ Å^−2^. Next, the heavy atoms were constrained with a harmonic force of 25 kcal mol^−1^ Å^−2^ and minimized with 2500 steps of steepest descent minimization and 2500 steps of conjugate gradient. Then, the system was heated from 0 to 300 K and equilibrated for 500 ps. Finally, 200 ns long simulations without restriction were conducted for ligand-protein complex at a constant pressure and temperature (T = 300 K, P= 1 atm). During MD simulations, the SHAKE method was applied to constrain the covalent bonds involving the hydrogen atom (Ruymgaart and Elber, 2012). The Particle Mesh Ewald (PME) method was adopted to treat the long-range electrostatic interaction (Darden et al., 1993). The cutoff distances for the long-range electrostatic and van der Waals interactions were set to 10 Å.

#### MD trajectory analysis and per residue energy decomposition

Root mean square deviation (RMSD) of the Cα was calculated from the MD trajectories at 100 ps intervals. Clustering is a means of partitioning data so that data points inside a cluster are more similar to each other than they are to points outside a cluster. The cluster analysis (Shao et al., 2007) of protein conformations as well as the measurement of distance of distance(s) between C-α atoms was carried out using cpptraj module. PyMOL version 1.8.8.2 was used for structural alignments and visualizations. For plotting graphs, MS Excel (2010) and GraphPad Prism® v6.0 (San Diego, CA) were used. To obtain a detailed view of the protein-ligand binding and identify the key residues responsible for the binding, free energy decomposition to each residue was performed for the last 10 ns snapshots while using the Molecular Mechanics/Polarized Born Surface Area (MM/PBSA) method (Genheden and Ryde, 2015).

### Quantification and statistical analysis

Data are expressed as mean ± SD of two or three replicates, as indicated in figure legends, and represented using GraphPad Prism. Comparisons of multiple groups were performed using 1-way ANOVA. Comparisons between 2 groups were performed using the student’s t test. All statistical calculations are included in the relevant figure legends. p values less than 0.05 were accepted as significant, ∗p < 0.05, ∗∗p < 0.01, ∗∗∗p < 0.001. Dose response curve and IC_50_ values are generated by non-linear regression for parameters fitting curves using GraphPad Prism.

## Data Availability

The data reported in this paper will be shared by the lead contact, Jinsong Liu (liu_jinsong@gibh.ac.cn). This paper does not report original code. Any additional information required to reanalyze the data reported in this paper is available from the lead contact upon request.

## References

[bib1] Ai T.J., Sun J.Y., Du L.J., Shi C., Li C., Sun X.N., Liu Y., Li L., Xia Z., Jia L. (2018). Inhibition of neddylation by MLN4924 improves neointimal hyperplasia and promotes apoptosis of vascular smooth muscle cells through p53 and p62. Cell Death Differ..

[bib2] Arkin M.R., Tang Y., Wells J.A. (2014). Small-molecule inhibitors of protein-protein interactions: progressing toward the reality. Chem. Biol..

[bib3] Bouchard V.J., Rouleau M., Poirier G.G. (2003). PARP-1, a determinant of cell survival in response to DNA damage. Exp. Hematol..

[bib4] Chandra Dantu S., Nathubhai Kachariya N., Kumar A. (2016). Molecular dynamics simulations elucidate the mode of protein recognition by Skp1 and the F-box domain in the SCF complex. Proteins.

[bib5] Chen P., Hu T., Liang Y., Li P., Chen X., Zhang J., Ma Y., Hao Q., Wang J., Zhang P. (2016). Neddylation inhibition activates the extrinsic apoptosis pathway through ATF4-CHOP-DR5 Axis in human esophageal cancer cells. Clin. Cancer Res..

[bib6] Cheng X., Liu Y.Q., Wang G.Z., Yang L.N., Lu Y.Z., Li X.C., Zhou B., Qu L.W., Wang X.L., Cheng Y.X. (2017). Proteomic identification of the oncoprotein STAT3 as a target of a novel Skp1 inhibitor. Oncotarget.

[bib7] Cohen I., Zhao D., Bar C., Valdes V.J., Dauber-Decker K.L., Nguyen M.B., Nakayama M., Rendl M., Bickmore W.A., Koseki H. (2018). PRC1 fine-tunes gene repression and activation to safeguard skin development and stem cell specification. Cell Stem Cell.

[bib8] Cui D., Xiong X., Shu J., Dai X., Sun Y., Zhao Y. (2020). FBXW7 confers radiation survival by targeting p53 for degradation. Cell Rep..

[bib9] Darden T., York D., Pedersen L. (1993). Particle mesh Ewald: an N⋅log(N) method for Ewald sums in large systems. J. Chem. Phys..

[bib10] Ferris J., Espona-Fiedler M., Hamilton C., Holohan C., Crawford N., McIntyre A.J., Roberts J.Z., Wappett M., McDade S.S., Longley D.B. (2020). Pevonedistat (MLN4924): mechanism of cell death induction and therapeutic potential in colorectal cancer. Cell Death Discov..

[bib11] Fujimaki W., Iwashima M., Yagi J., Zhang H., Yagi H., Seo K., Imai Y., Imanishi K., Uchiyama T. (2001). Functional uncoupling of T-cell receptor engagement and Lck activation in anergic human thymic CD4+ T cells. J. Biol. Chem..

[bib12] Genheden S., Ryde U. (2015). The MM/PBSA and MM/GBSA methods to estimate ligand-binding affinities. Expert Opin. Drug Discov..

[bib13] Hill S., Reichermeier K., Scott D.C., Samentar L., Coulombe-Huntington J., Izzi L., Tang X., Ibarra R., Bertomeu T., Moradian A. (2019). Robust cullin-RING ligase function is established by a multiplicity of poly-ubiquitylation pathways. Elife.

[bib14] Hussain M., Lu Y., Liu Y.Q., Su K., Zhang J., Liu J., Zhou G.B. (2016). Skp1: implications in cancer and SCF-oriented anti-cancer drug discovery. Pharmacol. Res..

[bib15] Jia L., Li H., Sun Y. (2011). Induction of p21-dependent senescence by an NAE inhibitor, MLN4924, as a mechanism of growth suppression. Neoplasia.

[bib16] Jiang W., Crowe J.L., Liu X., Nakajima S., Wang Y., Li C., Lee B.J., Dubois R.L., Liu C., Yu X. (2015). Differential phosphorylation of DNA-PKcs regulates the interplay between end-processing and end-ligation during nonhomologous end-joining. Mol. Cell.

[bib17] Jin J., Ang X.L., Shirogane T., Wade Harper J. (2005). Identification of substrates for F-box proteins. Methods Enzymol..

[bib18] Kitagawa K., Hiramatsu Y., Uchida C., Isobe T., Hattori T., Oda T., Shibata K., Nakamura S., Kikuchi A., Kitagawa M. (2009). Fbw7 promotes ubiquitin-dependent degradation of c-Myb: involvement of GSK3-mediated phosphorylation of Thr-572 in mouse c-Myb. Oncogene.

[bib19] Kubota R., Hamachi I. (2015). Protein recognition using synthetic small-molecular binders toward optical protein sensing in vitro and in live cells. Chem. Soc. Rev..

[bib20] Lea W.A., Simeonov A. (2011). Fluorescence polarization assays in small molecule screening. Expert Opin. Drug Discov..

[bib21] Lees A., McIntyre A.J., Crawford N.T., Falcone F., McCann C., Holohan C., Quinn G.P., Roberts J.Z., Sessler T., Gallagher P.F. (2020). The pseudo-caspase FLIP(L) regulates cell fate following p53 activation. Proc. Natl. Acad. Sci. USA.

[bib22] Lepage C.C., Palmer M.C.L., Farrell A.C., Neudorf N.M., Lichtensztejn Z., Nachtigal M.W., McManus K.J. (2021). Reduced SKP1 and CUL1 expression underlies increases in Cyclin E1 and chromosome instability in cellular precursors of high-grade serous ovarian cancer. Br. J. Cancer.

[bib23] Lin D.I., Barbash O., Kumar K.S., Weber J.D., Harper J.W., Klein-Szanto A.J., Rustgi A., Fuchs S.Y., Diehl J.A. (2006). Phosphorylation-dependent ubiquitination of cyclin D1 by the SCFFBX4-αB crystallin complex. Mol. Cell.

[bib24] Lin J.J., Milhollen M.A., Smith P.G., Narayanan U., Dutta A. (2010). NEDD8-targeting drug MLN4924 elicits DNA rereplication by stabilizing Cdt1 in S phase, triggering checkpoint activation, apoptosis, and senescence in cancer cells. Cancer Res..

[bib25] Liu D., Zhang N., Du J., Cai X., Zhu M., Jin C., Dou Z., Feng C., Yang Y., Liu L. (2006). Interaction of Skp1 with CENP-E at the midbody is essential for cytokinesis. Biochem. Biophys. Res. Commun..

[bib26] Liu Y.Q., Wang X.L., Cheng X., Lu Y.Z., Wang G.Z., Li X.C., Zhang J., Wen Z.S., Huang Z.L., Gao Q.L. (2015). Skp1 in lung cancer: clinical significance and therapeutic efficacy of its small molecule inhibitors. Oncotarget.

[bib27] Luo Z., Yu G., Lee H.W., Li L., Wang L., Yang D., Pan Y., Ding C., Qian J., Wu L. (2012). The Nedd8-activating enzyme inhibitor MLN4924 induces autophagy and apoptosis to suppress liver cancer cell growth. Cancer Res..

[bib28] Makafe G.G., Hussain M., Surineni G., Tan Y., Wong N.K., Julius M., Liu L., Gift C., Jiang H., Tang Y. (2019). Quinoline derivatives kill Mycobacterium tuberculosis by activating glutamate kinase. Cell Chem. Biol..

[bib29] Meng X., Liu X., Guo X., Jiang S., Chen T., Hu Z., Liu H., Bai Y., Xue M., Hu R. (2018). FBXO38 mediates PD-1 ubiquitination and regulates anti-tumour immunity of T cells. Nature.

[bib30] Nikolovska-Coleska Z., Wang R., Fang X., Pan H., Tomita Y., Li P., Roller P.P., Krajewski K., Saito N.G., Stuckey J.A. (2004). Development and optimization of a binding assay for the XIAP BIR3 domain using fluorescence polarization. Anal. Biochem..

[bib31] Piva R., Liu J., Chiarle R., Podda A., Pagano M., Inghirami G. (2002). In vivo interference with Skp1 function leads to genetic instability and neoplastic transformation. Mol. Cell Biol..

[bib32] Price D.J., Brooks C.L. (2004). A modified TIP3P water potential for simulation with Ewald summation. J. Chem. Phys..

[bib33] Qvarnstrom O.F., Simonsson M., Eriksson V., Turesson I., Carlsson J. (2009). gammaH2AX and cleaved PARP-1 as apoptotic markers in irradiated breast cancer BT474 cellular spheroids. Int. J. Oncol..

[bib34] Reed S.M., Quelle D.E. (2014). p53 acetylation: regulation and consequences. Cancers.

[bib35] Reitsma J.M., Liu X., Reichermeier K.M., Moradian A., Sweredoski M.J., Hess S., Deshaies R.J. (2017). Composition and regulation of the cellular repertoire of SCF ubiquitin ligases. Cell.

[bib36] Ruymgaart A.P., Elber R. (2012). Revisiting molecular dynamics on a CPU/GPU system: water kernel and SHAKE parallelization. J. Chem. Theor. Comput..

[bib37] Schaaf T.M., Peterson K.C., Grant B.D., Bawaskar P., Yuen S., Li J., Muretta J.M., Gillispie G.D., Thomas D.D. (2017). High-throughput spectral and lifetime-based FRET screening in living cells to identify small-molecule effectors of SERCA. SLAS Discov..

[bib38] Schaaf T.M., Peterson K.C., Grant B.D., Thomas D.D., Gillispie G.D. (2017). Spectral unmixing plate reader: high-throughput, high-precision FRET assays in living cells. SLAS Discov..

[bib39] Seo B.R., Min K.J., Woo S.M., Choe M., Choi K.S., Lee Y.K., Yoon G., Kwon T.K. (2017). Inhibition of cathepsin S induces mitochondrial ROS that sensitizes TRAIL-mediated apoptosis through p53-mediated downregulation of Bcl-2 and c-FLIP. Antioxid. Redox Signal..

[bib40] Serrano M.A., Li Z., Dangeti M., Musich P.R., Patrick S., Roginskaya M., Cartwright B., Zou Y. (2013). DNA-PK, ATM and ATR collaboratively regulate p53-RPA interaction to facilitate homologous recombination DNA repair. Oncogene.

[bib41] Shao J., Tanner S.W., Thompson N., Cheatham T.E. (2007). Clustering molecular dynamics trajectories: 1. Characterizing the performance of different clustering algorithms. J. Chem. Theor. Comput..

[bib42] Shiba-Ishii A., Hong J., Hirokawa T., Kim Y., Nakagawa T., Sakashita S., Sakamoto N., Kozuma Y., Sato Y., Noguchi M. (2019). Stratifin inhibits SCF(FBW7) formation and blocks ubiquitination of oncoproteins during the course of lung adenocarcinogenesis. Clin. Cancer Res..

[bib43] Soucy T.A., Smith P.G., Milhollen M.A., Berger A.J., Gavin J.M., Adhikari S., Brownell J.E., Burke K.E., Cardin D.P., Critchley S. (2009). An inhibitor of NEDD8-activating enzyme as a new approach to treat cancer. Nature.

[bib44] Tang X., Orlicky S., Liu Q., Willems A., Sicheri F., Tyers M. (2005). Genome-wide surveys for phosphorylation-dependent substrates of SCF ubiquitin ligases. Methods Enzymol..

[bib45] Thompson L.L., Baergen A.K., Lichtensztejn Z., McManus K.J. (2020). Reduced SKP1 expression induces chromosome instability through aberrant cyclin E1 protein turnover. Cancers.

[bib46] Tian C., Lang T., Qiu J., Han K., Zhou L., Min D., Zhang Z., Qi D. (2020). SKP1 promotes YAP-mediated colorectal cancer stemness via suppressing RASSF1. Cancer Cell Int..

[bib47] Vidal M., Starowicz K. (2017). Polycomb complexes PRC1 and their function in hematopoiesis. Exp. Hematol..

[bib48] Wang J., Wolf R.M., Caldwell J.W., Kollman P.A., Case D.A. (2004). Development and testing of a general amber force field. J. Comput. Chem..

[bib49] Wang K., Deshaies R.J., Liu X. (2020). Assembly and regulation of CRL ubiquitin ligases. Adv. Exp. Med. Biol..

[bib50] Wang Z., Gearhart M.D., Lee Y.W., Kumar I., Ramazanov B., Zhang Y., Hernandez C., Lu A.Y., Neuenkirchen N., Deng J. (2018). A non-canonical BCOR-PRC1.1 complex represses differentiation programs in human ESCs. Cell Stem Cell.

[bib51] Wong S.J., Gearhart M.D., Taylor A.B., Nanyes D.R., Ha D.J., Robinson A.K., Artigas J.A., Lee O.J., Demeler B., Hart P.J. (2016). KDM2B recruitment of the Polycomb group complex, PRC1.1, requires cooperation between PCGF1 and BCORL1. Structure.

[bib52] Wu S., Yang J., Xu H., Wang X., Zhang R., Lu W., Yang J., Li X., Chen S., Zou Y. (2021). Circular RNA circGLIS3 promotes bladder cancer proliferation via the miR-1273f/SKP1/Cyclin D1 axis. Cell Biol. Toxicol..

[bib53] Xie J., Jin Y., Wang G. (2019). The role of SCF ubiquitin-ligase complex at the beginning of life. Reprod. Biol. Endocrinol..

[bib54] Zhang J.H., Chung T.D., Oldenburg K.R. (1999). A simple statistical parameter for use in evaluation and validation of high throughput screening assays. J. Biomol. Screen.

[bib55] Zheng H., Shen M., Zha Y.L., Li W., Wei Y., Blanco M.A., Ren G., Zhou T., Storz P., Wang H.Y. (2014). PKD1 phosphorylation-dependent degradation of SNAIL by SCF-FBXO11 regulates epithelial-mesenchymal transition and metastasis. Cancer Cell.

[bib56] Zhu H., Zhang H., Pei Y., Liao Z., Liu F., Su C., Liu Y., Dong R., Song J., Zhang X. (2021). Long non-coding RNA CCDC183-AS1 acts AS a miR-589-5p sponge to promote the progression of hepatocellular carcinoma through regulating SKP1 expression. J. Exp. Clin. Cancer Res..

