# Supplementary material for: A small-molecule Skp1 inhibitor elicits cell death by p53-dependent mechanism
Source: iScience. 2022 Jun 14;25(7):104591. doi: 10.1016/j.isci.2022.104591 (PMC9249674; doi:10.1016/j.isci.2022.104591)
Supplement: Document S1. Figures S1–S9, Table S1 and Data S1 [file mmc1.pdf]

iScience, Volume *25*

Supplemental information

A small-molecule Skp1 inhibitor

elicits cell death by p53-dependent mechanism

Muzammal Hussain, Yongzhi Lu, Muqddas Tariq, Hao Jiang, Yahai Shu, Shuang Luo, Qiang Zhu, Jiancun Zhang, and Jinsong Liu

Supplementary information

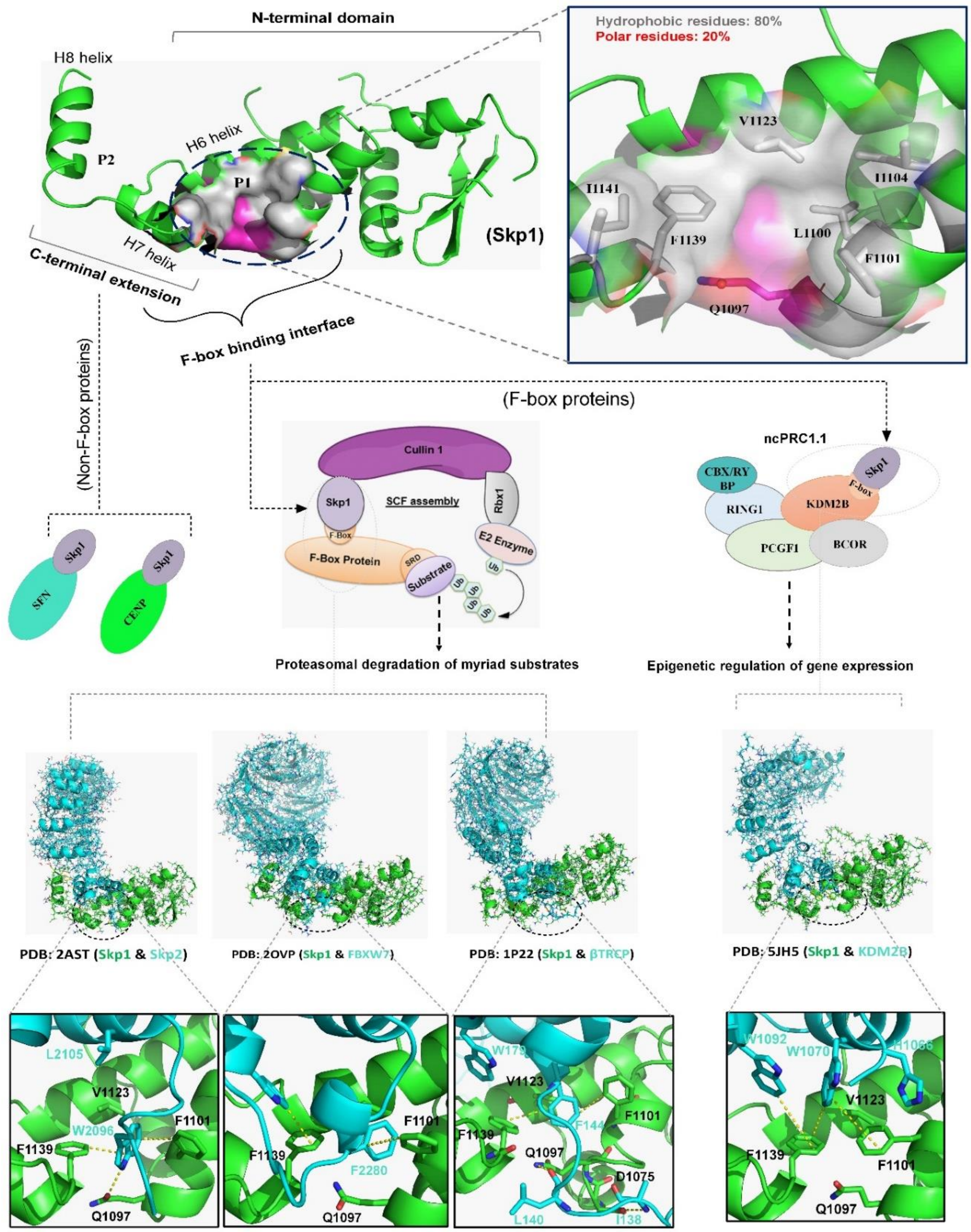

Figure S1. A general layout of Skp1 partnership in the molecular machinery of the cell in context of its structural aspects, related to text in the Introduction, and Figure 1.

The inset panel in the top-right corner highlights the key residues, including the aromatic cage phenylalanines (F101 and F139), constituting the hydrophobic hot spot region alongside H7 helix of C-terminal extension. The inset panels at the bottom highlight the interaction(s) mediated by key residues of hydrophobic hotspot region in context of crystal structure complexes of Skp1 with F-box proteins. The π-π interactions mediated by aromatic cage phenylalanines of Skp1 can be clearly observed.

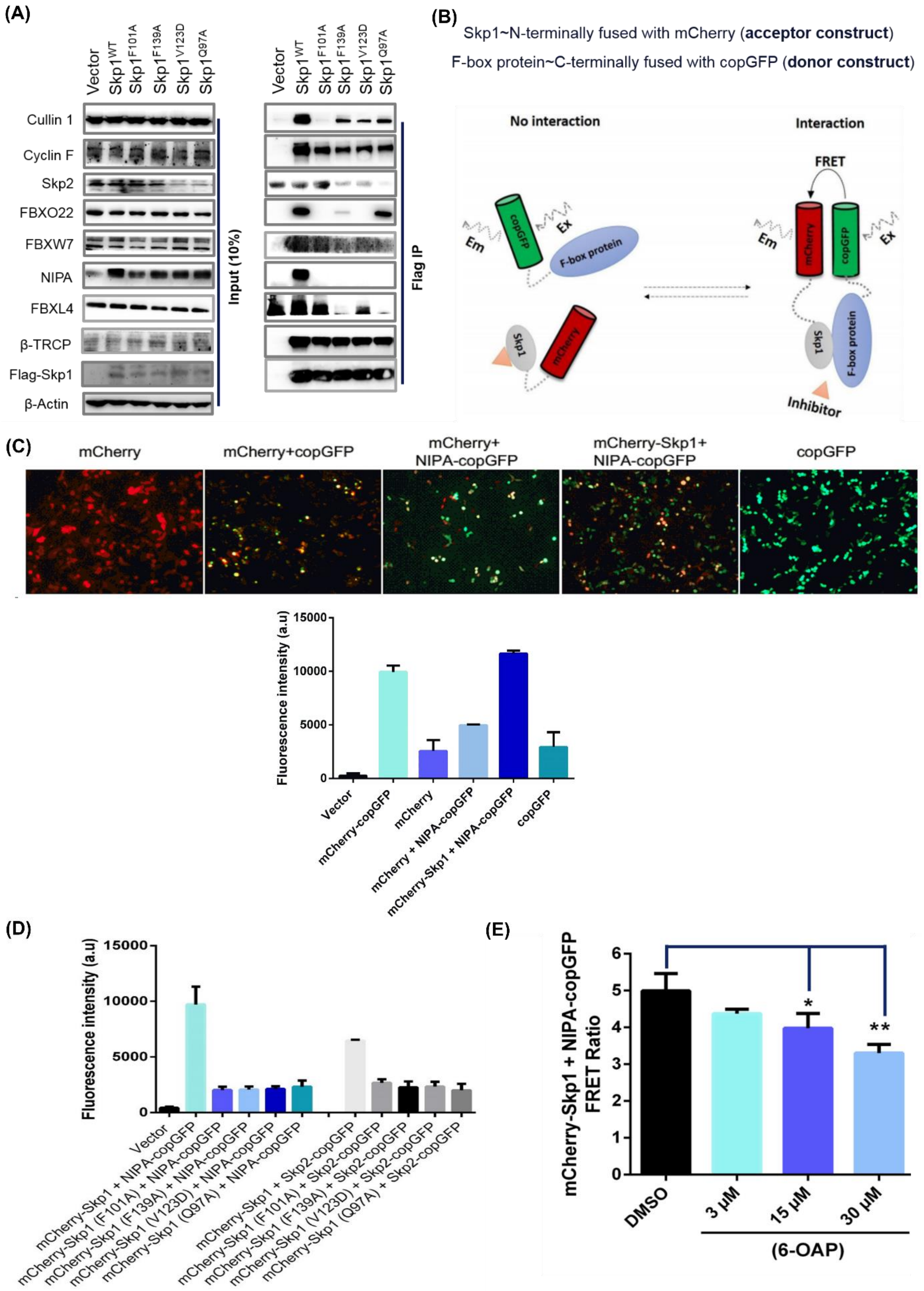

Figure S2. Flag-IP and cell-based FRET assays with Skp1 WT and mutant(s) plasmids, related to the text in Results "**Biochemical identification of Z0933M as a potent inhibitor of Skp1-F-box PPIs**", and Figures 1 and 3.

(A) Flag-IP experiment conducted from cellular lysates of A549 cells that were transfected with Skp1 WT and mutant(s) plasmids. The results show disruption of Skp1-F-box PPIs after site-directed mutagenesis of aromatic cage phenylalanines (F101 and F139) and the key residues in vicinity (Q97 and V123).
(B) Design strategy and working principle of cell-based FRET. Skp1 was N-terminally fused with mCherry (donor construct), while F-box protein(s) was/were fused with copGFP (acceptor construct) at the C-terminal. The Skp1-F-box PPIs would bring mCherry and copGFP in close proximity, leading to FRET occurrence upon excitation (Ex) and emission (Em) at specific wavelengths. Inhibitor intervention(s) against Skp1-F-box PPIs would prevent FRET occurrence because of dis-oriented spatial location(s) of mCherry and cop-GFP.
(C) Microscopic immunofluorescence images showing successful expression of proteins for various constructs (plasmids) related to cell-based FRET assay. The lower bar graph highlights the fluorescence intensity for various constructs of cell-based FRET assay after successfully adopted to 96-well plate format. The average FRET fluorescence in our mCherry-Skp1 + NIPA-copGFP transfected system was comparable to that of the positive control (mCherry-copGFP fused).
(D) Cell-based FRET assay (96-well plate format) demonstrating disruption of Skp1-F-box PPIs after site-directed mutagenesis of aromatic cage phenylalanines (F101 and F139) and the key residues in vicinity (Q97 and V123).
(E) The positive control ligand (6-OAP) caused dose-dependent disruption of Skp1-NIPA PPIs in a 96-well format cell-based FRET assay. Two-way ANOVA was performed to determine statistical significance (*$p \leq 0.05$; **$p \leq 0.01$).

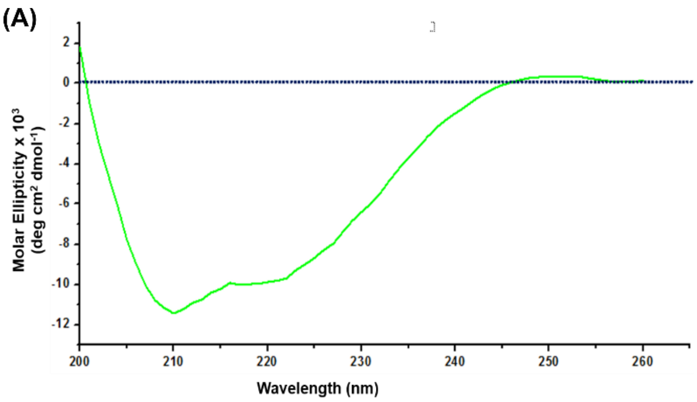

(B)

Secondary structure	pH 8.5 (%)	pH 8 (%)
Helix	90.20	87.30
Antiparallel	0.40	1
Parallel	0.70	0.70
Beta-Turn	8.30	9.50
Random Coil	1.90	1.40

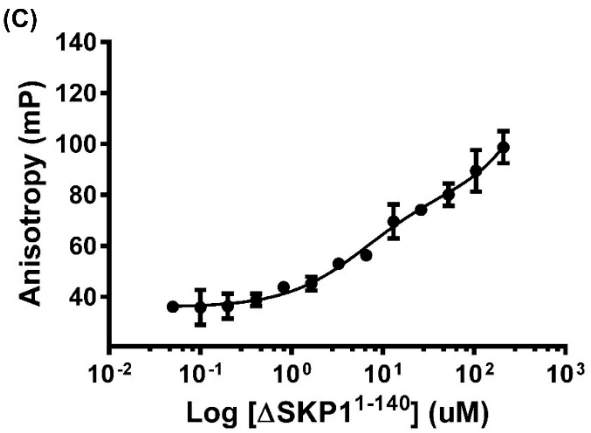

Figure S3. Development and characterization of fluorescence polarization (FP)-based *in vitro* competition assay, related to Figure 1.

(A) Representative CD spectra for the synthesized tracer, indicating that our F-box-based peptide construction could fold and attain a certain conformation to potentially interact with Skp1.

(B) Secondary structure components of the synthesized F-box peptide determined at two different pH buffer conditions.

(C) The titration curve of the recombinant purified $\Delta Skp1^{1\text{-}140}$, and the F-box tracer. The curve did not reach a plateau even by using a concentration of $\Delta Skp1^{1\text{-}140}$ two fold than that of $Skp1^{WT}$, inferring that the F-box peptide specifically interacts with the F-box binding interface of Skp1.

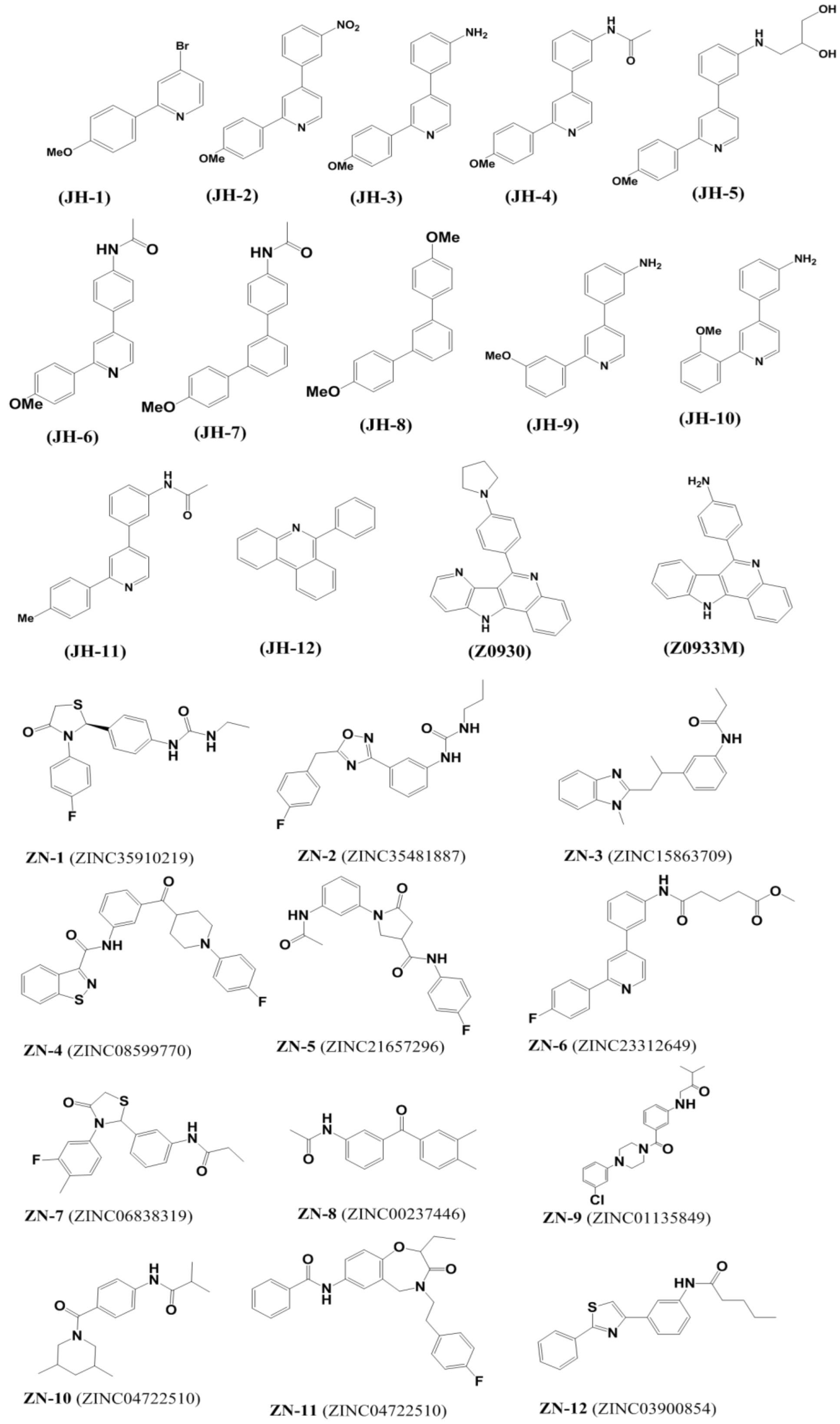

Figure S4. Chemical structures of the compounds tested in this study, Related to Figure 1 and text in STAR Methods.

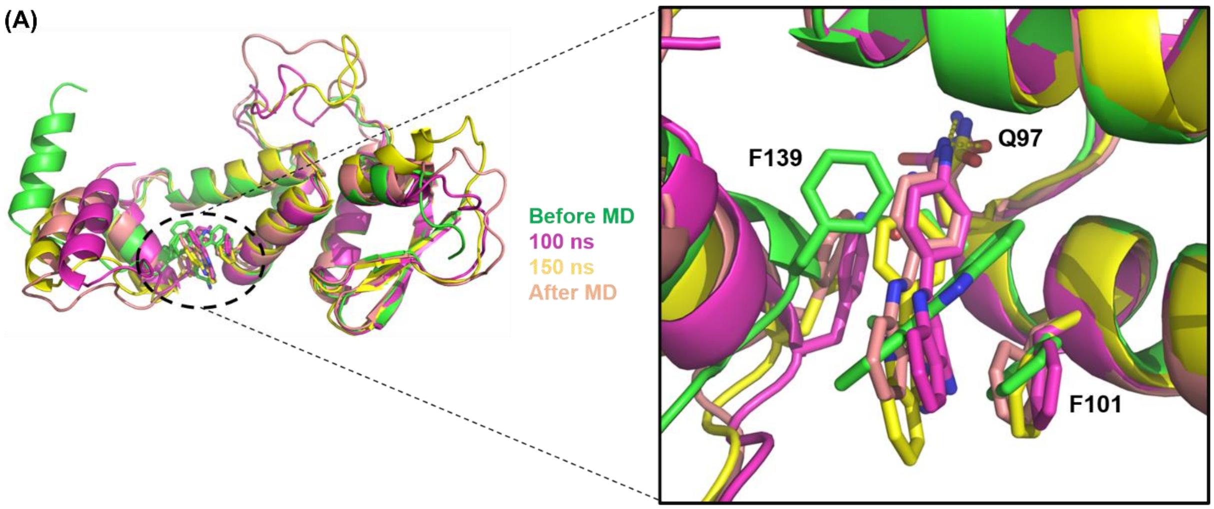

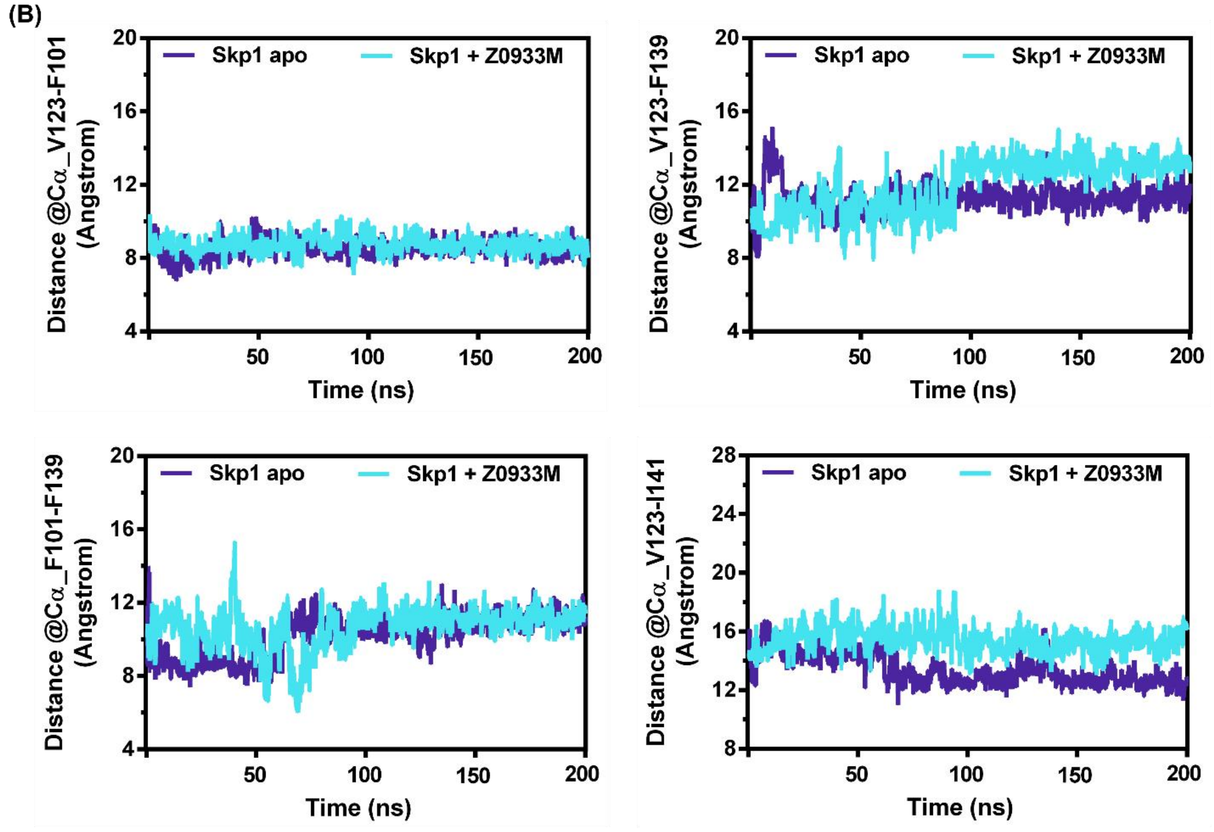

Figure S5. *In silico* assessment of Z0933M interaction with P1 hotspot of Skp1 using molecular dynamics (MD) simulations, related to Figure 2.

(A) An overlap of the structural snapshots from initial IFD (referred as "Before MD") and the ones extracted at 100 ns, 150 ns, and 200 ns (referred as "After MD") of the MD trajectory. The MD snapshots highlight the insertion of aniline moiety deep in the pocket, thus coordinating an H-bond interaction with Q97. The quinoline core and 1H-indol-3-yl moiety could still be predicted to coordinate intramolecular face-to-face (or edge-to-face) dual stacking interactions with the aromatic cage F101 and F139 residues. This highlights the stability of dually-stacked Z0933M within the aromatic cage, as predicted by our initial IFD simulations.

(B) The distance maps between the Cα atoms of the P1 residues that are critical for mediating interaction with Z0933M.

(A)

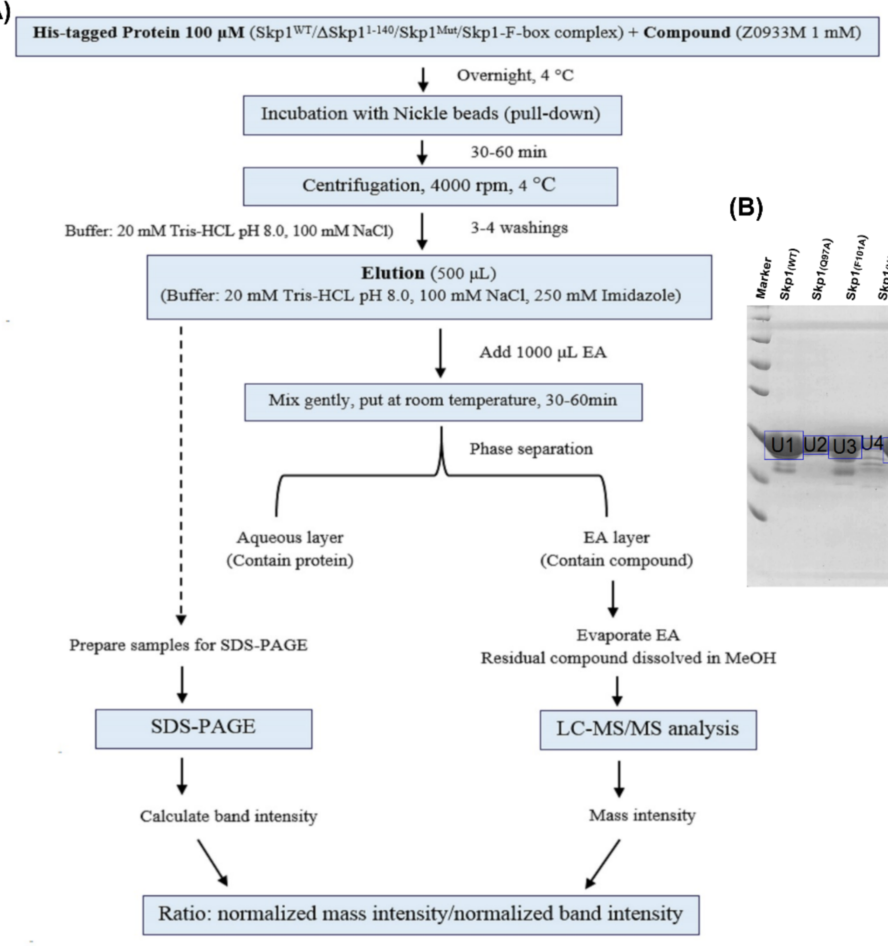

(B)

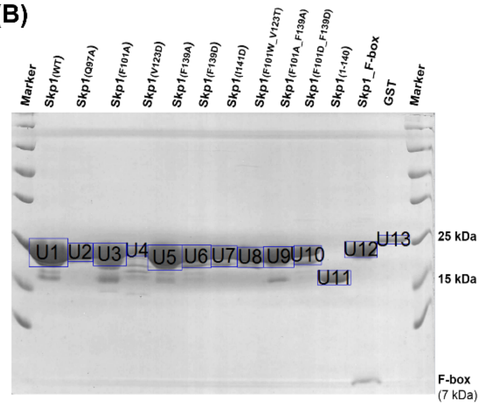

(C)

	Batch 1			Batch 2			Mean ± SEM
Z0933M + Protein	Mass intensity (MI)	SDS PAGE band intensity (BI)	Ratio (normalized MI/BI)	Mass intensity (MI)	SDS PAGE band intensity (BI)	Ratio (normalized MI/BI)	
Skp1(WT)	1370000	3348.963049870	1	173610	6839	1	1
Skp1(F101A)	719860	2901.37789	0.606504	124170	4626	1.057493	0.83 ± 0.15
Skp1(F139A)	350410	3589.69197	0.238621	85116	5427	0.617797	0.42 ± 0.13
Skp1(1-140)	655130	2148.72523	0.745309	124140	2551	1.917259	1.33 ± 0.41
Skp1_F-box	889580	2136.96181	1.017602	137380	2947	1.836657	1.42 ± 0.28
GST	000	1362	000				000

Figure S6. LC-MS analysis of the Z0933M binding with Skp1^{WT}, ΔSkp1$^{1-140}$, Skp1-F-box, and P1 mutants of Skp1, related to Figure 2.

(A) Schematics of the LC-MS/MS analysis of the *in vitro* binding between Skp1 and Z0933M.

(B) The SDS-PAGE of the samples prepared from eluted samples of incubated proteins with Z0933M and GST (negative control).

(C) Tabular representation of the recorded mass and SDS PAGE band intensities for two independent batch of experiments. The ratio between normalized mass intensity (from LC-MS spectra) and the normalized band intensity (from SDS-PAGE) was calculated for each sample.

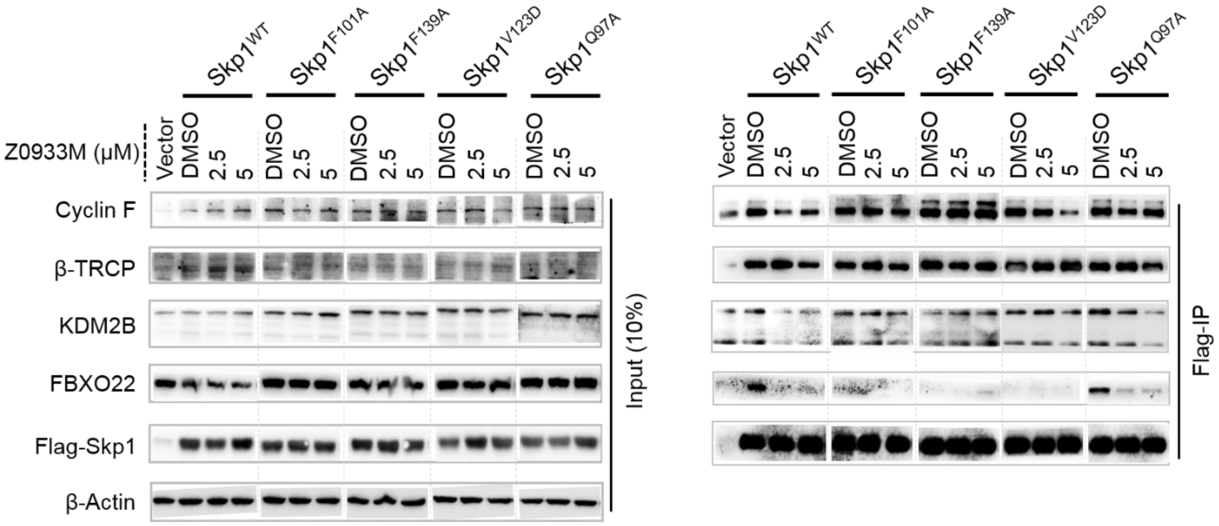

Figure S7. Flag-IP experiment with Skp1^{WT} and mutant(s) plasmids validating the Z0933M interaction with P1 residues, related to Figure 3.
Z0933M could not disrupt Skp1-F-box PPIs particularly in case of Flag-*Skp1^{F139A}* transfected samples.

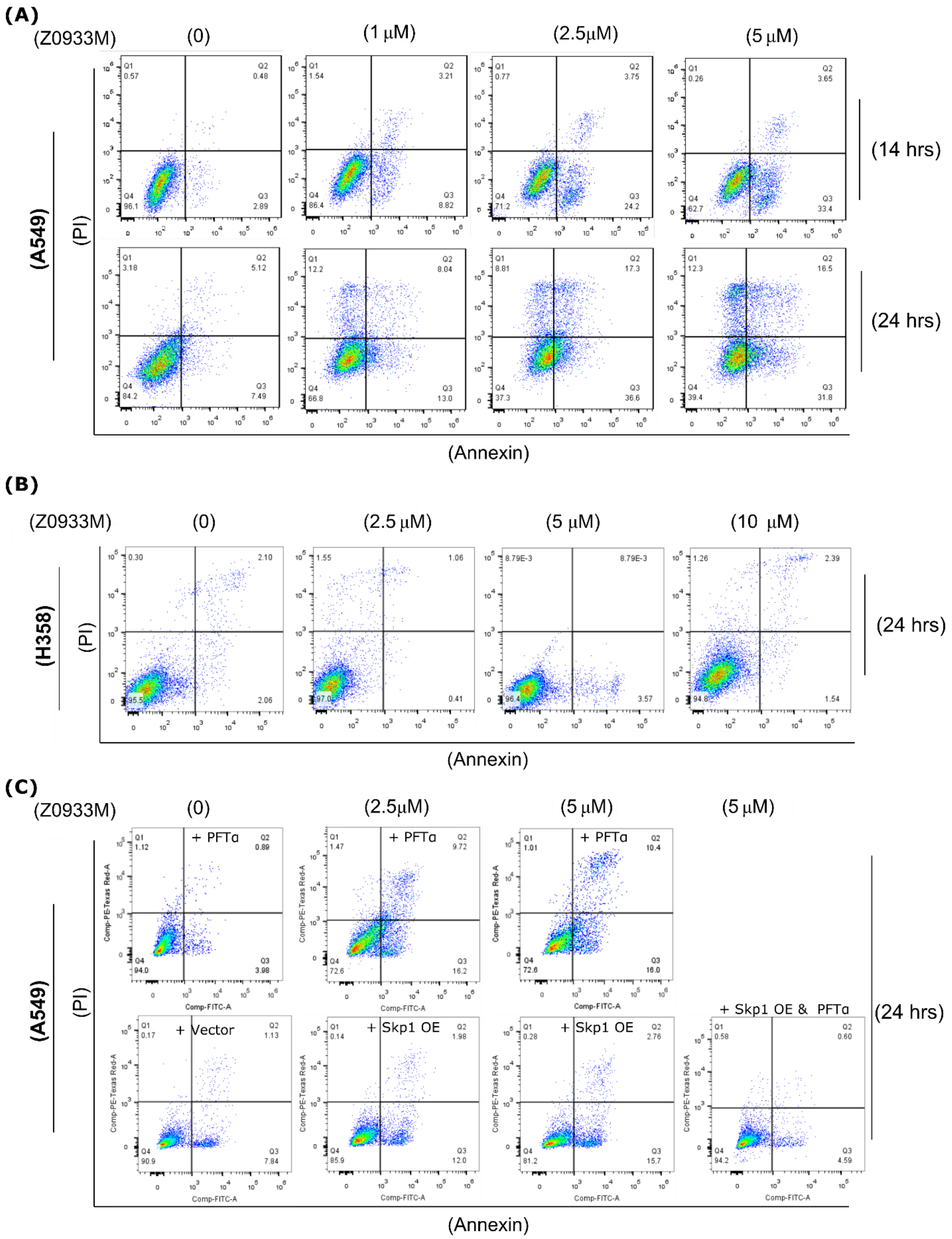

Figure S8. Flow cytometric analysis of Z0933M induced apoptosis, related to Figure 5.
(A) Dose- and time-dependent apoptotic/necrosis effect of Z0933M treatment on A549 cells.

(B) Dose-dependent apoptotic/necrosis effect of Z0933M treatment on H358 cells.
(C) Dose-dependent apoptotic/necrosis effect of Z0933M treatment on A549 cells in presence of p53 inhibitor (PFTα) and Skp1 overexpression.

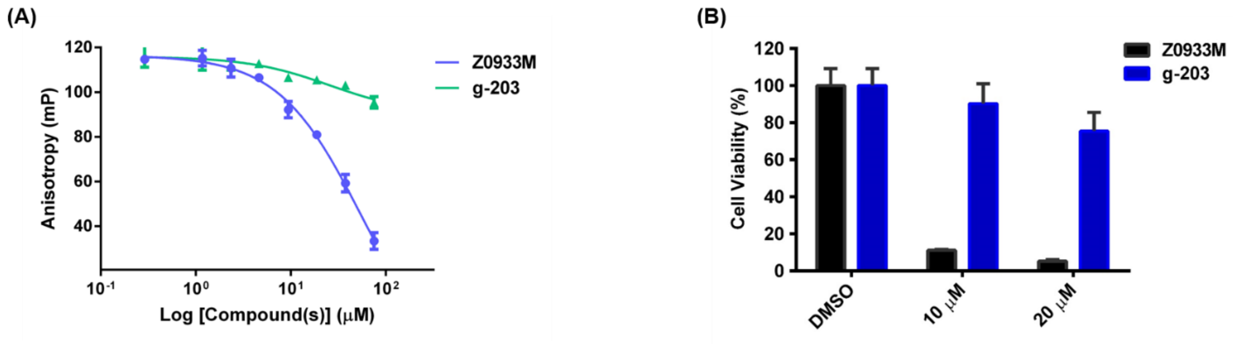

Figure S9. Functional comparison of Z0933M to its structural analogue g-203 by FP and cell viability assays, related to text in Discussion and Figures 1 and 4.
(A) *In vitro* FP assay with Z0933M and g-203
(B) Cell viability in A549 cells, comparing the potency of Z0933M and its structural analog g-203.

Table S1. List of primers used for RT-qPCR, related to Key Recourses Table and STAR Methods

Primer name	Forward sequence (5 ´-3´)	Reverse sequence (5 ´-3´)
Human *β-actin*	TTCTACAATGAGCTGCGTGTG	GGGGTGTTGAAGGTCTCAAA
Human *BAX*	TGCTACAGGGTTTCATCCAGG	TCCACGTCAGCAATCATCCT
Human *BAD*	GCACAGCAACGCAGATGC	AAGTTCCGATCCCACCAGG
Human *BCL2*	ATAACGGAGGCTGGGATGC	GGCAGGTTTGTCGACCTCA
Human *NOXA*	GGTGCCAGCAGACTTGAAGG	GCATTTCCATCAACCGGCG
Human *PUMA*	GAGCGGCGGAGACAAGAA	AGGAGTCCCATGAAGAGATTGT
Human *p21*	GGAAGACCATGTGGACCTGT	GGCGTTTGGAGTGGTAGAAA
Human *TRAIL*	GCTCTGGGCCGCAAAAT	TGCAAGTTGCTCAGGAATGAA
Human *TNFRSF10B*	TACCACGACCAGAGACACC	CACCCTGTTCTACACGTCCG

Data S1. ¹H-NMR spectrums of synthesized compounds, related to STAR Methods

JH-1

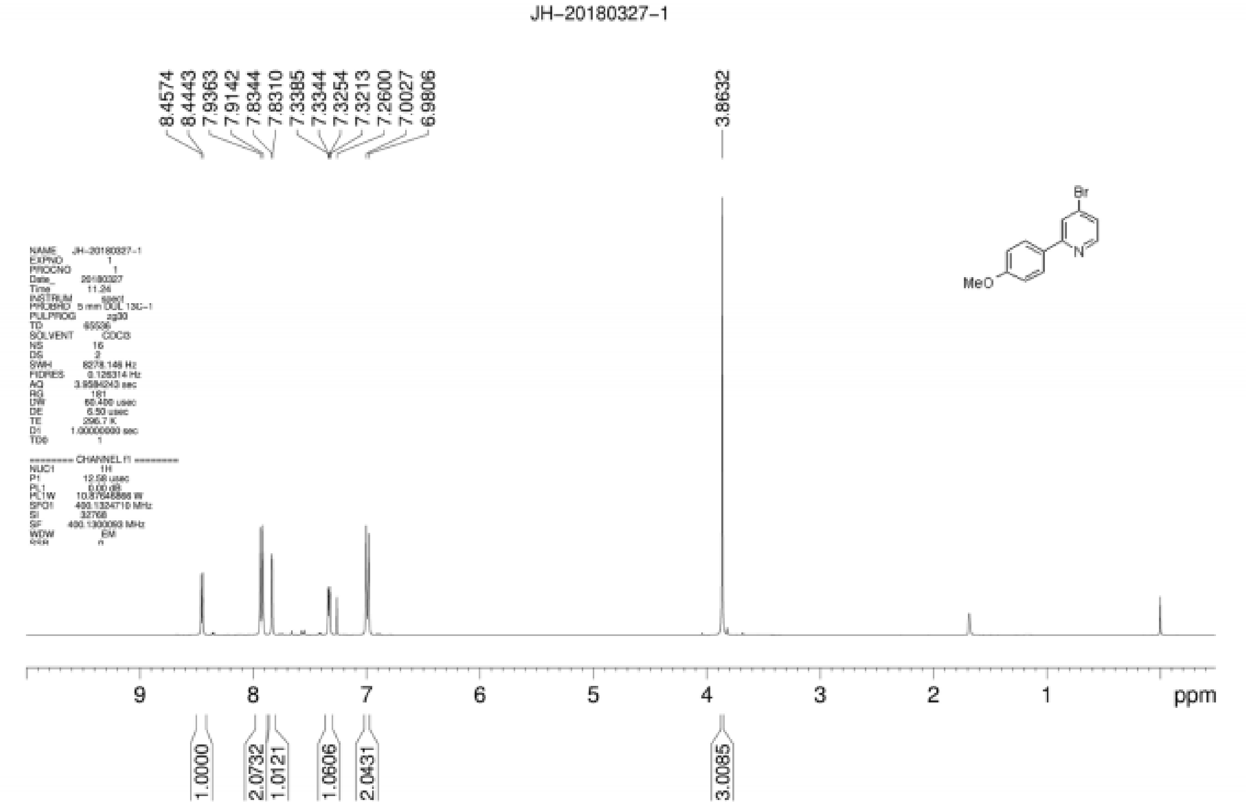

JH-2

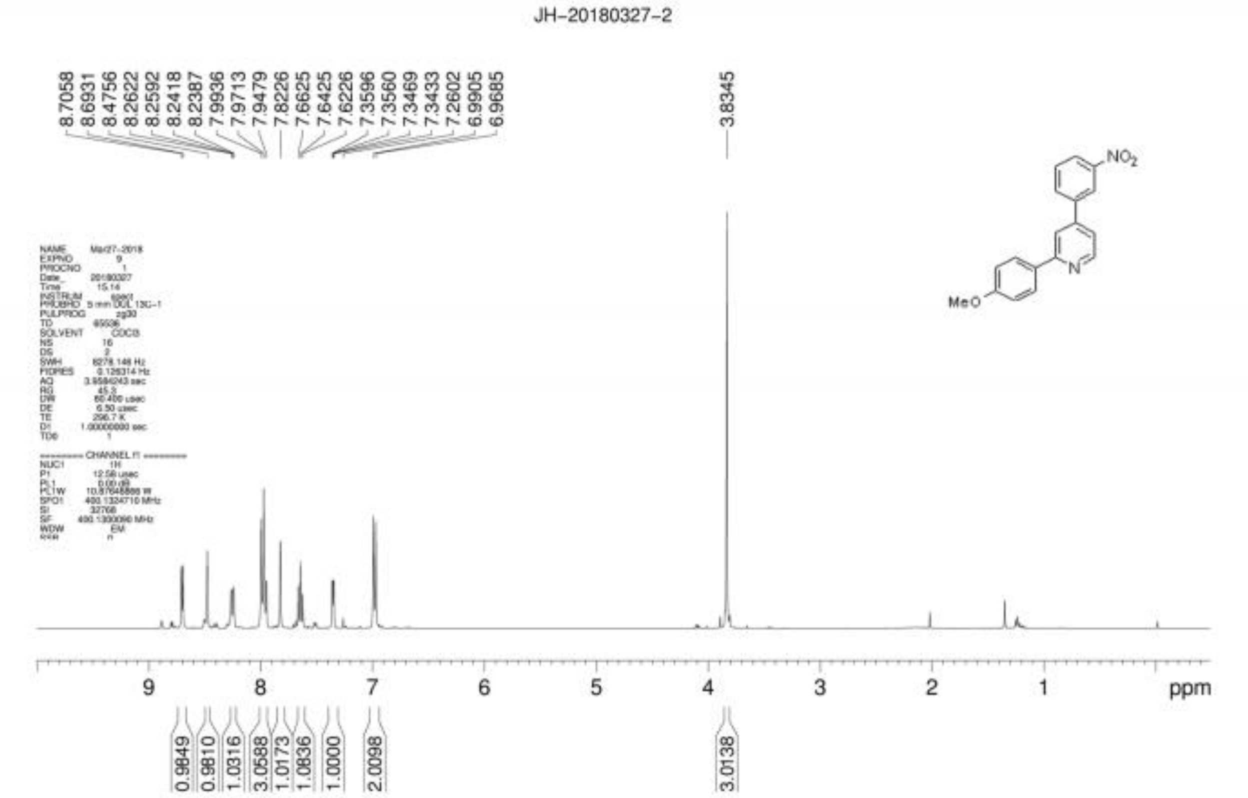
JH-20180327-2
8.7058
8.6931
8.4756
8.2622
8.2592
8.2418
8.2387
7.9936
7.9713
7.9479
7.8226
7.6625
7.6425
7.6226
7.3596
7.3560
7.3469
7.3433
7.2602
6.9905
6.9685
3.8345
NO_2
MeO
N
9
8
7
6
5
4
3
2
1
ppm
0.9849
0.9810
1.0316
3.0588
1.0173
1.0836
1.0000
2.0098
3.0138

JH-3

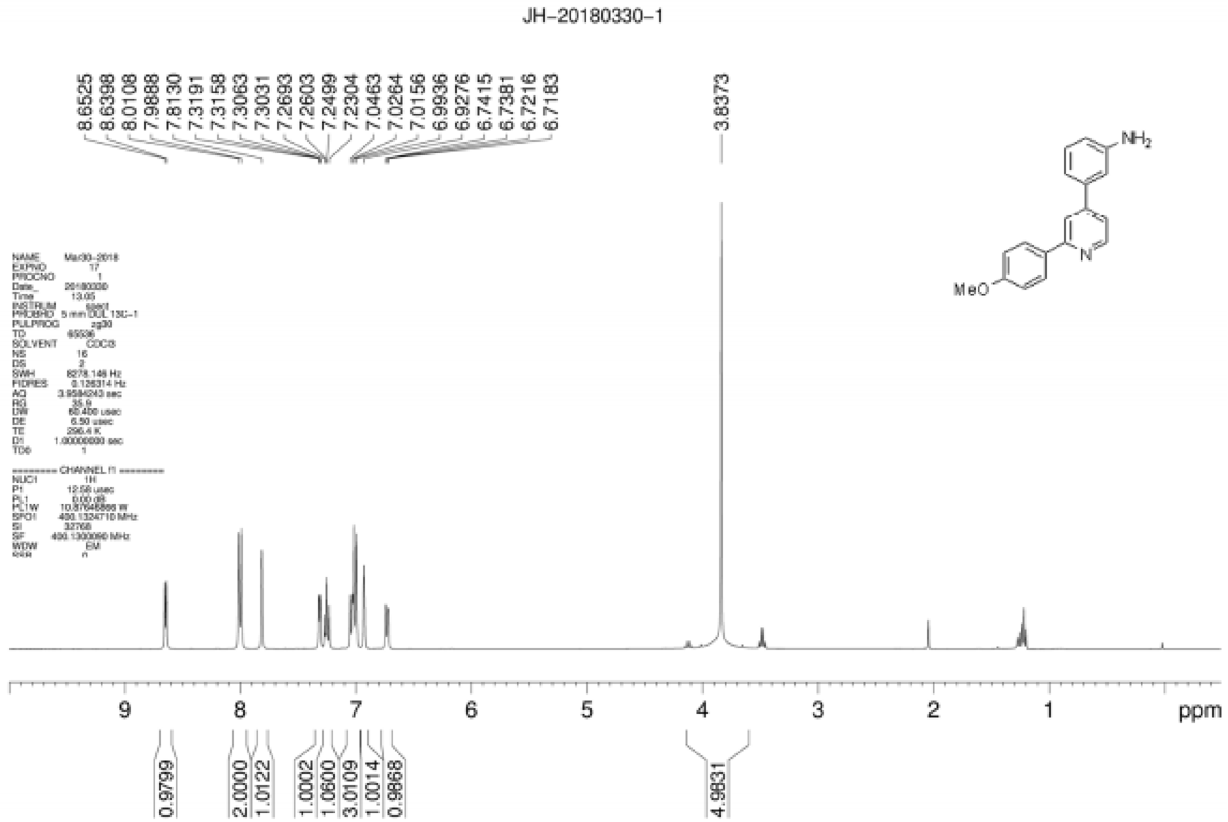
JH-20180330-1
8.6525
8.6398
8.0108
7.9888
7.8130
7.3191
7.3158
7.3063
7.3031
7.2693
7.2603
7.2499
7.2304
7.0463
7.0264
7.0156
6.9936
6.9276
6.7415
6.7381
6.7216
6.7183
3.8373
NH_2
N
MeO
9
8
7
6
5
4
3
2
1
ppm
0.9799
2.0000
1.0122
1.0002
1.0600
3.0109
1.0014
0.9868
4.9831

JH-4

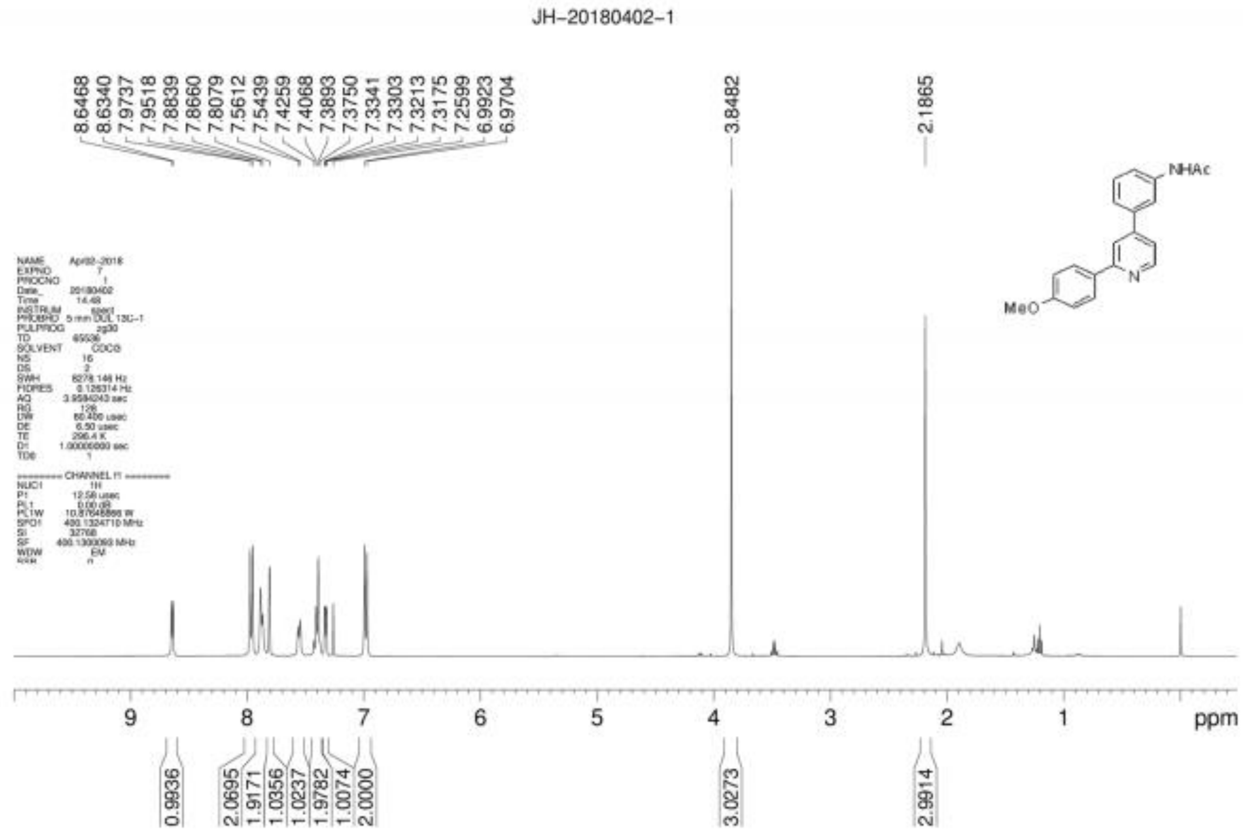
JH-20180402-1
8.6468
8.6340
7.9737
7.9518
7.8839
7.8660
7.8079
7.5612
7.5439
7.4259
7.4068
7.3893
7.3750
7.3341
7.3303
7.3213
7.3175
7.2599
6.9923
6.9704
3.8482
2.1865
NHAc
MeO
N
ppm
0.9936
2.0695
1.9171
1.0356
1.0237
1.9782
1.0074
2.0000
3.0273
2.9914

JH-5

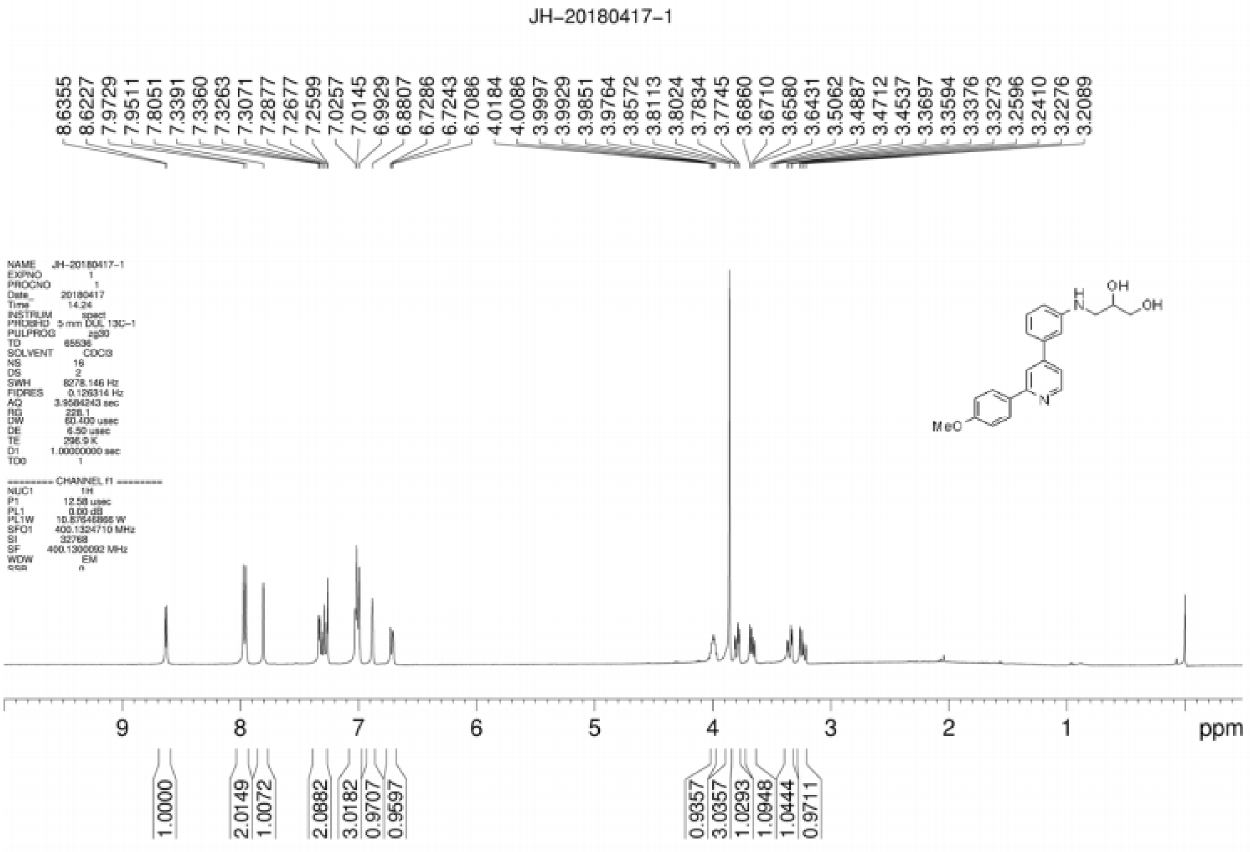

JH-20180417-1
8.6355
8.6227
7.9729
7.9511
7.8051
7.3391
7.3360
7.3263
7.3071
7.2877
7.2677
7.2599
7.0257
7.0145
6.9929
6.8807
6.7286
6.7243
6.7086
4.0184
4.0086
3.9997
3.9929
3.9851
3.9764
3.8572
3.8113
3.8024
3.7834
3.7745
3.6860
3.6710
3.6580
3.6431
3.5062
3.4887
3.4712
3.4537
3.3697
3.3594
3.3376
3.3273
3.2596
3.2410
3.2276
3.2089
ppm
1.0000
2.0149
1.0072
2.0882
3.0182
0.9707
0.9597
0.9357
3.0357
1.0293
1.0948
1.0444
0.9711
MeO
N
OH

JH-6

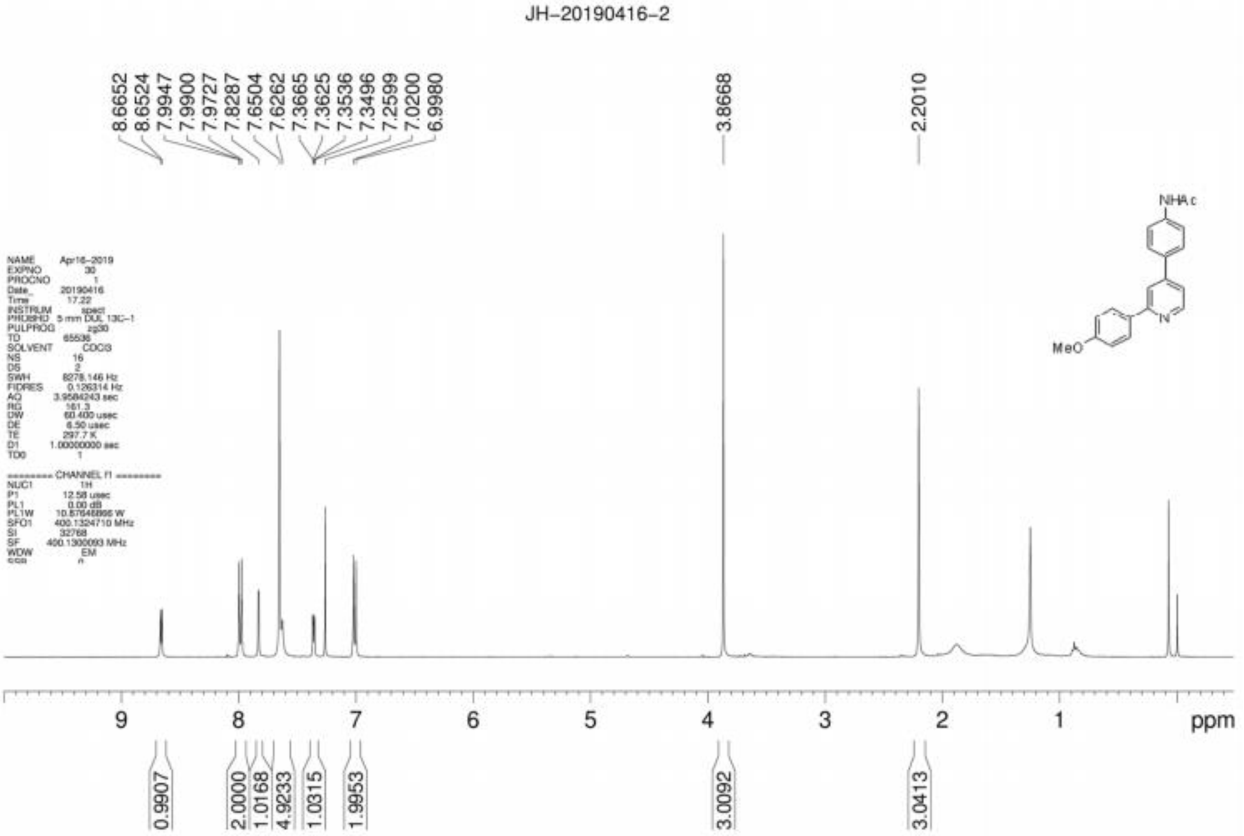

JH−20190416−2
8.6652
8.6524
7.9947
7.9900
7.9727
7.8287
7.6504
7.6262
7.3665
7.3625
7.3536
7.3496
7.2599
7.0200
6.9980
3.8668
2.2010
NHAc
MeO
N
NAME Apr16−2019
EXPNO 30
PROCNO 1
Date_ 20190416
Time 17.22
INSTRUM spect
PROBHD 5 mm DUL 13C−1
PULPROG zg30
TD 65536
SOLVENT CDCl3
NS 16
DS 2
SWH 8278.146 Hz
FIDRES 0.126314 Hz
AQ 3.9584243 sec
RG 161.3
DW 60.400 usec
DE 6.50 usec
TE 297.7 K
D1 1.00000000 sec
TD0 1
======== CHANNEL f1 ========
NUC1 1H
P1 12.58 usec
PL1 0.00 dB
PL1W 10.87646866 W
SFO1 400.1324710 MHz
SI 32768
SF 400.1300093 MHz
WDW EM
9
8
7
6
5
4
3
2
1
ppm
0.9907
2.0000
1.0168
4.9233
1.0315
1.9953
3.0092
3.0413

JH-7

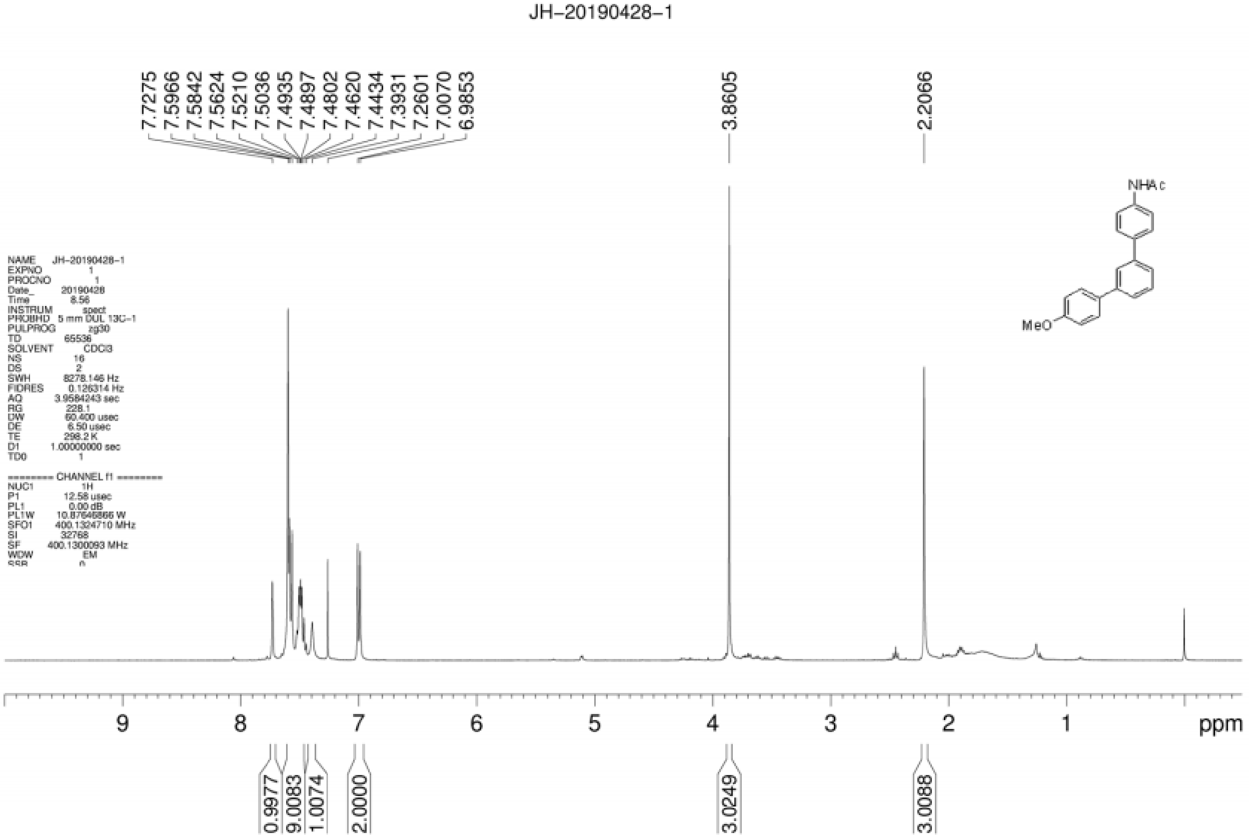
JH−20190428−1
7.7275
7.5966
7.5842
7.5624
7.5210
7.5036
7.4935
7.4897
7.4802
7.4620
7.4434
7.3931
7.2601
7.0070
6.9853
3.8605
2.2066
NHAc
MeO
9
8
7
6
5
4
3
2
1
ppm
0.9977
9.0083
1.0074
2.0000
3.0249
3.0088

JH-8

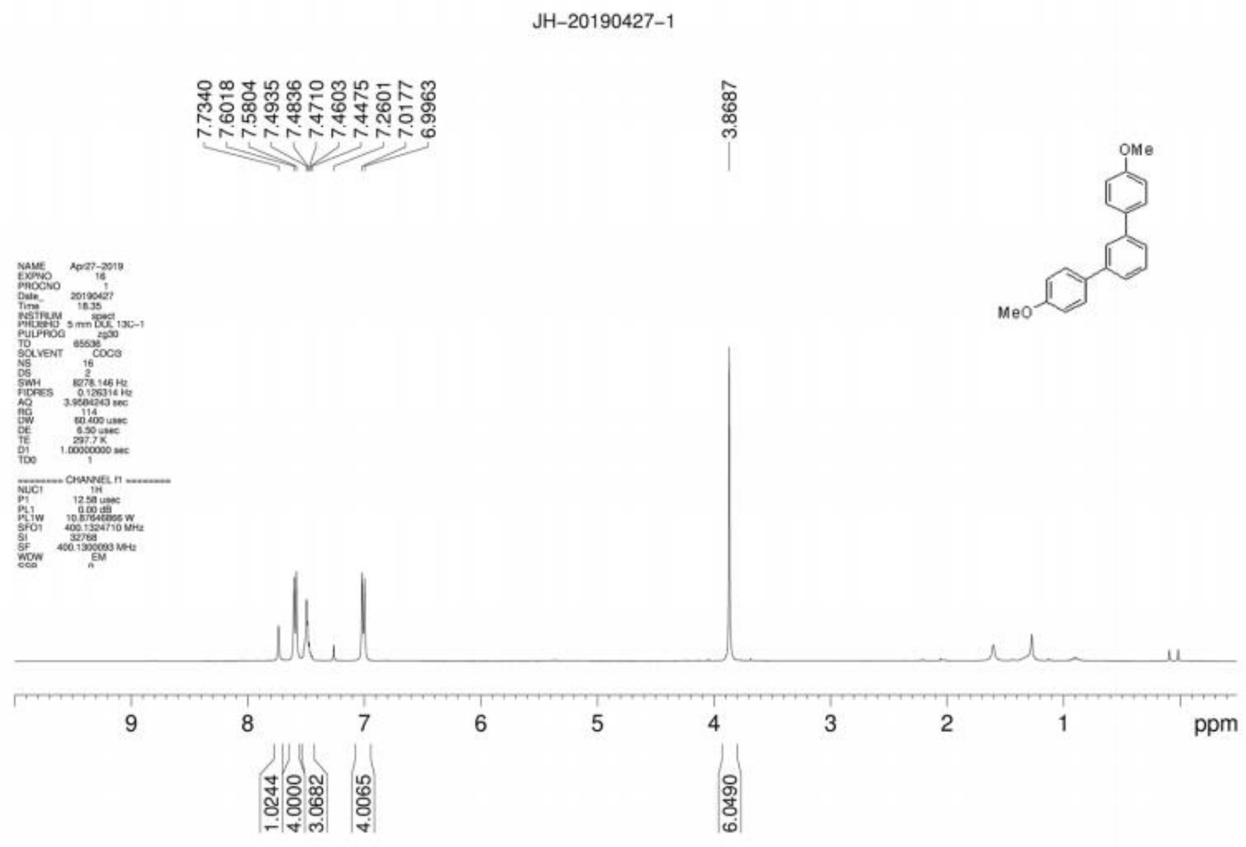

JH-20190427-1
7.7340
7.6018
7.5804
7.4935
7.4836
7.4710
7.4603
7.4475
7.2601
7.0177
6.9963
3.8687
OMe
MeO
9
8
7
6
5
4
3
2
1
ppm
1.0244
4.0000
3.0682
4.0065
6.0490

JH-9

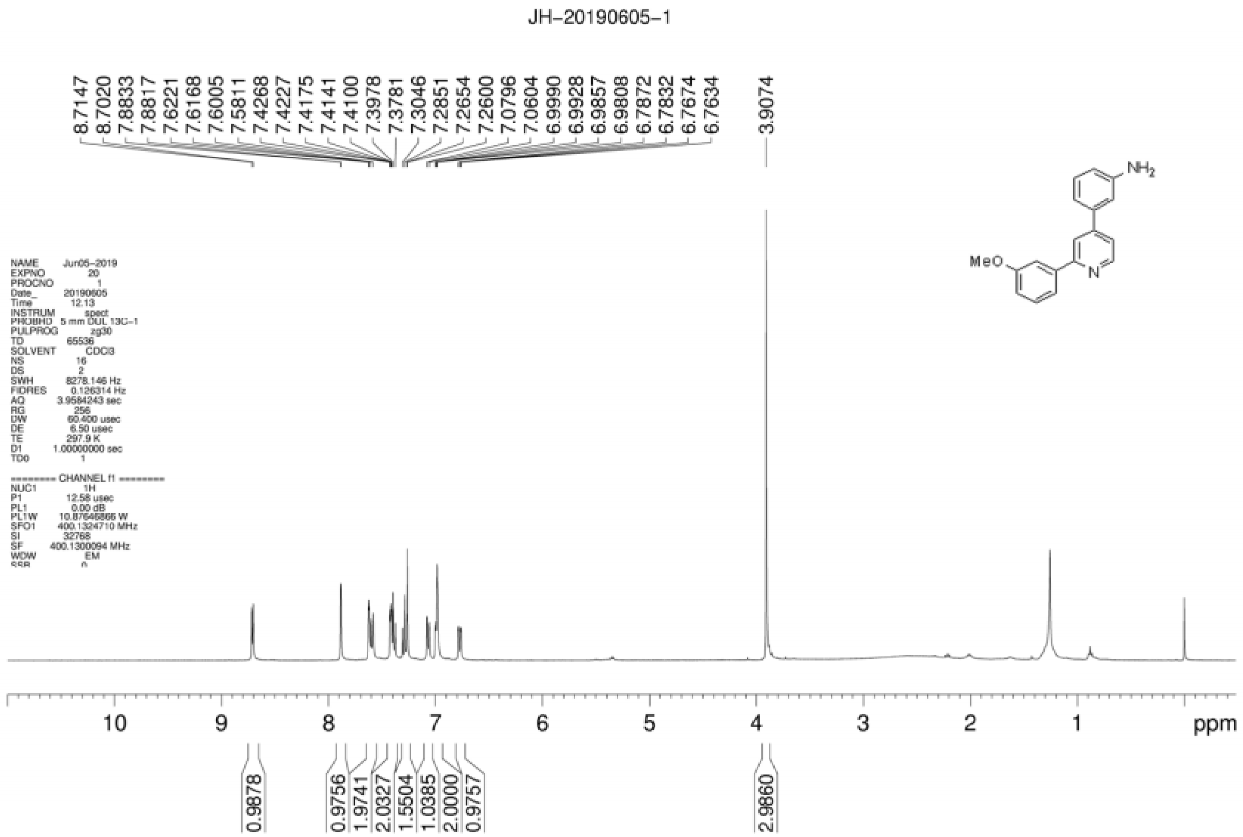
JH-20190605-1
8.7147
8.7020
7.8833
7.8817
7.6221
7.6168
7.6005
7.5811
7.4268
7.4227
7.4175
7.4141
7.4100
7.3978
7.3781
7.3046
7.2851
7.2654
7.2600
7.0796
7.0604
6.9990
6.9928
6.9857
6.9808
6.7872
6.7832
6.7674
6.7634
3.9074
NH2
MeO
N
NAME Jun05-2019
EXPNO 20
PROCNO 1
Date_ 20190605
Time 12.13
INSTRUM spect
PROBHD 5 mm DUL 13C-1
PULPROG zg30
TD 65536
SOLVENT CDCl3
NS 16
DS 2
SWH 8278.146 Hz
FIDRES 0.126314 Hz
AQ 3.9584243 sec
RG 256
DW 60.400 usec
DE 6.50 usec
TE 297.9 K
D1 1.00000000 sec
TD0 1
======== CHANNEL f1 ========
NUC1 1H
P1 12.58 usec
PL1 0.00 dB
PL1W 10.87646866 W
SFO1 400.1324710 MHz
SI 32768
SF 400.1300094 MHz
WDW EM
10
9
8
7
6
5
4
3
2
1
ppm
0.9878
0.9756
1.9741
2.0327
1.5504
1.0385
2.0000
0.9757
2.9860

JH-10

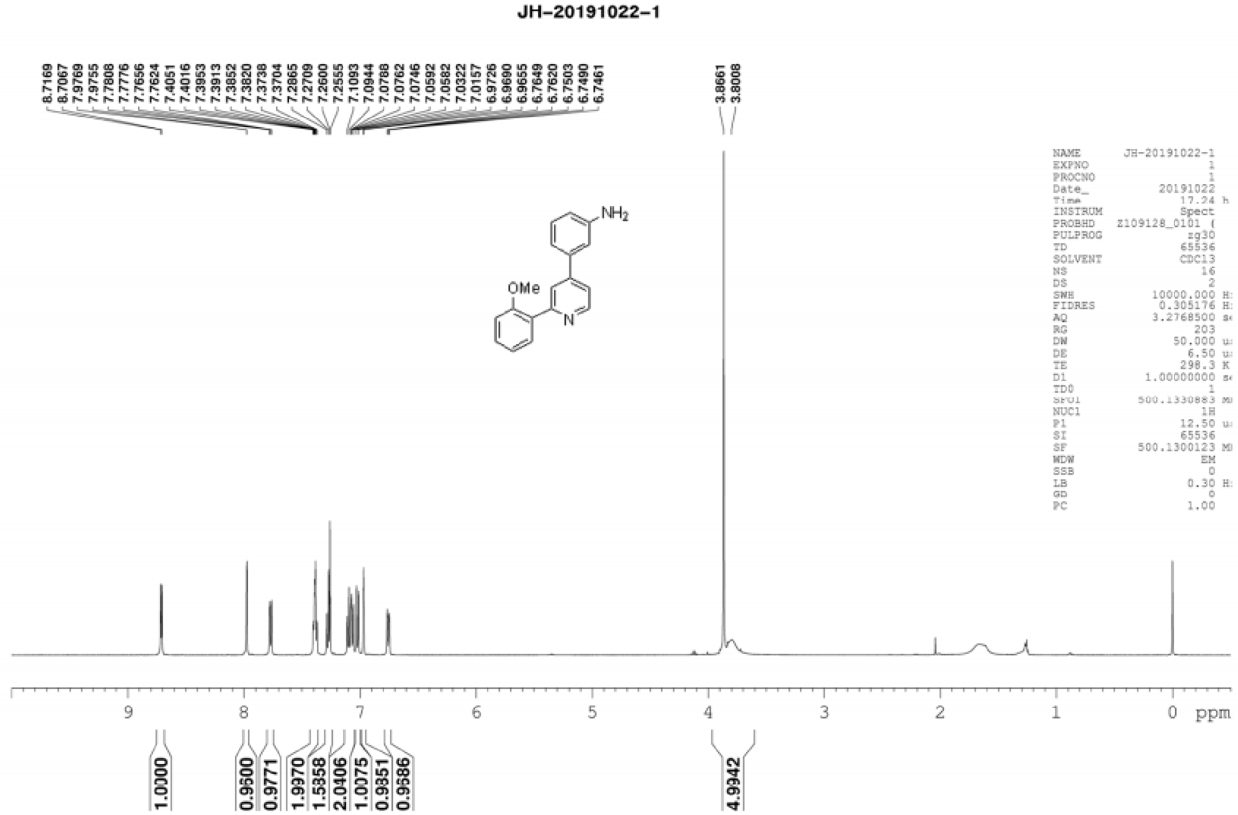

JH-12

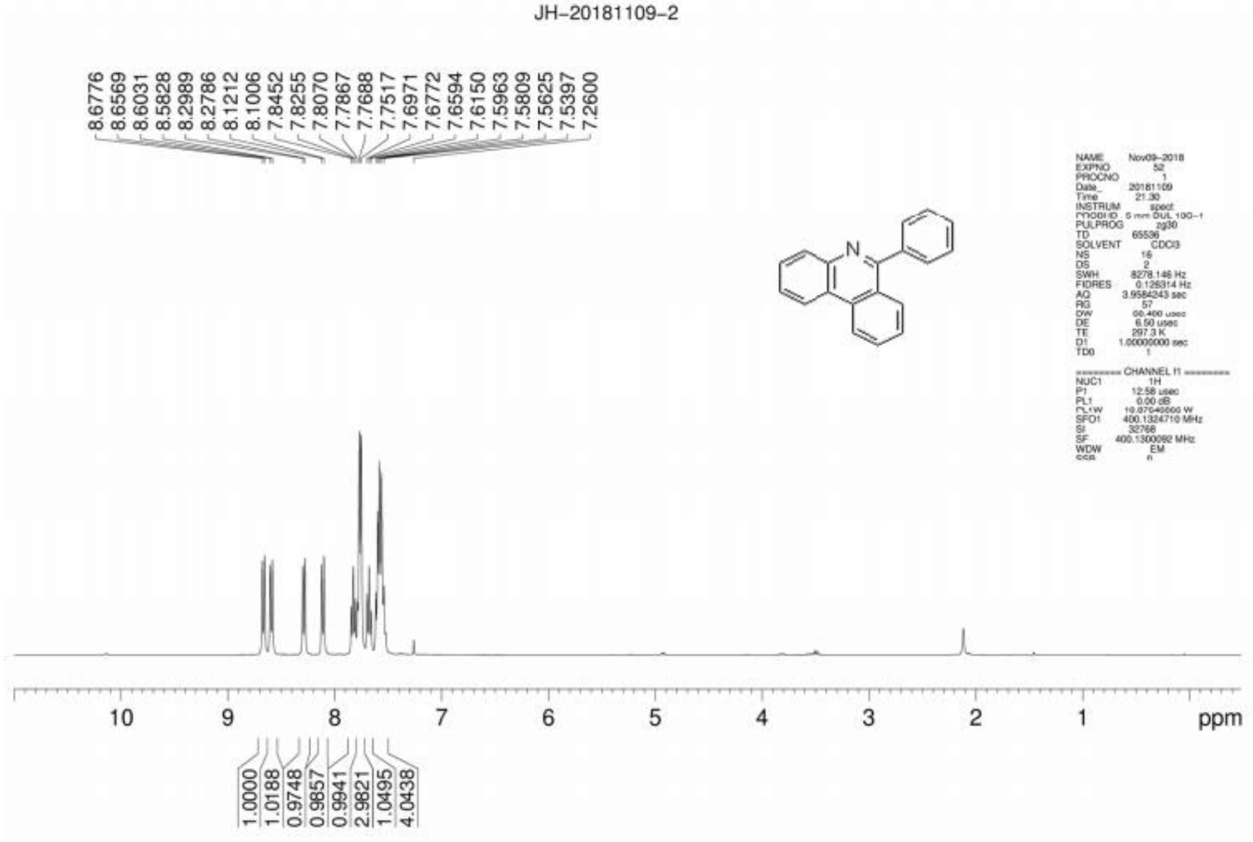

JH−20181109−2
8.6776
8.6569
8.6031
8.5828
8.2989
8.2786
8.1212
8.1006
7.8452
7.8255
7.8070
7.7867
7.7688
7.7517
7.6971
7.6772
7.6594
7.6150
7.5963
7.5809
7.5625
7.5397
7.2600
N
10
9
8
7
6
5
4
3
2
1
ppm
1.0000
1.0188
0.9748
0.9857
0.9941
2.9821
1.0495
4.0438
